# MCH-02, a synthetic macamide analog with *in vitro* FAAH-inhibitory activity, modulates hippocampal endocannabinoid system and BK channel expression without preventing PTZ-induced progressive seizure susceptibility in rats

**DOI:** 10.3389/fneur.2026.1843264

**Published:** 2026-10-16

**Authors:** Yessenia Yauri-Huaman, Rodrigo Caceres-Robles, Christell Becerra Flores, Miguel Garay-Malpartida, Carlos Medina-Saldivar, Luis F. Pacheco-Otalora

**Affiliations:** 1 Laboratorio de Investigación en Neurociencias, Instituto Científico, Universidad Andina del Cusco, Cusco, Peru; 2 Multidisciplinary Research Center, School of Arts, Sciences and Humanities, University of São Paulo, São Paulo, Brazil

**Keywords:** BK Ca channel, endocannabinoids, epilepsy—abnormalities, macamide, PTZ (pentylenetetrazol)

## Abstract

Epilepsy remains a major neurological disorder, with about 30% of patients showing drug resistance despite the availability of multiple antiseizure medications. The endocannabinoid system (ECS) has emerged as a promising therapeutic target due to its role in regulating neuronal excitability. This study examined the effects of a new macamide-derived fatty acid amide hydrolase (FAAH) inhibitor, *N*-(8Z-heptadecen-1-yl)-*O*-(3-pyridylmethyl) carbamate (MCH-02), on seizure development, cognition, and hippocampal molecular markers in a chronic pentylenetetrazol (PTZ) kindling model in rats. Behavioral measures (Racine scale, survival, Barnes maze, and novel object recognition) and molecular analyses (RT-qPCR and Western blot of CA1, CA3, and dentate gyrus) were conducted. MCH-02 did not prevent PTZ-induced progressive seizure susceptibility and initially worsened seizure severity. Survival probability did not differ significantly among groups, and we observed no evidence of a protective effect on survival. On a molecular level, MCH-02 increased *FAAH, Cnr1,* and *Napepld* mRNA levels in the CA1 region. It altered CB1 receptor and BKα1 subunit protein levels in the hippocampus, suggesting ECS remodeling and changes in potassium channel regulation. Despite these molecular changes, behavioral protection was inconsistent, suggesting a complex and context-dependent relationship between ECS function and intrinsic excitability. Overall, MCH-02 modulates molecular pathways associated with hippocampal endocannabinoid signaling and BK channel remodeling but does not demonstrate antiepileptogenic efficacy in the PTZ-kindling model. However, the surprising worsening of seizures highlights the importance of further research incorporating electrophysiological and pharmacokinetic studies to determine optimal dosing and therapeutic timing for FAAH inhibitors in chronic epilepsy models.

## 1 Introduction

Epilepsy is a chronic neurological disorder characterized by recurrent and unprovoked seizures resulting from abnormal neuronal hyperexcitability and hypersynchronous activity within brain networks. Despite significant advances in antiseizure therapies, approximately 30% of patients remain drug-resistant, highlighting a critical unmet clinical need and the importance of identifying novel mechanistic targets beyond conventional pharmacological approaches (1). Increasing evidence indicates that epilepsy should not be viewed as a static condition but rather as the outcome of a dynamic and progressive process known as progressive seizure susceptibility. This process involves coordinated molecular, cellular, and network-level alterations that gradually transform a normal brain into a hyperexcitable state (2). Mechanisms underlying progressive seizure susceptibility include synaptic plasticity changes, ion channel remodeling, neuroinflammation, and alterations in neuromodulatory systems, ultimately leading to persistent seizure susceptibility (3).

Experimental models are essential for investigating these mechanisms. Among them, the pentylenetetrazole (PTZ)-induced kindling model is widely used due to its ability to reproduce progressive seizure development through repeated subconvulsive stimulation (4, 5). Unlike acute seizure paradigms, PTZ kindling captures the temporal evolution of progressive seizure susceptibility, enabling the study of both maladaptive plasticity and compensatory responses that shape network excitability over time (6).

Among emerging neuromodulatory targets, the endocannabinoid system (ECS) has received considerable attention due to its role in regulating synaptic transmission and neuronal excitability (7). However, the ECS does not exert a uniformly anticonvulsant effect. Instead, it operates as a highly dynamic, context-dependent system that can produce both anticonvulsant and proconvulsant outcomes depending on receptor localization, neuronal circuitry, and disease stage (8). Endocannabinoids act as retrograde messengers that modulate both glutamatergic and GABAergic transmission, and this dual regulation can either stabilize or destabilize network activity depending on the underlying excitatory–inhibitory balance (9). Pharmacological modulation of the ECS has therefore been explored as a potential therapeutic strategy. In particular, inhibition of fatty acid amide hydrolase (FAAH), the enzyme responsible for anandamide degradation, represents an indirect approach to enhancing endocannabinoid signaling (10). While enhancing endocannabinoid signaling has shown anticonvulsant effects in some experimental models, its impact is not uniform and depends on the endocannabinoid pathway engaged, synaptic localization, seizure model, and the stage of progressive seizure susceptibility. In particular, endocannabinoid signaling at excitatory synapses may suppress seizures, whereas signaling at inhibitory synapses may promote seizure activity; moreover, its role may shift from pro-epileptogenic to anti-epileptogenic as the disease progresses (11, 12). These findings indicate that increased endocannabinoid tone does not necessarily translate into seizure suppression and may, under certain conditions, contribute to network instability.

Macamides, bioactive compounds derived from *Lepidium meyenii*, have been identified as natural FAAH inhibitors with potential neuromodulatory properties (13, 14). Although previous studies suggest anticonvulsant effects of FAAH inhibitors such as URB597 (15), it remains unclear whether macamide-mediated FAAH inhibition exerts similar effects during the progressive development of epilepsy. However, their effects during progressive seizure susceptibility remain poorly understood, particularly regarding their pharmacological profile and interactions with intrinsic excitability mechanisms.

In parallel, intrinsic regulators of neuronal excitability, such as large-conductance calcium-activated potassium (BK) channels, have been implicated in seizure generation and progressive seizure susceptibility (16). BK channels play a central role in action potential repolarization and in regulating firing frequency (17, 18). However, their functional contribution is complex: while they can limit excitability by promoting repolarization, they may also facilitate high-frequency firing and burst activity, potentially enhancing seizure susceptibility depending on cellular and network context (19). Furthermore, alterations in BK channel expression and function have been associated with epileptic network remodeling (20, 21).

Importantly, progressive seizure susceptibility is multifactorial and cannot be explained by modulating a single pathway. It involves synaptic reorganization, neuroinflammatory responses, gliosis, and ionic dysregulation, all of which contribute to progressive network instability (22, 23). Within this framework, both ECS signaling and BK channel activity should be considered as components of a broader regulatory network rather than isolated therapeutic targets (16, 20, 24, 25). Despite these advances, a critical knowledge gap remains regarding how FAAH inhibition-driven modulation of the ECS interacts with BK channel remodeling during progressive seizure susceptibility, and whether this interaction results in neuroprotection or, conversely, contributes to network destabilization.

Therefore, the present study aimed to evaluate the effects of the macamide-derived FAAH inhibitor N-(8Z-heptadecen-1-yl)-O-(3-pyrimidinylmethyl) carbamate (MCH-02) on seizure development, cognitive outcomes, and hippocampal molecular remodeling in a chronic PTZ-induced kindling model in rats with particular emphasis on: (i) endocannabinoid system components, and (ii) BK channel expression across hippocampal subregions. We hypothesized that FAAH inhibition by MCH-02 modulates endocannabinoid tone in a context-dependent manner, leading to differential effects on hippocampal network excitability and seizure expression across stages of PTZ-induced progressive seizure susceptibility, rather than producing a uniform anticonvulsant or antiepileptogenic effect.

## 2 Materials and methods

### 2.1 Animals

Two-month-old male Sprague–Dawley rats (*n* = 39) were raised and housed (3–5 animals per cage) in the animal facility at Universidad Andina del Cusco under controlled conditions: a temperature of 21 ± 2 °C, 40% relative humidity, and free access to water and standard rodent chow throughout the experiment. All procedures were approved in advance by the Institutional Animal Care and Use Committee (IACUC) of Universidad Andina del Cusco. During drug administration and behavioral testing, animals were transported to a separate testing room with the same environmental conditions.

### 2.2 Drugs

The vehicle consisted of grade two distilled water, polyethylene glycol 40 (PEG-40), and dimethyl sulfoxide (DMSO) in a final ratio of 18:1:1 (v/v/v). Owing to its highly lipophilic nature and limited aqueous solubility, MCH-02 (N-(8Z-Heptadecen-1-yl)-O-(3-pyrimidinylmethyl) carbamate) was initially dissolved in a pre-warmed (37 °C) PEG-40/DMSO mixture (1:1, v/v), which served as co-solvents to ensure complete dissolution and formulation stability. The stock solution was then diluted with pre-warmed grade two distilled water while maintaining the final vehicle composition. Distilled water was selected as the aqueous component of the vehicle because it is compatible with the PEG-40/DMSO co-solvent system and preserves the physicochemical stability and homogeneity of the formulation. Vehicle-treated animals received the identical PEG-40/DMSO/distilled water formulation without MCH-02, thereby controlling for any potential effects attributable to the solvent mixture. For comparison, sterile 0.9% sodium chloride (saline) was used exclusively as the vehicle for pentylenetetrazole (PTZ; Sigma-Aldrich, USA), which was freshly dissolved immediately before each administration.

### 2.3 Experimental design

To investigate the harmful effects of PTZ on progressive seizure susceptibility, spatial memory, and learning, and the potential protective effect of MCH-02, four groups were established and compared. Animals receiving vehicle and PTZ (Veh + PTZ) were compared with those receiving vehicle and saline (Veh + Saline) to evaluate the effects of PTZ. Animals treated with MCH-02 and PTZ (MCH-02 + PTZ) were used to assess whether the macamide could prevent PTZ-induced damage. Those receiving MCH-02 and saline (MCH-02 + Saline) served as controls to examine the effects of MCH-02 alone. A total of 39 male Sprague–Dawley rats participated in the study and were divided into two cohorts based on the timing of their participation. The first cohort included 22 animals assigned to Veh + Saline (n = 6), Veh + PTZ (*n* = 6), MCH-02 + Saline (*n* = 5), and MCH-02 + PTZ (*n* = 5). The second cohort comprised 17 animals assigned to the same groups: Veh + Saline (*n* = 3), Veh + PTZ (*n* = 5), MCH-02 + Saline (*n* = 4), and MCH-02 + PTZ (*n* = 5) Figure 1b. The overall experimental timeline and treatment sequence are summarized in Figure 1a.

**Figure 1:**
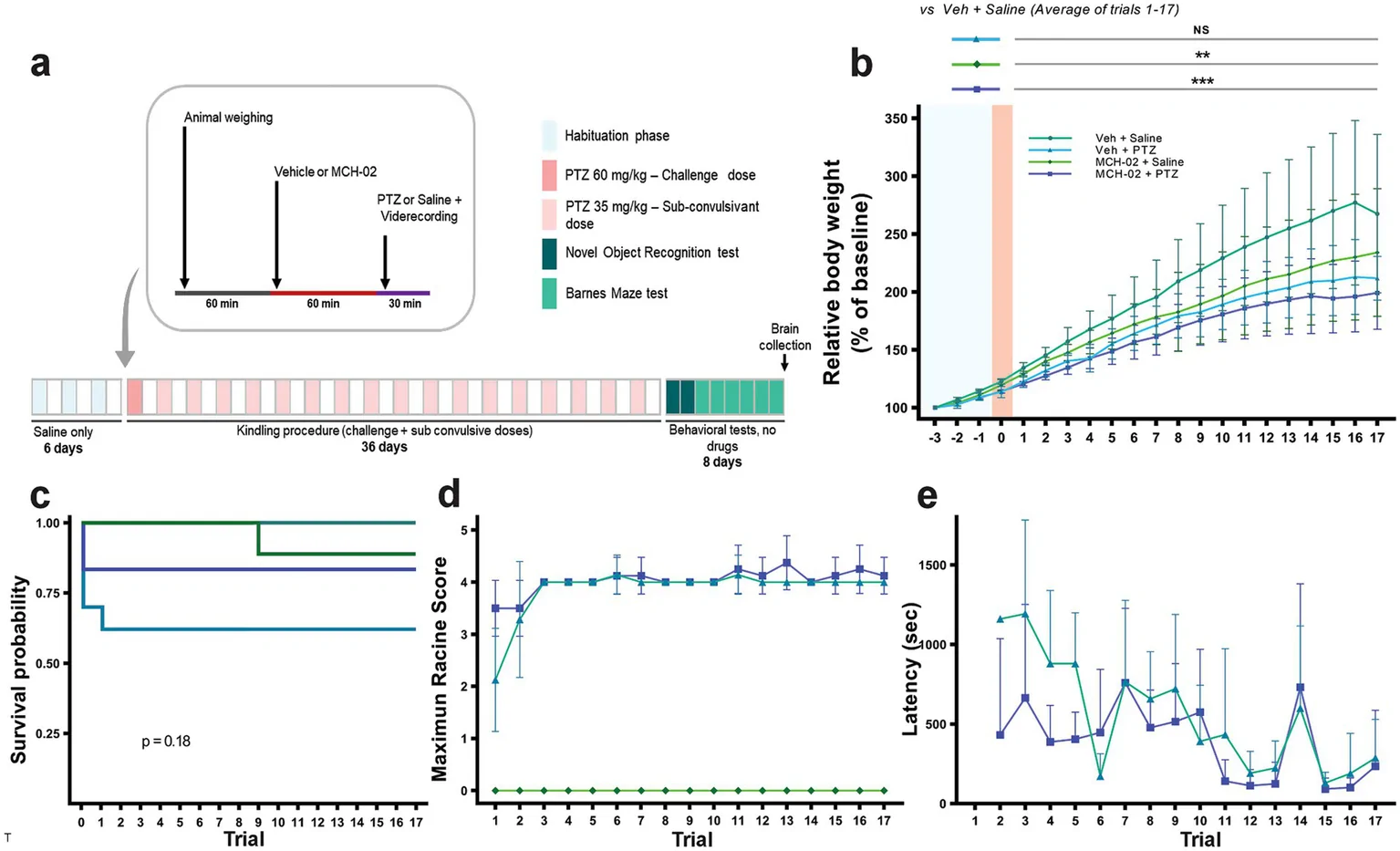
Experimental timeline, relative body weight, survival, and behavioral seizure progression during PTZ kindling. **(a)** Experimental timeline and injection sequence. Vehicle or MCH-02 (1 mg/kg, i.p.) was administered 60 min before PTZ or saline, followed by 30 min of behavioral video recording. The initial PTZ challenge dose was 60 mg/kg, followed by repeated subconvulsive doses of 35 mg/kg. Behavioral testing began 24 h after the last kindling session and comprised 2 days of novel object recognition followed by 6 days of Barnes maze assessment, with brain collection after behavioral testing on day 8. No drugs were administered during the behavioral assessment phase. **(b)** Relative body weight, calculated as (BWt/BW₀) × 100, where BW₀ is each animal’s weight at the first habituation measurement, defined as 100%. Cohorts are pooled for descriptive visualization. Annotations above the graph refer to comparisons of estimated marginal means averaged over trials 1–17 against Veh + Saline, rather than comparisons at individual trials. **(c)** Kaplan–Meier survival curves; the overall comparison among groups was not statistically significant (*p* = 0.18). **(d)** Maximum Racine score recorded for each animal during each observation session. **(e)** Latency to the onset of Racine stage 4 seizures in PTZ-treated animals. **(b,d,e)** Show mean ± SD. For **(b)**, **p < 0.01 and ***p < 0.001; NS, not statistically significant, following Tukey adjustment.

### 2.4 Pentylenetetrazol kindling

All experimental procedures were conducted in a dedicated behavioral testing room physically separated from the animal housing facility to minimize exposure to uncontrolled environmental stimuli, including noise, odors, and handling-associated stress. Chronic PTZ-induced kindling was performed according to the protocol described by Ngoupaye et al. (26), with minor modifications.

Before kindling induction, animals were habituated to the experimental environment, handling procedures, and intraperitoneal (i.p.) saline injections over three consecutive sessions. Forty-eight hours after the final habituation session, animals were transferred to the testing room and allowed to acclimate for 1 h before treatment.

To evaluate the potential disease-modifying effects of FAAH inhibition during progressive seizure susceptibility, animals received either vehicle or MCH-02 (1 mg/kg, i.p.) 1 h before each PTZ administration throughout the experimental protocol.

We determined group size *a priori* using a statistical power analysis for a one-way ANOVA with four groups (*k* = 4), performed with the pwr package (27) in R (28). Because no previous study had evaluated MCH-02 in the PTZ-kindling model, the expected effect sizes were derived from published studies that used the behavioral protocols adopted here. For the Barnes maze, the F statistic and degrees of freedom reported for escape latency by (29) were converted to η^2^ following (30), yielding η^2^ = 0.552 (Cohen’s *f* = 1.110); this value was preferred because it was the more conservative of the two available estimates [(31): η^2^ = 0.593] and because it derives from the study whose protocol was adapted in the present work. For the novel object recognition test, the *t* statistic reported by (32) for the 24-h retention interval was converted to Cohen’s *d* = 7.002 (*f* = 3.501) following (30, 33). With α = 0.001 and a target power (1 − β) of 0.80, the analysis indicated a minimum of 7 animals per group for the Barnes maze and 3 per group for the novel object recognition test; the larger, more demanding estimate was adopted. Groups were consequently planned with at least 7 animals, resulting in final group sizes of *n* = 7–8, which meet or exceed the estimated requirement. The complete derivation of the effect sizes, the parameters used, and the R code are provided in the Supplementary material (“Power analysis of behavioral tests”).

Pilot experiments evaluating MCH-02 at doses of 0.1, 1, and 10 mg/kg in an acute PTZ-induced seizure model indicated that 1 mg/kg produced the most consistent biological response without observable adverse effects. Based on these preliminary findings, we selected 1 mg/kg for all subsequent chronic PTZ-kindling experiments. This treatment schedule was designed to maintain systemic exposure to MCH-02 during the progressive development of seizure susceptibility induced by repeated PTZ stimulation. On Day 0, animals received either saline or a convulsive dose of PTZ (60 mg/kg, i.p.) administered contralaterally to the pretreatment injection. This first PTZ administration served as the challenge dose and marked the initiation of the kindling protocol. Immediately thereafter, animals were placed individually in transparent observation cages and video-recorded for 30 min to assess seizure behavior.

Forty-eight hours later, animals began receiving repeated subconvulsive PTZ injections (35 mg/kg, i.p.) every other day. Vehicle or MCH-02 pretreatment was maintained throughout the kindling period. PTZ administration continued until animals exhibited two consecutive Racine stage 5 seizures, which was considered the criterion for full kindling acquisition. Under these conditions, animals reached the fully kindled state after 17 PTZ injections over 35 days. All procedures were performed during the light phase between 08:00 and 12:00 h to minimize circadian variability.

Seizure progression was evaluated using a modified Racine scale adapted to the behavioral manifestations observed in this model: stage 1, behavioral arrest (catalepsy); stage 2, facial automatisms and neck jerks; stage 3, unilateral or bilateral forelimb clonus; stage 4, generalized myoclonic seizures involving the entire body; and stage 5, generalized tonic–clonic seizures with or without loss of postural control.

Behavioral seizure activity was quantified from the video recordings obtained after each PTZ administration. Outcome measures included relative body weight gain, latency to reach stage 4 seizures, latency and duration of stage 5 seizures, maximal Racine score achieved during each session, cumulative seizure severity, and survival rate throughout the experimental protocol.

### 2.5 Barnes maze test

All behavioral assessments were performed after completion of the 35-day PTZ-kindling protocol and following recovery from acute seizure activity. Behavioral testing was intentionally separated from the final PTZ administration to minimize potential confounding effects associated with ictal and postictal states. Consequently, the behavioral outcomes reported in this study were intended to reflect persistent neurobiological alterations associated with chronic progressive seizure susceptibility and treatment rather than transient seizure-induced impairments. To reduce potential carryover effects, cumulative stress, and task-related interference, behavioral tests were conducted sequentially from the least aversive to the most demanding paradigm.

The Barnes apparatus consisted of a circular table (120 cm in diameter) with 19 closed holes (10 cm in diameter) and one open hole leading to a goal box (12 × 36 × 10 cm) (Figure 2a). The apparatus was raised 100 cm above the floor. Four distinct signs, each in a different color, were placed on the four walls, 20 cm from the edge of the maze. A modified version of the Barnes test (29) was employed to evaluate learning and spatial memory following PTZ and MCH-02 administration. Briefly, the protocol had three parts: habituation, training, and testing days. On the habituation day, the animal was first placed directly into the goal box for 5 min. It was then positioned in the center of the maze and allowed 5 min to find the goal box. If the animal did not find the goal box, it was gently guided to it and left there for an additional 5 min. Next, the animal was placed on an opaque cylinder in the center of the maze for 1 min. After removing the cylinder, the animal had 5 min to locate the goal box. If unsuccessful, it was gently guided to the goal box and left there for 5 min. The training period lasted four consecutive days, with two trials each day. In the first trial, the animal was placed inside the opaque cylinder at the center of the maze for 1 min. After the cylinder was removed, it had 5 min to find the goal box. If it failed, it was guided to the goal box and left there for 1 min. The animal then returned to its cage to rest for 5 min. The second trial followed the same procedure: 1 min in the cylinder, 5 min to find the goal, and 1 min inside the goal box. During training, we video-recorded all trials and measured parameters such as latency to reach the goal, number of errors, total distance traveled (cm), and average velocity (cm/s). On the test day, the goal box was closed, and animals were placed in the opaque cylinder for 1 min. After the cylinder was removed, the animals were allowed to explore the maze freely. The main parameter recorded during this session was the time spent in the goal quadrant. Each trial was video-recorded and analyzed using the behavioral software EthoVision XT, version 15.

**Figure 2:**
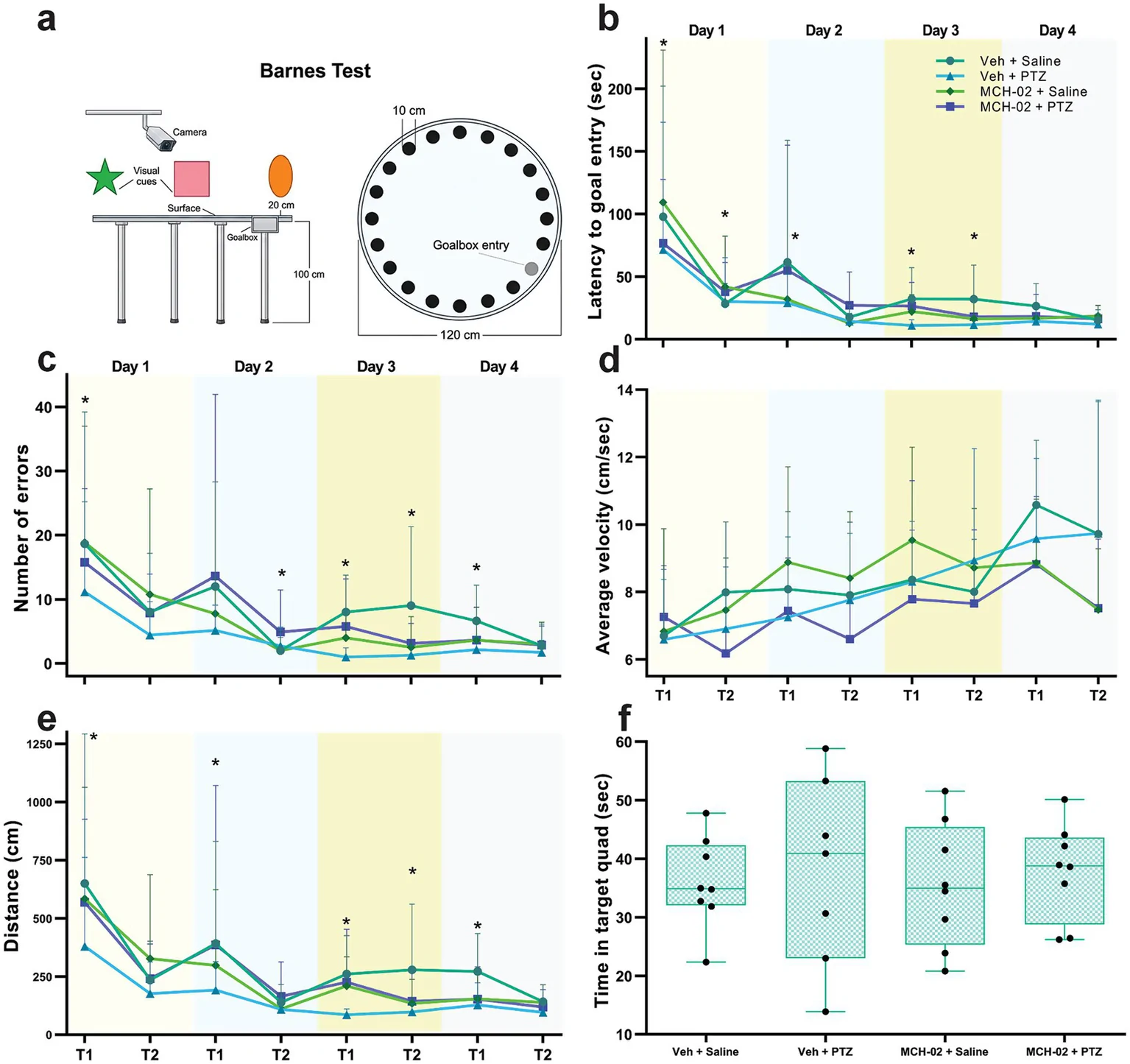
Barnes maze performance following PTZ kindling. **(a)** Schematic of the Barnes maze apparatus. During four consecutive training days, with two trials per day (T1 and T2), the following outcomes were recorded: **(b)** latency to enter the goal box, **(c)** number of errors before goal-box entry, **(d)** average velocity, and **(e)** distance traveled. **(f)** Time spent in the target quadrant during the probe test conducted the day after the final training session. **(b–e)** Show mean ± SD; panel **(f)** shows individual animals, with boxes representing the interquartile range and horizontal lines indicating medians. Group sizes were Veh + Saline, *n* = 8; Veh + PTZ, *n* = 7; MCH-02 + Saline, *n* = 8; and MCH-02 + PTZ, *n* = 8. The same animals underwent novel object recognition and Barnes maze testing. Cohorts are pooled for descriptive visualization, whereas *post hoc* comparisons were evaluated within cohort and training trial using estimated marginal means with Tukey adjustment. No significant group effect was detected for target-quadrant time during the probe test.

### 2.6 Novel object recognition test

The novel object recognition test was conducted in a 50 × 50 × 40 cm open field with dark plexiglass walls. The three-day procedure followed the methodology described by Rostami et al. and included a habituation day, a familiarization day, and a test day. Briefly, on the habituation day, animals were placed in the center of the empty open field for 10 min to explore freely, then returned to their cages. After 24 h, on the familiarization day, two identical objects matching in color, texture, shape, and size were placed 16.6 cm from one wall and 16.6 cm apart. Animals were placed in the center of the open field and allowed to explore the objects for 10 min before being returned to their cages. Finally, after another 24 h, on the test day, one familiar object was replaced with a novel object that differed in texture, color, and shape but was similar in size. Animals were again introduced to the center of the field to explore freely. To prevent bias, the positions of the familiar and novel objects were switched between trials. Each trial was video-recorded and analyzed using the behavioral software EthoVision XT, version 15. Parameters obtained from the video recordings included the time spent exploring the familiar or novel object, defined as the time the nose was within a 2 cm halo around the object, and the discrimination index (the difference between the time spent exploring the novel and familiar objects, expressed as a percentage of total exploration time).

### 2.7 Tissue processing and sampling

At the end of the experimental protocol, animals from the Saline + Vehicle (*n* = 6), PTZ + Vehicle (*n* = 5), PTZ + MCH-02 (*n* = 6), and Saline + MCH-02 (*n* = 6) groups were euthanized 35 days after treatment initiation. Tissue samples were collected from all animals that completed the PTZ-kindling protocol and consistently exhibited Racine stage 4 or 5. Consequently, molecular analyses were designed to evaluate long-term neurobiological adaptations associated with progressive seizure susceptibility and chronic MCH-02 treatment rather than immediate ictal or postictal responses. Animals were euthanized by decapitation, and the brains were rapidly removed, placed on an ice-cold surface, and immediately frozen. Following collection, brains were stored at −80 °C until cryosectioning. Cryosectioning and subsequent micropunch microdissection were performed under controlled frozen-storage conditions to preserve tissue integrity and minimize RNA and protein degradation prior to downstream molecular analyses. For hippocampal micropunch microdissection, brains were individually transferred to a cryostat (Leica CM1520, Germany) and equilibrated at −10 °C for 30 min prior to sectioning. Five consecutive coronal sections (500-μm thickness) were obtained between approximately Bregma −3.14 mm and −4.30 mm, encompassing the principal hippocampal subregions, including the dentate gyrus, CA1, and CA3 (34). Sections were mounted onto gelatin-coated microscope slides and stored at −80 °C until microdissection.

On the day of tissue collection, the cryosections were re-equilibrated in the cryostat at −20 °C. Hippocampal CA3 tissue was then bilaterally isolated under RNase-free cold conditions using an 18-gauge stainless-steel micropunch needle, following the method described by Paxinos (34). Approximately 1.5 mg of tissue was collected per animal and immediately transferred into RNase-free 1.5 mL microcentrifuge tubes kept on ice.

To maximize the use of each biological specimen and minimize inter-sample variability, each micropunched sample underwent a sequential extraction protocol that allowed both total RNA and protein to be obtained from the same tissue specimen. Briefly, samples were mechanically homogenized in 200 μL of Mammalian Lysis Buffer (50 mM Tris–HCl, pH 7.4, 150 mM NaCl, 1% NP-40, 0.5% sodium deoxycholate, 0.1% SDS, and 1 mM EDTA) supplemented with RNase and protease inhibitor cocktails. The resulting homogenate was then divided into two aliquots: 70 μL was reserved for protein extraction, while the remaining 130 μL was processed for total RNA isolation. Total RNA was isolated using TRIzol/chloroform extraction, followed by isopropanol precipitation and DNase treatment. RNA concentration and purity were determined spectrophotometrically, and integrity was verified by agarose gel electrophoresis, with intact 28S and 18S ribosomal RNA bands visualized (Supplementary Data). Purified RNA was reverse-transcribed into cDNA for RT-qPCR analyses. For protein extraction, an aliquot of the same homogenized sample was processed independently, centrifuged, and quantified using the Bradford colorimetric assay. Protein integrity was assessed by SDS-PAGE followed by Coomassie Brilliant Blue staining. All RNA, cDNA, and protein samples were stored at −80 °C until downstream molecular analyses.

This experimental design enabled evaluation of persistent molecular remodeling in the hippocampal CA3 subregion following completion of the kindling process, thereby providing insight into long-term mechanisms underlying PTZ-induced progressive seizure susceptibility and the effects of chronic MCH-02 treatment.

### 2.8 RT-qPCR

After sample extraction, total RNA was purified using the TURBO DNA-free kit (Thermo Fisher Scientific, USA) and then reverse transcribed using the High-Capacity cDNA Reverse Transcription Kit (Applied Biosystems, USA). Forward and reverse primers for the target genes (Cnr1, FAAH, Napepld, and Kcnma1) and reference genes (Hprt1 and Gapdh) were synthesized by Macrogen (Seoul, South Korea). Additional details about the oligonucleotide sequences are available in Supplementary Data 1. RT-qPCR was conducted in triplicate on a QuantStudio 3 qPCR System (Thermo Fisher Scientific, USA) with BlasTaq™ 2X PCR MasterMix (ABM, Canada). Fold change in gene expression was calculated using the geometric mean method described by (35).

### 2.9 Western blot

Total protein quantification was performed using the spectrophotometric Bradford method. All samples were diluted and aliquoted to a final concentration of 2 μg/μL. Western blot analysis was conducted with 10 μg of total protein per sample. The primary antibodies used were anti-CB1 (1:500), anti-FAAH (1:500), anti-BK channel (1:500), and anti-Tubulin (1:500). For secondary antibody incubation, species-specific secondary antibodies were used at a 1:1000 dilution. Bands were detected using a colorimetric method with the ABC kit (PK-6200, Vector Laboratories) and DAB reagent (SK-4100, Vector Laboratories). Membranes were digitized using a ChemiDoc MP Imaging System (Bio-Rad, U.S.), and band analysis was carried out with Image Lab Software (version 6.1).

### 2.10 Statistical analysis

A generalized linear mixed model (GLMM) approach was used to analyze all data except for survival probability and kindling progression. This method was selected to avoid potential bias from fitting data under the assumptions of traditional parametric methods and to enable flexibility in modeling the dependent variable with distributions such as the gamma distribution when appropriate (36). First, the data were fitted to a GLMM using either a Gaussian or a gamma distribution, depending on the Akaike information criterion (AIC), with animal ID included as a random effect. Model assumptions were then checked and validated using residual diagnostics for hierarchical models (DHARMa), considering the Kolmogorov–Smirnov test, dispersion test, and outlier test, which were deemed acceptable if *p* > 0.05. Next, independent variables and their interactions were analyzed, and, if necessary, *post hoc* pairwise comparisons were performed using estimated marginal means (EMMs) with Tukey-adjusted *p*-values. For survival probability, a Kaplan–Meier model was used, and ordinal data on kindling progression were analyzed using a cumulative link mixed model (Supplementary Data). For all statistical tests, *p* < 0.05 was considered significant. Statistical analyses were conducted, and plots were created using R (version 4.5.0) and RStudio (version 2025.05.0 + 496).

## 3 Results

### 3.1 MCH-02 did not prevent pentylenetetrazol kindling progression

Body weight was monitored as an indirect indicator of general physiological condition and expressed relative to each animal’s weight at the first habituation measurement, defined as 100% (Figure 1b). Relative body weight generally increased during follow-up. In the revised longitudinal mixed-effects analysis, no comparison between MCH-02 + PTZ and Veh + PTZ reached statistical significance at any measurement occasion in either cohort after Holm adjustment across all 80 comparisons (all adjusted *p* = 1.000 in cohort K1; all adjusted *p* ≥ 0.557 in cohort K2). Under saline conditions, relative body weight was lower in the MCH-02 group than in the vehicle group in K1 at measurement occasions −2, −1, 0, 1, and 3–16 (Holm-adjusted *p* < 0.05), but not at occasions 2 or 17. No corresponding comparison reached significance in K2. Because differences in K1 were already present during habituation, they cannot be attributed solely to treatment. Nonsignificant comparisons should not be interpreted as evidence of equivalence. Kaplan–Meier curves showed numerically lower survival in the Veh + PTZ group than in the Veh + Saline group (Figure 1c). However, the overall survival comparison was not statistically significant [χ^2^ (3) = 4.90, *p* = 0.18]. These results do not establish a survival benefit of MCH-02. Kindling progression was assessed using the maximum Racine score attained by each animal in each trial (Figure 1d). Cumulative link mixed-model analysis identified a significant group effect [χ^2^ (1) = 6.68, *p* = 0.0097], whereas neither the trial effect [χ^2^ (16) = 6.51, *p* = 0.9816] nor the group × trial interaction (*p* = 0.9955) was significant. *Post hoc* comparisons identified a higher Racine score in the MCH-02 + PTZ group than in the Veh + PTZ group during the first trial (*p* = 0.0097); no significant between-group differences were detected in subsequent trials. Thus, MCH-02 did not attenuate the progression of behavioral seizure severity under the conditions examined. Latency to the onset of Racine stage 4 seizures varied across trials (Figure 1e). The analysis identified a significant trial effect [Wald χ^2^ (15) = 56.47, *p* = 1.008 × 10^−6^], but neither the group effect [Wald χ^2^ (1) = 0.13, *p* = 0.7136] nor the group × trial interaction [Wald χ^2^ (15) = 18.43, *p* = 0.2405] was significant. These results provide no evidence that MCH-02 altered stage 4 seizure latency relative to vehicle. Racine stage 5 seizures occurred inconsistently across trials, including within individual animals, and their latency and duration were highly variable. Consequently, insufficient observations were available for inferential analysis of these outcomes.

### 3.2 Neither PTZ nor MCH-02 altered the learning and memory of rats

During the familiarization day of the novel object recognition test, the amount of time animals spent exploring each of the two identical objects was not affected by group [Wald (3) = 2.6252; *p* = 0.4531], cohort [Wald (1) = 0.7466; *p* = 0.3875], or object position [Wald (1) = 0.4692; *p* = 0.4934], indicating no significant place or object bias at the start of the experiment (Figure 3a). On the test day, the total time animals explored both objects (familiar plus novel) was similar to the time spent exploring the two identical objects during familiarization [Wald (1) = 3.7465; *p* = 0.05292]. This was unaffected by group [Wald (3) = 3.4509; *p* = 0.32719] or cohort [Wald (1) = 0.0341; *p* = 0.85341] (Figure 3b). No differences were found between the time spent exploring the familiar and the novel object on the test day [Wald (1) = 0.0236; *p* = 0.87780], and these times were not influenced by group [Wald (3) = 4.0963; *p* = 0.25126] or cohort [Wald (1) = 3.4102; *p* = 0.06480] (Figure 3c). However, although animals spent the same total time exploring both objects, the discrimination index showed a significant interaction between cohort and test day [Wald (1) = 5.6755; *p* = 0.01720]. *Post hoc* analysis revealed a significant and positive increase in the discrimination index for the MCH-02 + PTZ group compared to the Veh + PTZ group on the test day (*p* = 0.0487). Because this difference was restricted to cohort 1 and was not reproduced in cohort 2, it was interpreted as a cohort-specific effect rather than evidence of a general treatment-related improvement in recognition memory. This difference was only observed in the first cohort (Figure 3d). Additionally, there were no significant differences in total distance traveled or mean velocity among groups, cohorts, or days (Figures 3e,f).

**Figure 3:**
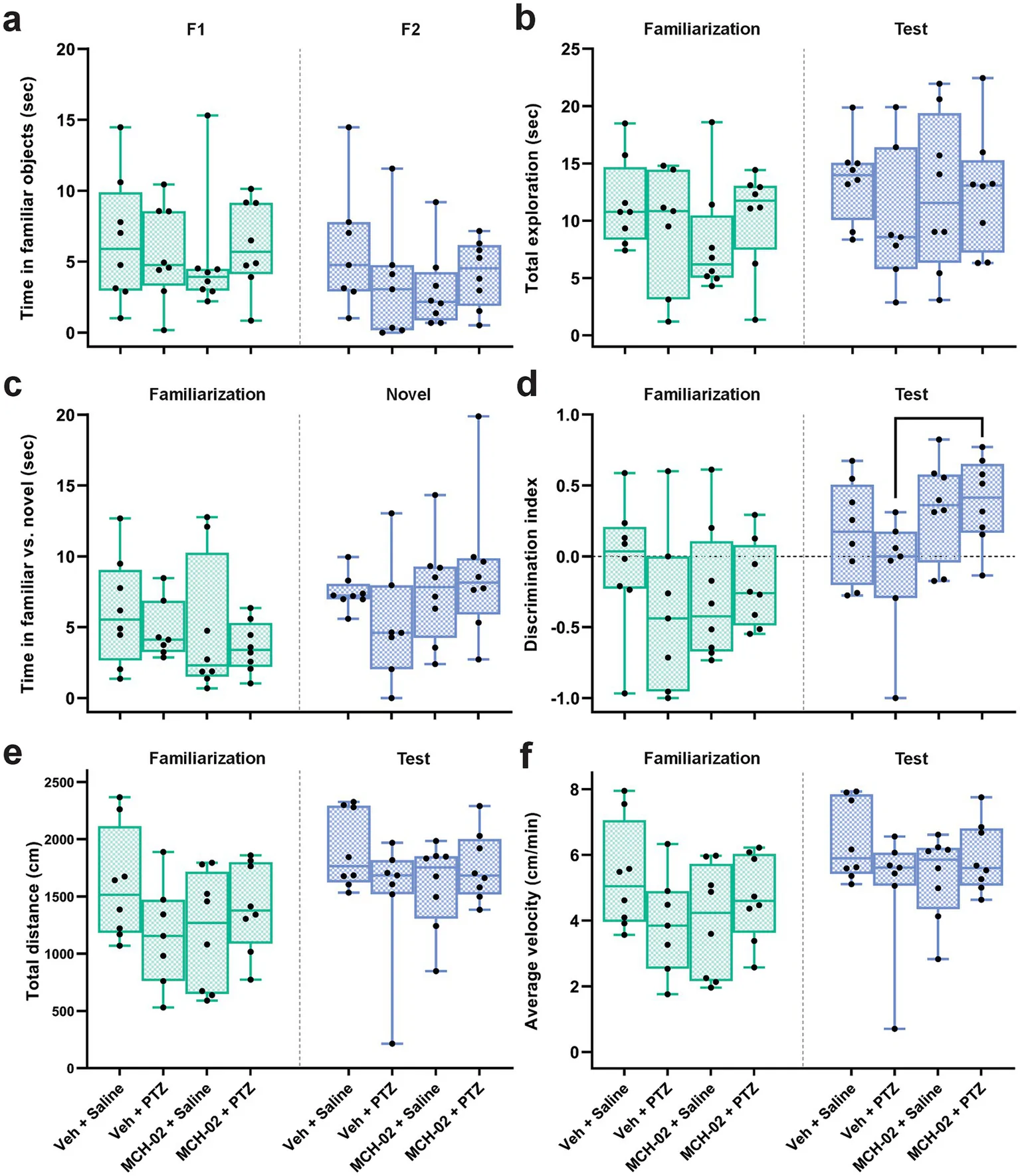
Novel object recognition and exploratory behavior following PTZ kindling. **(a)** Exploration time for the two identical objects, F1 and F2, during familiarization. **(b)** Total object exploration time during familiarization and testing. **(c)** Exploration time for the familiar and novel objects during testing. **(d)** Discrimination index during familiarization and testing. The index is displayed on a − 1 to 1 scale, with zero indicating equal exploration of the two objects. **(e)** Total distance traveled and **(f)** average velocity during familiarization and testing. Boxes represent the interquartile range, horizontal lines indicate medians, and individual points represent animals. Group sizes were Veh + Saline, *n* = 8; Veh + PTZ, *n* = 7; MCH-02 + Saline, *n* = 8; and MCH-02 + PTZ, *n* = 8. Data from both cohorts are pooled for descriptive visualization. The significant comparison between MCH-02 + PTZ and Veh + PTZ in the test phase was restricted to cohort 1 (Tukey-adjusted *p* = 0.0487) and was not reproduced in cohort 2.

During the Barnes test training phase, the time to enter the goal box decreased, reaching a stable, similar latency across groups by around day 4 (Figure 2b). The group influenced this decrease [Wald (3) = 18.9995; *p* = 0.0002735], cohort [Wald (1) = 7.5829; *p* = 0.0058923], and timepoint [Wald (7) = 15.5354; *p* = 0.0297171]. Significant interactions included group and cohort [Wald (3) = 26.9869; *p* = 5.925e-06], group and timepoint [Wald (21) = 54.2096; *p* = 9.208e-05], cohort and timepoint [Wald (7) = 17.6882; *p* = 0.0134588], and group, cohort, and timepoint [Wald (21) = 55.9842; *p* = 5.074e-05]. *Post hoc* analysis showed that, depending on the time point, animals treated with Veh + PTZ tended to have shorter latencies compared to other groups. On day 1 – trial 1, the MCH-02 + Saline group had higher latency than Veh + Saline (cohort 1, *p* = 0.0080; cohort 2, *p* = 0.0255), Veh + PTZ (cohort 1, *p* = 0.0002), and MCH-02 + PTZ (cohort 1, *p* = 0.0078). On day 1 – trial 2, the MCH-02 + Saline group still showed higher latency than the Veh + PTZ group (cohort 1, *p* = 0.0435). On day 2 – trial 1, the Veh + Saline group had higher latency than the MCH-02 + PTZ group (cohort 1, *p* = 0.0006), and the MCH-02 + PTZ group had higher latency than the MCH-02 + Saline group (cohort 2, *p* = 0.0444). On day 3, the Veh + Saline group showed higher latency than the Veh + PTZ group in both trials (cohort 1, *p* = 0.0224; cohort 2, *p* = 0.0487). By day 4, there were no significant differences among groups. The number of errors decreased over time across all groups. However, the Veh + Saline control group temporarily increased errors in session 3 but returned to levels similar to those of the other groups by session 4 (Figure 2c). Significant interactions included group and cohort [Wald (3) = 8.2850; *p* = 0.04048] and group and timepoint [Wald (21) = 34.8292; *p* = 0.02948]. *Post hoc* analysis revealed more errors in the MCH-02 + Saline group versus Veh + PTZ on day 1 – trial 1 (cohort 1, *p* = 0.0447), in MCH-02 + PTZ versus MCH-02 + Saline on day 2 – trial 2 (cohort 2, *p* = 0.0263), in Veh + Saline versus Veh + PTZ on day 3 (cohort 1, *p* = 0.0197), and in Veh + Saline versus MCH-02 + PTZ on day 4 – trial 1 (cohort 1, *p* = 0.0358). Distance traveled during each trial tended to decrease over time across all groups (Figure 2d). This measure was significantly affected by group [Wald (3) = 7.9009; *p* = 0.04810], as well as by interactions between group and cohort [Wald (3) = 8.5762; *p* = 0.03549], group and timepoint [Wald (21) = 34.5566; *p* = 0.03156], and group, cohort, and timepoint [Wald (21) = 33.0982; *p* = 0.04514]. *Post hoc* analysis showed that the distance traveled was notably higher in the MCH-02 + Saline group compared to Veh + PTZ on day 1 – trial 1 (cohort 1, *p* = 0.0256), in MCH-02 + PTZ versus Veh + Saline on day 2 – trial 1 (cohort 1, *p* = 0.0017), in Veh + Saline versus Veh + PTZ on day 3 – trial 1 (cohort 1, *p* = 0.0136) and trial 2 (cohort 1, *p* = 0.0301), and in Veh + Saline versus MCH-02 + PTZ on day 4 – trial 1 (cohort 1, *p* = 0.0236). Regarding average velocity during training, it tended to increase over time (Figure 2e) and was significantly affected by interactions between group and experiment [Wald (3) = 10.4287; *p* = 0.01525] and group, cohort, and timepoint [Wald (21) = 34.9068; *p* = 0.02891]. *Post hoc* analysis identified a significant difference on day 4 (trial 2), where the Veh + Saline group showed higher average velocity than the MCH-02 + PTZ group (cohort 2, *p* = 0.0089). The day after training, animals were tested in the same apparatus, with the goal box removed. There was no significant effect of group, cohort, or their interaction on the time spent in the quadrant where the goal box used to be (Figure 2f).

### 3.3 MCH-02 exerts endocannabinoid-related mRNA level changes mainly in hippocampal CA1 independently of PTZ administration

In CA1, the mRNA levels of all evaluated transcripts were significantly affected by the group variable (Figure 4a). FAAH mRNA levels showed a significant effect of group [Wald (3) = 16.23; *p* = 0.001017], with *post hoc* analysis revealing a notable increase in the MCH-02 + PTZ group compared to Veh + Saline (*p* = 0.0075) and Veh + PTZ (*p* = 0.0323) groups. Similarly, Cnr1 mRNA levels were influenced by group [Wald (3) = 20.499; *p* = 0.0001337], with higher expression in the MCH-02 + PTZ group relative to Veh + Saline (*p* = 0.0090) and Veh + PTZ (*p* = 0.0034). Regarding Napepld mRNA levels (group effect [Wald (3) = 24.7324; *p* = 1.756e-05]), *post hoc* analysis indicated that the MCH-02 + Saline group had elevated Napepld mRNA compared to Veh + Saline (*p* = 0.0473) and Veh + PTZ (*p* = 0.0078). The MCH-02 + PTZ group also showed higher Napepld mRNA levels than Veh + Saline (*p* = 0.0248) and Veh + PTZ (*p* = 0.0041), though there was no significant difference between the MCH-02 + PTZ and MCH-02 + Saline groups. Changes in Kcnma1 mRNA levels (group effect [Wald (3) = 17.6314; *p* = 0.0005239]) were mainly due to increased transcript levels in the MCH-02 + Saline group compared to Veh + Saline (*p* = 0.045) and Veh + PTZ (*p* = 0.012), as well as in the MCH-02 + PTZ group compared to Veh + PTZ (*p* = 0.0449). In CA3, despite observed variations, FAAH, Cnr1, and Kcnma1 mRNA levels did not differ significantly among groups (Figure 4b). The only gene transcript notably affected by the group was Napepld mRNA [Wald (3) = 14.826; *p* = 0.001971], with *post hoc* analysis confirming decreased Napepld expression in the MCH-02 + Saline (*p* = 0.0407) and MCH-02 + PTZ (*p* = 0.0143) groups. No significant changes were observed in the abundance of any tested gene transcripts in the hippocampal dentate gyrus (Figure 4c).

**Figure 4:**
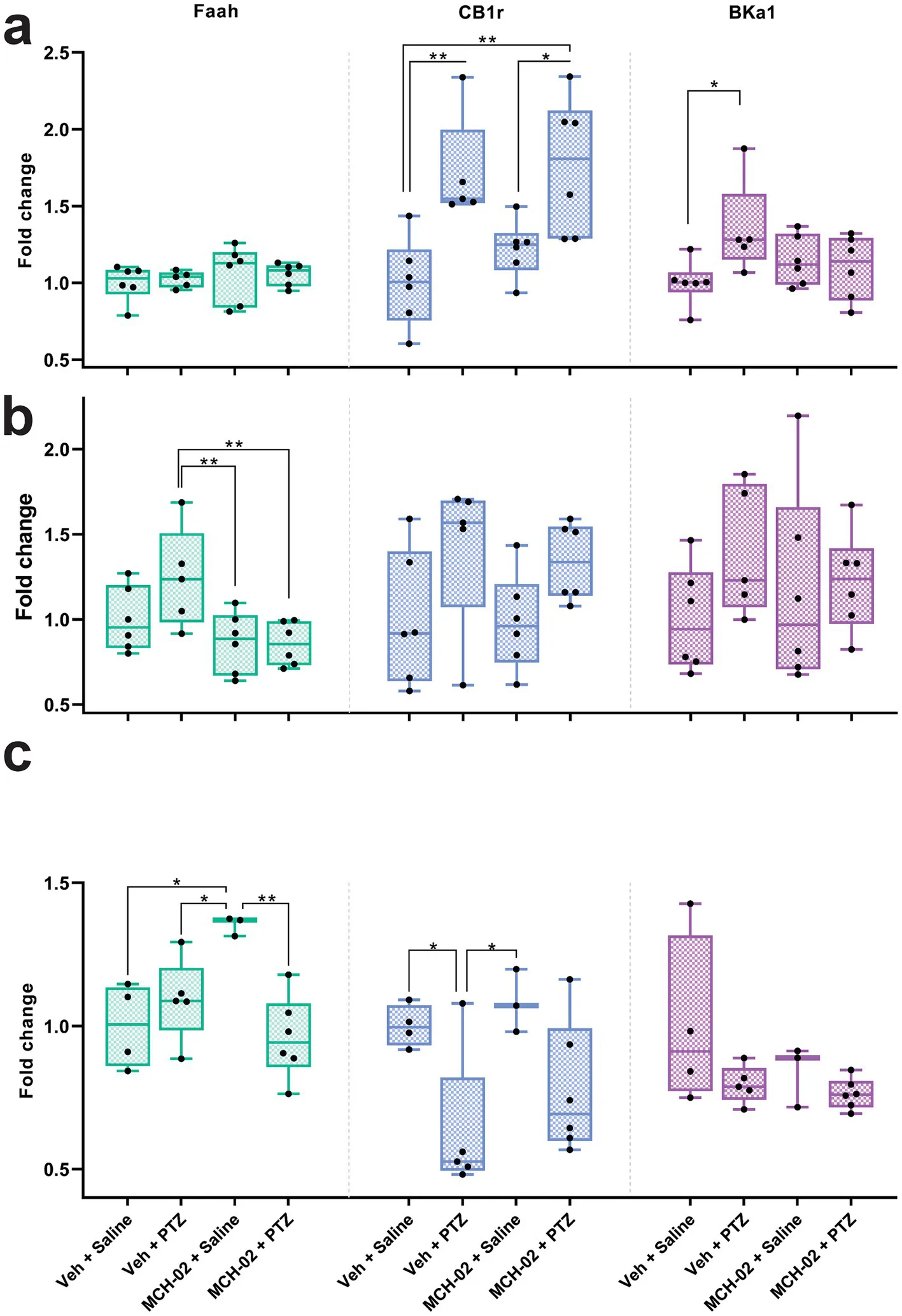
Relative hippocampal mRNA abundance following PTZ kindling and MCH-02 administration. Fold changes in Faah, Cnr1, Napepld, and Kcnma1 mRNA abundance were evaluated by RT-qPCR in **(a)** CA1, **(b)** CA3, and **(c)** dentate gyrus (DG). Schematics indicate the bilateral sampling regions. Boxes represent the interquartile range, horizontal lines indicate medians, and individual points represent biological replicates from individual animals. Group sizes were Veh + Saline, *n* = 6; Veh + PTZ, *n* = 5; MCH-02 + Saline, *n* = 6; and MCH-02 + PTZ, *n* = 6. Brackets identify pairwise comparisons based on estimated marginal means with Tukey-adjusted *p*-values: *p < 0.05 and **p < 0.01.

### 3.4 MCH-02 could not prevent protein expression changes produced by PTZ kindling

In the CA1 region of the hippocampus (Figure 5a), FAAH protein expression was not significantly affected by treatment group [Wald (3) = 1.2228, *p* = 0.7475]. In contrast, CB1 receptor expression showed a strong overall group effect [Wald (3) = 29.476, *p* = 1.778 × 10^−6^]. *Post hoc* comparisons showed higher CB1 receptor levels in both the Veh + PTZ (*p* = 0.0029) and MCH-02 + PTZ (*p* = 0.0012) groups than in Veh + Saline. In addition, CB1 receptor expression was higher in the MCH-02 + PTZ group than in the MCH-02 + Saline group (*p* = 0.0359). BKα1 expression was also significantly affected by group [Wald (3) = 9.2944, *p* = 0.02562], with higher levels observed in Veh + PTZ animals compared with Veh + Saline controls.

**Figure 5:**
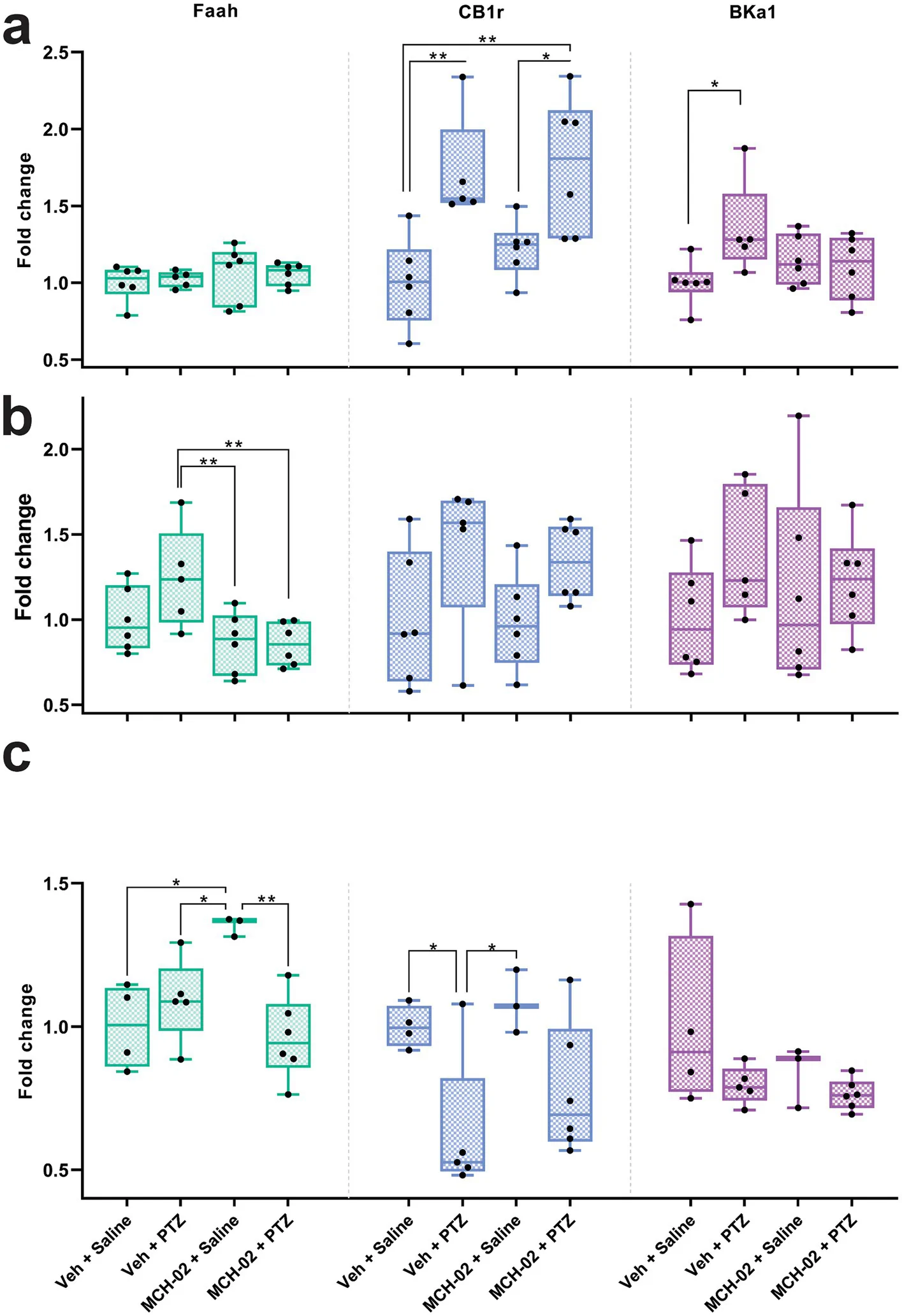
Relative hippocampal abundance of FAAH, CB1 receptor, and BK channel α subunit (BKα1) following PTZ kindling and MCH-02 administration. Protein abundance was evaluated by Western blot in **(a)** CA1, **(b)** CA3, and **(c)** dentate gyrus (DG). Boxes represent the interquartile range, horizontal lines indicate medians, and individual points represent biological replicates from individual animals. For CA1 and CA3, group sizes were Veh + Saline, *n* = 6; Veh + PTZ, *n* = 5; MCH-02 + Saline, *n* = 6; and MCH-02 + PTZ, *n* = 6. For DG, the corresponding group sizes were *n* = 4, 5, 3, and 6. Region-specific sample inclusion and exclusions are detailed in the Supplementary Information. Brackets identify pairwise comparisons based on estimated marginal means with Tukey-adjusted *p*-values: *p < 0.05 and **p < 0.01.

In CA3 (Figure 5b), FAAH was the only protein showing a significant overall group effect [Wald (3) = 14.6092, *p* = 0.002183]. *Post hoc* analysis indicated lower FAAH expression in both the MCH-02 + Saline and MCH-02 + PTZ groups compared with Veh + PTZ (*p* = 0.0048 for both comparisons). CB1 receptor expression also showed a significant overall group effect [Wald (3) = 8.5411, *p* = 0.03606]; however, no individual pairwise comparison remained statistically significant after multiple-comparison adjustment. BKα1 expression did not show a significant treatment-related change in this region.

Finally, in the dentate gyrus of the hippocampus (Figure 5c), significant group effects were detected for FAAH [Wald (3) = 26.596; *p* = 7.155e-06], CB1r [Wald (3) = 15.868; *p* = 0.001207], and BKa1 [Wald (3) = 8.4194; *p* = 0.03809]. *Post hoc* analysis showed that the MCH2 + Saline group had higher FAAH levels compared to Veh + Saline (*p* = 0.0110), Veh + PTZ (*p* = 0.0464), and MCH2 + PTZ (*p* = 0.0023). Conversely, CB1r expression was reduced in the Veh + PTZ group compared to both Veh + Saline (*p* = 0.0465) and MCH2 + Saline (*p* = 0.0213) groups. Interestingly, despite the significant group effect identified by the GLMM, *post hoc* analysis did not find any significant differences between groups in BKa1 expression.

Overall, these findings indicate that chronic MCH-02 administration did not prevent the region-specific protein remodeling associated with PTZ kindling. Instead, effects on FAAH, CB1 receptor, and BKα1 expression varied across hippocampal subregions, supporting differential molecular remodeling rather than uniform normalization or a protective effect.

## 4 Discussion

### 4.1 ECS remodeling induced by MCH-02 does not translate into protection against PTZ-kindling progression

The principal finding of the present study is the dissociation between hippocampal molecular remodeling and functional seizure outcomes following chronic administration of MCH-02. Although treatment induced significant alterations in the expression of endocannabinoid system (ECS)-related genes, including *FAAH*, *Cnr1*, and *Napepld*, as well as changes in CB1 receptor and BKα1 channel protein expression, these molecular adaptations did not prevent PTZ-kindling progression or reduce seizure susceptibility. Instead, MCH-02 transiently exacerbated seizure severity during the early phases of kindling acquisition.

This observation highlights an important conceptual distinction between biological target engagement and disease modification. While MCH-02 clearly induced molecular adaptations within ECS-associated pathways, these changes were insufficient to confer meaningful protection against the progressive development of seizure susceptibility. Therefore, the molecular alterations observed in the present study should be interpreted as evidence of hippocampal neurobiological remodeling rather than proof of therapeutic efficacy.

Indirect ECS activation through FAAH inhibition has attracted considerable interest as a potential anticonvulsant strategy. FAAH inhibitors such as URB597 increase endogenous anandamide levels and have been reported to suppress epileptiform activity while preserving synaptic plasticity and avoiding some adverse effects associated with direct CB1 agonists (15, 37). Similarly, phytocannabinoids such as cannabidiol have demonstrated efficacy in reducing seizure burden in both experimental and clinical settings through mechanisms involving ECS-related signaling pathways (38, 39). Collectively, these findings support the ECS as an important regulator of network excitability and a promising therapeutic target in drug-resistant epilepsy (1, 40).

Transcript-level differences were not consistently accompanied by corresponding differences in protein abundance. In CA1, higher Faah mRNA abundance in MCH-02 + PTZ than in Veh + Saline and Veh + PTZ was not accompanied by a significant overall group effect on FAAH protein abundance. Although CB1 receptor protein abundance was higher in both PTZ-treated groups than in Veh + Saline, the higher Cnr1 mRNA abundance in MCH-02 + PTZ relative to Veh + PTZ was not accompanied by a significant protein-level difference in that comparison.

This limited correspondence could reflect regulation at multiple stages of gene expression, including differences in translational efficiency and mRNA and protein turnover. However, these processes were not measured, and their contribution remains hypothetical. Differences in statistical significance between assays also do not, by themselves, establish biological uncoupling between transcript and protein responses. Accordingly, these findings do not establish coordinated changes in transcript and protein abundance, altered FAAH enzymatic activity, or enhanced CB1 receptor function.

### 4.2 State-dependent ECS signaling may underlie the early proconvulsant effects of MCH-02

One of the most unexpected findings of this study was the transient worsening of seizure severity observed during the initial stages of PTZ kindling. Although ECS activation is often considered anticonvulsant, increasing evidence indicates that cannabinoid signaling is highly state-dependent and can produce divergent effects depending on receptor availability, disease stage, and network context (7).

Consistent with previous reports, ECS remodeling during seizure development appears to follow a dynamic and biphasic pattern. Experimental epilepsy models have demonstrated early CB1 receptor internalization and downregulation following intense seizure activity, followed by compensatory receptor redistribution and upregulation during later stages (41, 42). Within this framework, the early proconvulsant effects observed after MCH-02 administration may reflect a mismatch between enhanced endocannabinoid tone and reduced functional CB1 receptor availability during the initial phases of network remodeling. Under these conditions, increased endocannabinoid signaling may fail to engage protective inhibitory mechanisms efficiently, thereby contributing to transient network instability.

Importantly, this interpretation remains hypothetical. The present study did not directly assess hippocampal endocannabinoid concentrations, FAAH enzymatic activity, CB1 receptor localization, receptor occupancy, or downstream signaling pathways. Consequently, the proposed relationship between ECS remodeling and seizure aggravation should be viewed as a mechanistic hypothesis requiring further experimental validation.

### 4.3 BK channel-associated remodeling represents a downstream correlate of ECS modulation

A novel aspect of the present study is the identification of BK channel-associated molecular remodeling following chronic administration of MCH-02. BK channels are critical regulators of membrane excitability and action potential repolarization, and alterations in BK channel function have been implicated in seizure susceptibility and epilepsy pathophysiology (17, 21, 43).

Previous studies have demonstrated that endocannabinoids, particularly anandamide, can modulate BK channel activity directly and indirectly, suggesting potential interactions between ECS signaling and intrinsic excitability mechanisms (16, 44). In the present study, MCH-02 administration was associated with increased *Kcnma1* expression and alterations in BKα1 protein levels, particularly within the CA1 region. These findings suggest that ECS-associated molecular remodeling may extend to ion channel systems involved in the regulation of excitability.

Nevertheless, the functional significance of these changes remains uncertain. No electrophysiological recordings were performed to evaluate BK currents, neuronal firing properties, intrinsic excitability, or synaptic function. Consequently, the observed alterations should be interpreted as evidence of BK channel-associated molecular plasticity rather than definitive proof of functional BK channel remodeling. Whether these changes represent adaptive, maladaptive, or compensatory responses remains unknown and will require future electrophysiological investigation.

### 4.4 Behavioral outcomes reinforce the dissociation between molecular adaptation and seizure protection

The behavioral findings further support the dissociation between molecular remodeling and functional seizure protection. A cohort-specific difference in novel-object discrimination was detected between MCH-02 + PTZ and Veh + PTZ animals; however, because this effect was observed only in cohort 1 and was not reproduced in cohort 2, it does not provide robust evidence for a general improvement in recognition memory. Consistently, the Barnes-maze probe test did not reveal treatment-related differences in target-quadrant occupancy. Thus, the behavioral data do not support a reproducible memory-enhancing effect of MCH-02 under the present experimental conditions.

Given the established role of the ECS in learning, memory, and synaptic plasticity (7, 45), it is plausible that some of the observed behavioral changes reflect selective modulation of recognition memory circuits. However, the absence of robust improvements across multiple behavioral domains and the lack of concomitant seizure protection suggest that these effects should be interpreted cautiously.

Rather than indicating disease modification, the behavioral data likely represent context-dependent functional correlates of ECS remodeling. Furthermore, the inherent variability of chronic PTZ-kindling models, combined with limited sample sizes in some experimental groups, warrants caution when interpreting these findings. Additional studies will be required to determine the reproducibility and biological significance of these behavioral observations.

### 4.5 Glial and inflammatory pathways as potential contributors to hippocampal remodeling

An additional mechanism that may help explain the dissociation between ECS remodeling and the absence of sustained protection against PTZ-kindling progression involves neuroinflammatory and glial pathways. Neuroinflammation is increasingly recognized as an important component of the progressive network remodeling associated with recurrent seizure activity, involving reciprocal interactions among neurons, microglia, astrocytes, cytokine signaling, and alterations in blood–brain barrier function. Because FAAH regulates the availability of anandamide and other N-acylethanolamines, its inhibition may influence these processes through mechanisms extending beyond neuronal CB1-mediated modulation of synaptic transmission.

Experimental evidence supports the capacity of FAAH inhibition to modulate neuroinflammatory responses under specific pathological conditions. Chronic administration of the selective FAAH inhibitor URB597 reduced hippocampal microglial activation and attenuated age-associated increases in IL-1β and TNF-α, effects accompanied by partial restoration of long-term potentiation in the dentate gyrus (46). Similarly, URB597 promoted an anti-inflammatory microglial phenotype in an Aβ-associated inflammatory model, reducing iNOS and pro-inflammatory cytokine expression while increasing Arg-1 and anti-inflammatory mediators (47). Together with the ability of URB597 to suppress hippocampal maximal dentate afterdischarges and prevent seizure-associated impairment of short- and long-term synaptic plasticity (15), these observations provide a biological basis for considering FAAH-dependent lipid signaling as a potential link among neuronal excitability, synaptic plasticity, and neuroinflammation.

However, FAAH inhibition should not be regarded as intrinsically or uniformly anti-inflammatory. Its biological consequences depend on cell type, pathological context, receptor availability, brain region, disease stage, and the relative contribution of cannabinoid and non-cannabinoid signaling pathways. This complexity is illustrated by the observation that FAAH-deficient astrocytes exposed to β-amyloid developed an exacerbated inflammatory phenotype characterized by increased production of pro-inflammatory cytokines and increased expression of COX-2, iNOS, and TNF-α. Importantly, these responses involved PPAR-α-, PPAR-γ-, and TRPV1-dependent mechanisms rather than CB1 or CB2 signaling, whereas exogenous anandamide, palmitoylethanolamide, and oleoylethanolamide produced anti-inflammatory effects (48). These findings indicate that altered FAAH-dependent lipid metabolism cannot be interpreted simply as an increase in cannabinoid-mediated anti-inflammatory tone and emphasize that the net biological effect of enhanced N-acylethanolamine availability is strongly dependent on the cellular and molecular environment.

This context dependence is particularly relevant to the present PTZ-kindling model. Repeated seizure activity engages multiple mutually reinforcing mechanisms including altered excitatory–inhibitory balance, ion-channel remodeling, synaptic plasticity, glial activation, and inflammatory signaling that collectively contribute to progressive seizure susceptibility. Consequently, even if MCH-02-mediated FAAH inhibition generated anti-inflammatory effects in specific cellular compartments, such effects may have been insufficient in magnitude, duration, or anatomical distribution to counteract the broader network adaptations driven by repeated PTZ exposure. Conversely, FAAH-dependent changes in lipid signaling could have produced different or even opposing effects across neuronal, microglial, and astrocytic populations. This provides a plausible framework for understanding why substantial ECS-associated molecular remodeling did not translate into sustained protection against kindling progression.

Nevertheless, this interpretation remains hypothetical because the present study did not directly quantify anandamide or related N-acylethanolamines, FAAH enzymatic activity, cytokines, microglial or astrocytic activation, or blood–brain barrier integrity. Accordingly, our findings do not demonstrate either the presence or absence of an anti-inflammatory action of MCH-02 in this model. Rather, they indicate that FAAH-associated ECS remodeling alone was insufficient to prevent progressive seizure susceptibility. Future studies combining direct measurements of FAAH target engagement and endocannabinoid lipidomics with markers of microglial and astrocytic activation, inflammatory mediators, and electrophysiological outcomes will be required to determine whether neuroinflammatory mechanisms contribute to the context-dependent effects of MCH-02 during PTZ kindling.

### 4.6 Statistical considerations and limitations in the interpretation of negative findings

The interpretation of the present findings should also consider the statistical and experimental limitations inherent to chronic PTZ-kindling studies. Group sizes were established *a priori* using statistical power calculations based on published effect estimates from behavioral paradigms comparable to those used in the present study. With a target power of 0.80 and a conservative significance threshold of α = 0.001, the Barnes maze yielded the more demanding estimate of at least seven animals per group, which was adopted for the experimental design. The complete derivation and analysis code are provided in the Supplementary material.

However, this power analysis was based primarily on behavioral endpoints and relatively large previously reported effect sizes and therefore should not be interpreted as demonstrating equivalent statistical sensitivity for every secondary, molecular, or longitudinal outcome examined in the study. This distinction is particularly relevant to PTZ kindling, which exhibits substantial inter-animal variability in seizure severity, latency, progression, and molecular responses. Consequently, the study may have had limited sensitivity to detect small or moderate treatment effects for some outcomes, and non-significant comparisons should not be interpreted as evidence of complete biological equivalence between groups.

To address repeated measurements and inter-individual variability, longitudinal outcomes were analyzed using mixed-effects approaches with animal identity incorporated as a random effect, while kindling progression was evaluated using an ordinal cumulative link mixed model. These approaches improve the use of repeated observations and account for within-animal dependency but do not eliminate limitations imposed by sample size or biological heterogeneity. Therefore, a type II error cannot be excluded for secondary outcomes showing high variability or modest effect sizes.

Importantly, the principal conclusion that MCH-02 did not provide sustained protection against PTZ-kindling progression is supported not by an isolated non-significant comparison but by the longitudinal behavioral pattern: seizure progression remained broadly comparable between the PTZ-treated groups throughout the protocol, while MCH-02 produced a significant transient increase in seizure severity during the initial phase. Thus, the available data do not support a robust disease-modifying effect under the present experimental conditions. Nevertheless, larger confirmatory studies powered specifically for longitudinal seizure outcomes and molecular endpoints will be necessary to determine whether smaller treatment effects exist and to establish their biological relevance.

Additional limitations should also be considered. The study did not directly determine brain concentrations of MCH-02, pharmacokinetic parameters, FAAH enzymatic inhibition *in vivo*, endocannabinoid concentrations, CB1 receptor occupancy or signaling, or functional BK-channel activity. Furthermore, molecular measurements represent endpoint adaptations following completion of the kindling protocol and therefore cannot establish the temporal sequence or causal relationships among FAAH modulation, ECS remodeling, BK-channel-associated changes, and seizure progression. These limitations restrict mechanistic interpretation and reinforce the need to distinguish molecular association from functional target engagement and disease modification.

### 4.7 Integrated interpretation and conclusion

Under the conditions examined, chronic administration of MCH-02 at 1 mg/kg did not demonstrate sustained protection against PTZ-induced seizure susceptibility. Seizure severity was greater in the MCH-02 + PTZ group during the first kindling trial, without significant between-group differences in subsequent trials. Survival did not differ significantly among groups. Evidence of cognitive benefit was also inconsistent: the increase in NOR discrimination index was restricted to cohort 1, and Barnes maze probe-test outcomes did not support improved spatial memory retention. Together, these findings do not establish a reproducible anticonvulsant, survival, or memory-enhancing benefit of this regimen.

The molecular analyses identified region- and marker-specific differences in hippocampal ECS-related transcripts and CB1 receptor and BKα1 protein abundance. However, these findings did not constitute a uniform response to MCH-02. Significant overall group effects were not always accompanied by significant adjusted pairwise comparisons, and transcript-level differences were not consistently reflected in protein abundance. In CA1, increased Faah mRNA was not accompanied by a significant group effect on FAAH protein abundance. Similarly, the higher Cnr1 mRNA level in MCH-02 + PTZ relative to Veh + PTZ was not accompanied by a significant CB1 protein difference between those groups. Consequently, these observations do not establish coordinated pathway activation or a causal relationship between molecular changes and seizure outcomes.

Although MCH-02 inhibited recombinant FAAH *in vitro*, its systemic and brain exposure and its effects on brain FAAH activity were not determined. The present study therefore cannot attribute the behavioral or molecular findings to demonstrated FAAH target engagement, altered endocannabinoid concentrations, or changes in CB1 receptor or BK-channel function. Qualitative immunofluorescence observations do not establish increased neuroinflammation or treatment-dependent glial activation. Likewise, the representative EEG recording from the separate pilot study does not establish recurrent spontaneous seizures or chronic seizure burden in the main experimental cohorts.

Overall, the findings show that molecular differences can occur without a consistent protective effect on the measured behavioral outcomes. They do not demonstrate antiepileptogenic efficacy, but nonsignificant results should not be interpreted as equivalence or as excluding smaller effects, particularly given the limited sample sizes and absence of endpoint-specific prospective power calculations for seizure and molecular outcomes. Further studies combining plasma and brain pharmacokinetics, direct FAAH activity measurements, endocannabinoid profiling, functional electrophysiology, quantitative glial assessments, and systematic longitudinal EEG monitoring are needed to test the proposed mechanisms and establish whether alternative MCH-02 regimens offer reproducible benefit.

## Data Availability

The raw data supporting the conclusions of this article will be made available by the authors, without undue reservation.
