# Supplementary material for: MCH-02, a synthetic macamide analog with in vitro FAAH-inhibitory activity, modulates hippocampal endocannabinoid system and BK channel expression without preventing PTZ-induced progressive seizure susceptibility in rats
Source: Front Neurol. 2026 Oct 16;17:1843264. doi: 10.3389/fneur.2026.1843264 (PMC13719350; doi:10.3389/fneur.2026.1843264)
Supplement: Supplementary file 1 [file Data_Sheet_1.PDF]

SUPPLEMENTARY INFORMATION

SECTION S1. *In vitro FAAH inhibition by synthetic macamides*

To evaluate the ability of synthetic macamides to modulate fatty acid amide hydrolase (FAAH) activity, an in vitro fluorometric FAAH inhibitor-screening assay was conducted with recombinant human FAAH. Three compounds were tested at concentrations of 1–100 μM: MCH-01 (N-benzyl-(9Z,12Z)-octadecadienamide), MCH-02 N-(8Z-heptadecen-1-yl)-O-(3-pyridylmethyl) carbamate and MCH-03 ((5Z,8Z,11Z,14Z)-2-hydroxy-N-(2-hydroxyethyl)-eicosa-5,8,11,14-tetraenamide).

FAAH activity was quantified by monitoring the generation of the fluorescent product 7-amino-4-methylcoumarin (AMC) following enzymatic hydrolysis of the fluorogenic substrate. Positive controls (URB597 and JZL195) produced the expected reduction in fluorescence, confirming assay sensitivity and validity. Among the compounds evaluated, MCH-02 exhibited a clear inhibitory effect on FAAH activity, evidenced by a concentration-dependent reduction in AMC production compared with vehicle-treated controls. This effect was observed throughout the incubation period and was most pronounced at higher concentrations, indicating effective interaction with the FAAH catalytic machinery.

In contrast, MCH-01 and MCH-03 did not inhibit FAAH activity under the experimental conditions tested. Unexpectedly, both compounds produced higher fluorescence signals than vehicle controls, suggesting increased substrate hydrolysis and an apparent enhancement of FAAH activity. Although the mechanism underlying this effect remains unclear, these findings indicate that neither MCH-01 nor MCH-03 behaves as a FAAH inhibitor in this assay.

Based on these results, we selected MCH-02 as the lead compound for subsequent in vivo studies. Its inhibitory activity on FAAH supports the hypothesis that this macamide may enhance endocannabinoid signaling by reducing anandamide degradation. Nevertheless, because these experiments used recombinant human FAAH in a cell-free system, additional studies are needed to determine MCH-02's potency, selectivity, pharmacokinetic properties, and in vivo target engagement. Overall, the screening assay identified MCH-02 as the only candidate displaying concentration-dependent FAAH inhibitory activity under the in vitro assay conditions, providing the pharmacological rationale for its selection in subsequent investigations of endocannabinoid modulation during PTZ-induced epileptogenesis

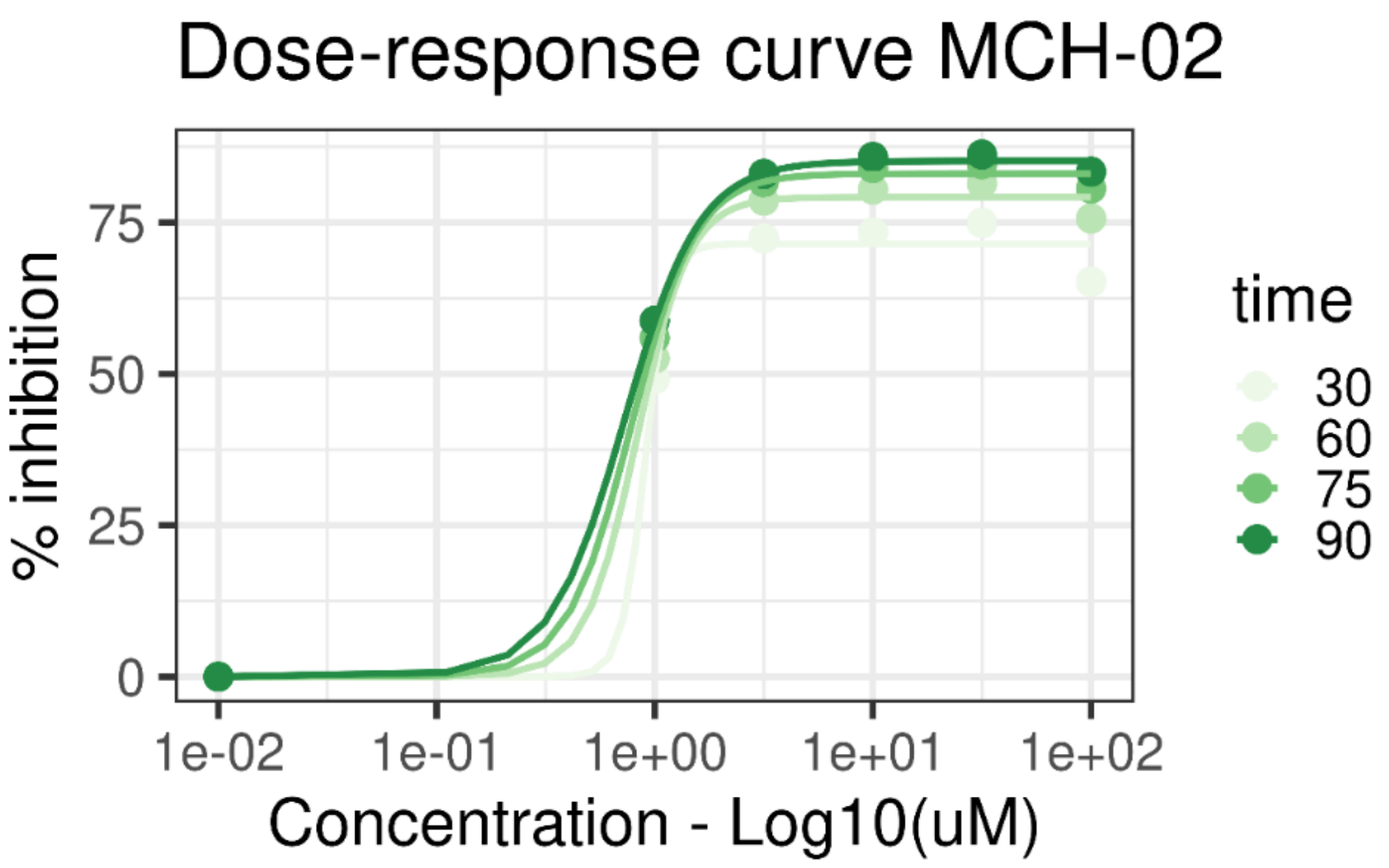

Figure S1. Four-parameter log-logistic regression fitted to the dose–response curve of MCH-02, showing the relationship between compound concentration and percentage inhibition of FAAH activity.

SECTION S2. *Long-term EEG assessment following PTZ kindling*

S2.1. Exploratory post-kindling EEG monitoring.

Exploratory EEG monitoring was performed in a subset of animals (2, 3 and 4 animals per experimental control, PTZ and MCH2 groups respectively) during the 2–3-month period following completion of the PTZ-kindling protocol. Animals underwent extended continuous EEG recording sessions (>24 h) to determine whether spontaneous electrographic seizure-like events could be detected during the post-kindling period. This supplementary assessment was exploratory and was not prospectively designed or powered to compare spontaneous seizure frequency, duration, cumulative seizure burden, or treatment effects among experimental groups. Accordingly, the EEG recordings were used as qualitative supporting evidence of spontaneous electrographic activity after PTZ kindling rather than as a quantitative measure of chronic epilepsy.

S2.2. Representative spontaneous electrographic seizure-like activity

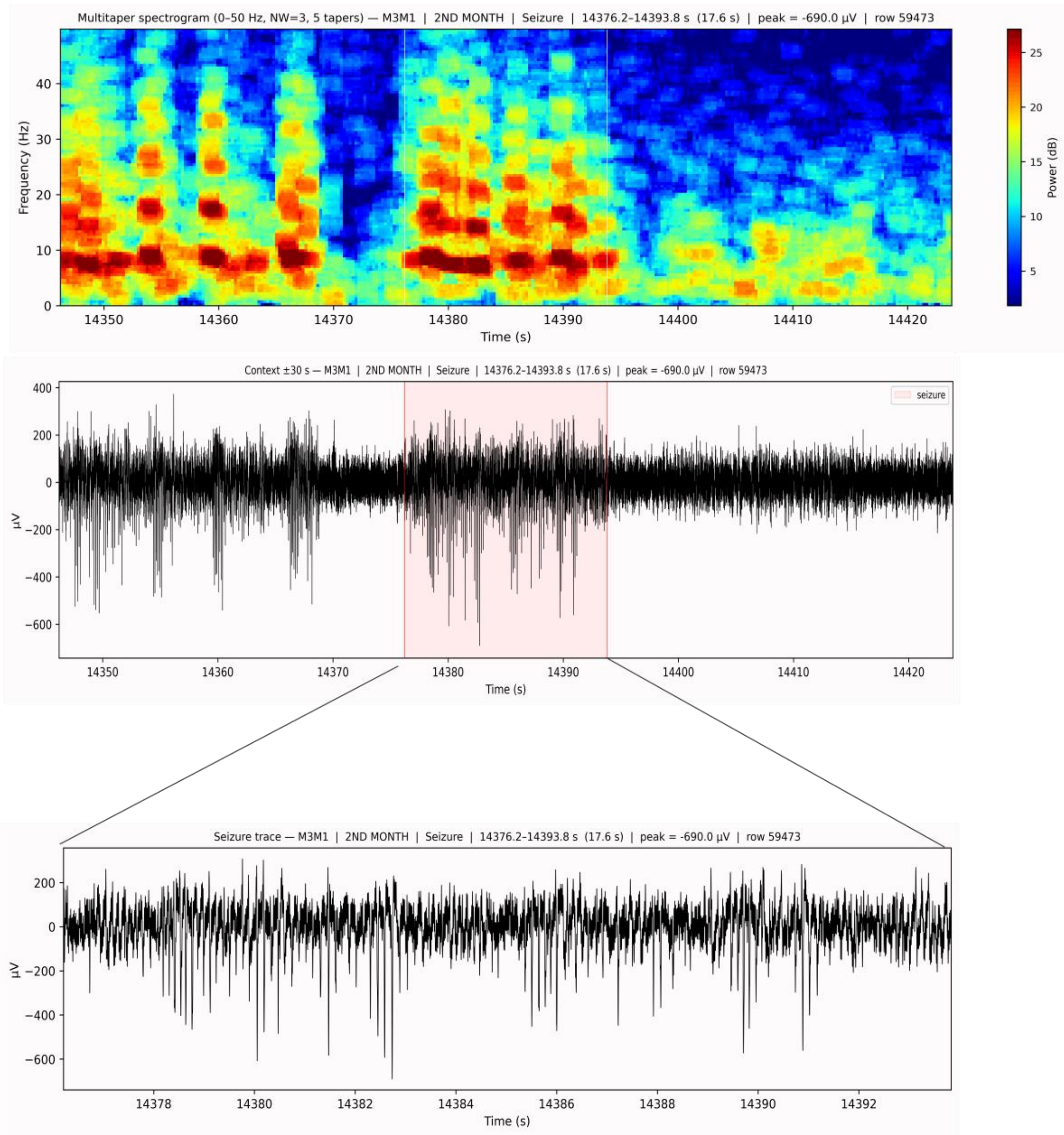

Figure S2. Representative spontaneous electrographic seizure-like activity detected during post-kindling EEG monitoring. Representative EEG recording obtained during exploratory monitoring following completion of the PTZ-kindling protocol. The upper panel shows the multilayer spectrogram (0–50 Hz), the middle panel shows the corresponding 30-s EEG segment with the identified electrographic event highlighted, and the lower panel shows an expanded view of the event. The representative event lasted approximately 17.6 s and reached a peak amplitude of −690 µV. This trace is presented

as qualitative evidence that spontaneous electrographic seizure-like activity could be detected during post-kindling monitoring and was not used to establish chronic epilepsy or to quantitatively compare spontaneous seizure burden among experimental groups.

SECTION S3. *Molecular sample quality and validation*

S3.1. RNA quantification and quality control.

Total RNA obtained from hippocampal microdissections (CA1, CA3, and dentate gyrus) was quantified by spectrophotometry prior to RT-qPCR analyses. RNA concentrations ranged from 91.9 to 311.5 ng/µL across all samples, reflecting differences in tissue yield associated with the size and number of microdissections collected from each hippocampal subregion. The highest RNA concentrations were observed in dentate gyrus samples, whereas the lowest concentrations were detected in a subset of CA3 microdissections. RNA purity was evaluated using the A260/A280 absorbance ratio. Across all experimental groups and hippocampal regions, A260/A280 values ranged from 1.68 to 1.95, indicating acceptable RNA purity and minimal protein contamination. Most samples exhibited ratios between 1.80 and 1.95, consistent with RNA of suitable quality for downstream gene expression analyses. To ensure standardized template input for reverse transcription and quantitative PCR, RNA samples were diluted to a working concentration of 25 ng/µL prior to cDNA synthesis.

S3.2. RNA Integrity Assessment.

RNA integrity was evaluated by electrophoresis on 1% agarose gels. All samples displayed well-defined 28S and 18S ribosomal RNA bands, with visible 5.8S RNA, indicating preservation of RNA integrity throughout tissue processing, extraction, and procedures (Supplementary **Figure S1).** No evidence of substantial RNA degradation or fragmentation was observed.

Table of primers:

Gene symbol	Amplicon length		Sequence (5'->3')	Oligo length	BLAST - value
Faah	116	**For:**	GTTCACCTTGGACCCTACCG	20	0.002
		**Rev:**	CTGGGCTGGGCATGGTATAG	20	0.002
Napepld	115	**For:**	CCTTTCTGGGGCTGTTTGGA	20	0.002
		**Rev:**	TGGATACTGATGGCTTGGCG	20	0.002
Kcnma1	101	**For:**	CAGTGGGACGCACATTCTAA	20	0.002
		**Rev:**	GGTACAGCTCTGTGTCAGGG	20	0.002
Cnr1	115	**For:**	GCCTTCCTACCACTTCATCG	20	0.002
		**Rev:**	ACATTGGGGCTGTCTTTACG	20	0.002
Hprt1	115	**For:**	GGACCTCTCGAAGTGTTGGA	21	0.002
		**Rev:**	CTTTTCCACTTTCGCTGATGA	21	0.002
Gapdh	116	**For:**	CATCTTCCAGGAGCGAGATC	20	0.002
		**Rev:**	GGAGATGATGACCCTTTTGG	20	0.002

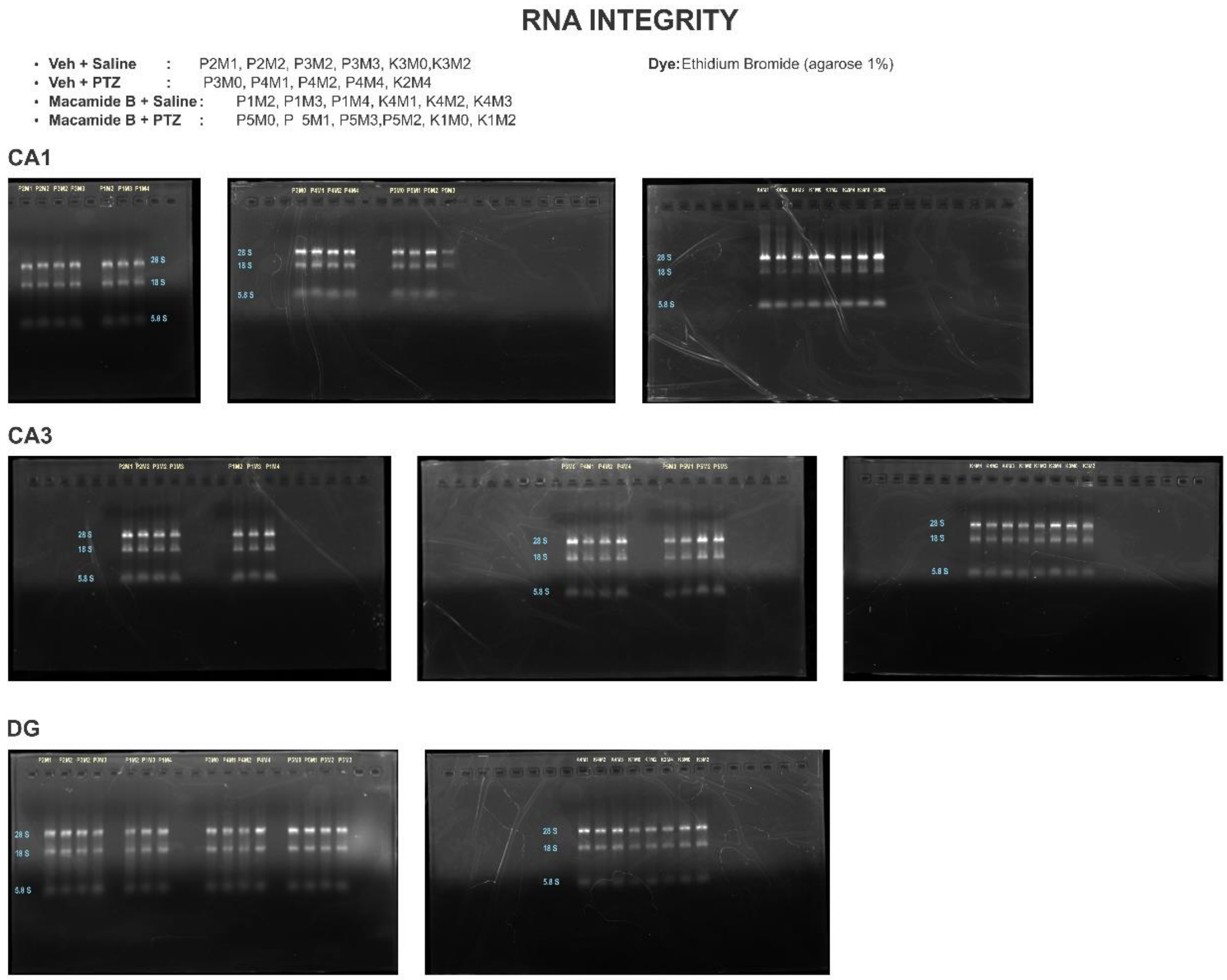

Figure S3. Collectively, these results demonstrate that the RNA extraction and purification workflow yielded RNA samples of sufficient concentration, purity, and integrity for reliable RT-qPCR analysis.

S3.3 Determination of primer amplification efficiency

We determined the amplification efficiency of the primer pairs used for gene expression analysis by generating standard curves from serial dilutions of cDNA. For this purpose, cDNA from representative study samples was used to provide a homogeneous template for evaluating each primer pair. We prepared five cDNA concentrations (50, 5, 0.5, 0.05, and 0.005 ng) using serial 1:10 dilutions. We analyzed each concentration in technical triplicates. RT-qPCR reactions were prepared using the same primer concentrations, final reaction volume, master mix, and amplification conditions subsequently employed for gene expression analysis. We included a no-template control (NTC) for each primer pair, in which cDNA was replaced with nuclease-free water. We recorded the quantification cycle (Cq) values for each technical replicate and calculated the mean Cq for each cDNA concentration. Subsequently, the base-10 logarithm of the cDNA concentration was calculated, and a standard curve was generated by linear regression analysis, with Cq as the dependent variable and the $\log_{10}$ of the initial cDNA concentration as the independent variable. The standard curve was expressed according to the following equation:

$$\boldsymbol{Cq} = m(log10[cDNA]) + b$$

- Where *m* represents the slope of the standard curve and *b* represents the y-intercept.
- The amplification efficiency (E) of each primer pair was calculated from the slope of the standard curve using the following equation:

$$\boldsymbol{E}(\%) = \left[10^{\left\{-\frac{1}{m}\right\}} - 1\right] x100$$

The quality of the standard curve was assessed using the coefficient of determination (R^2), with values close to 1 indicating a high degree of linearity between the initial template concentration and the corresponding Cq values. The specificity of the amplification products was evaluated by melting-curve analysis immediately after the amplification step. The presence of a single, well-defined melting peak was considered indicative of predominant amplification of a specific product. The presence of additional peaks or melting profiles consistent with nonspecific products or primer-dimer formation was considered indicative of potential nonspecific amplification. Primer pairs showing an amplification efficiency within the range of 90–115%, adequate linearity of the standard curve, and a melting profile consistent with amplification of a specific product were considered suitable for subsequent gene expression analysis by RT-qPCR.

Gapdh

Con (ng)	*log(ng)*	*Cq*
**50**	1.698970004	15.804
**50**	1.698970004	15.902
**50**	1.698970004	15.659
**5**	0.698970004	18.642
**5**	0.698970004	18.84
**5**	0.698970004	18.552
**0.5**	-0.301029996	21.876
**0.5**	-0.301029996	21.867
**0.5**	-0.301029996	21.81

**m**	-3.03133333
**b**	20.8912555
**Amplification factor**	2.13741001
**Efficiency**	113.741001

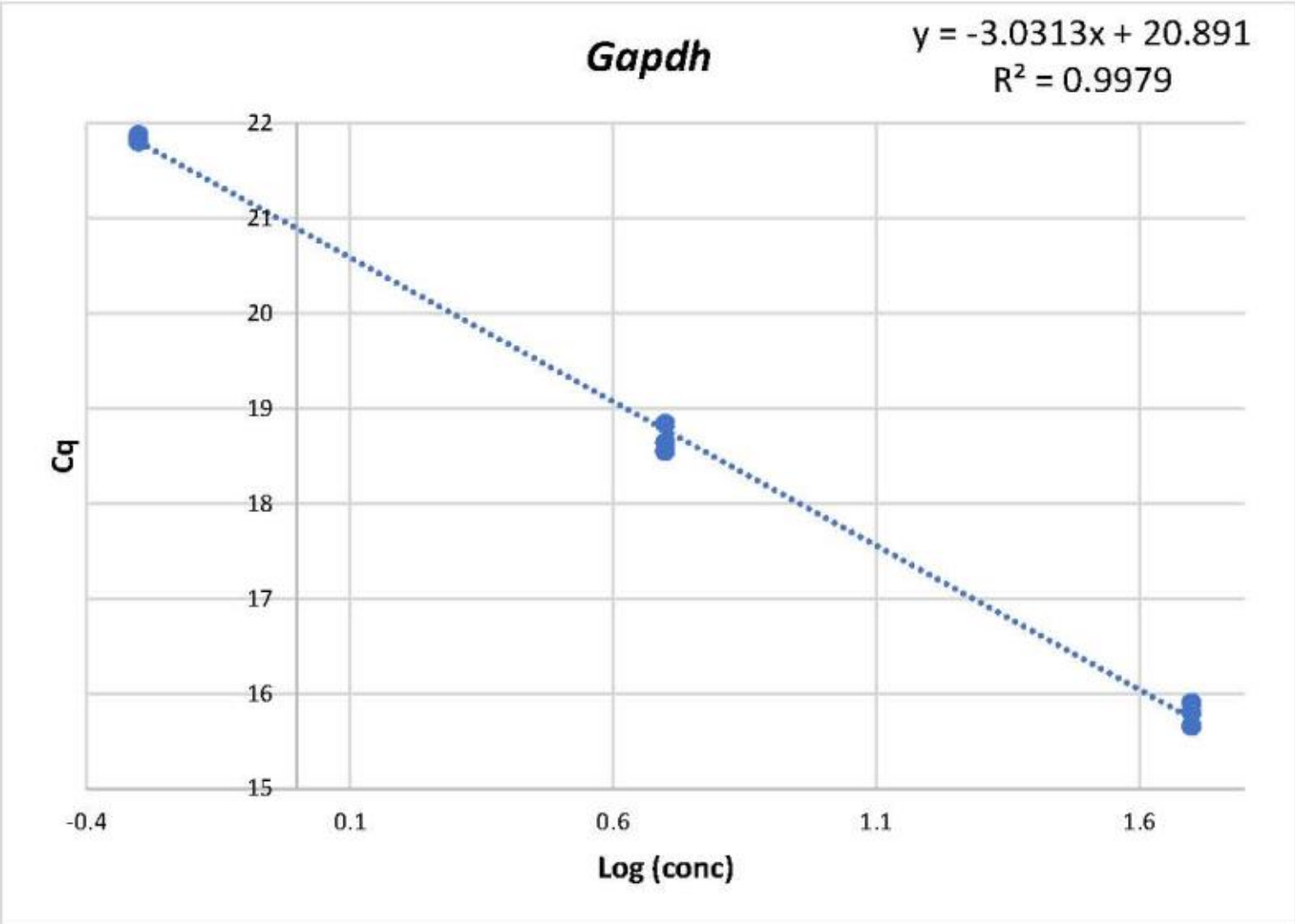

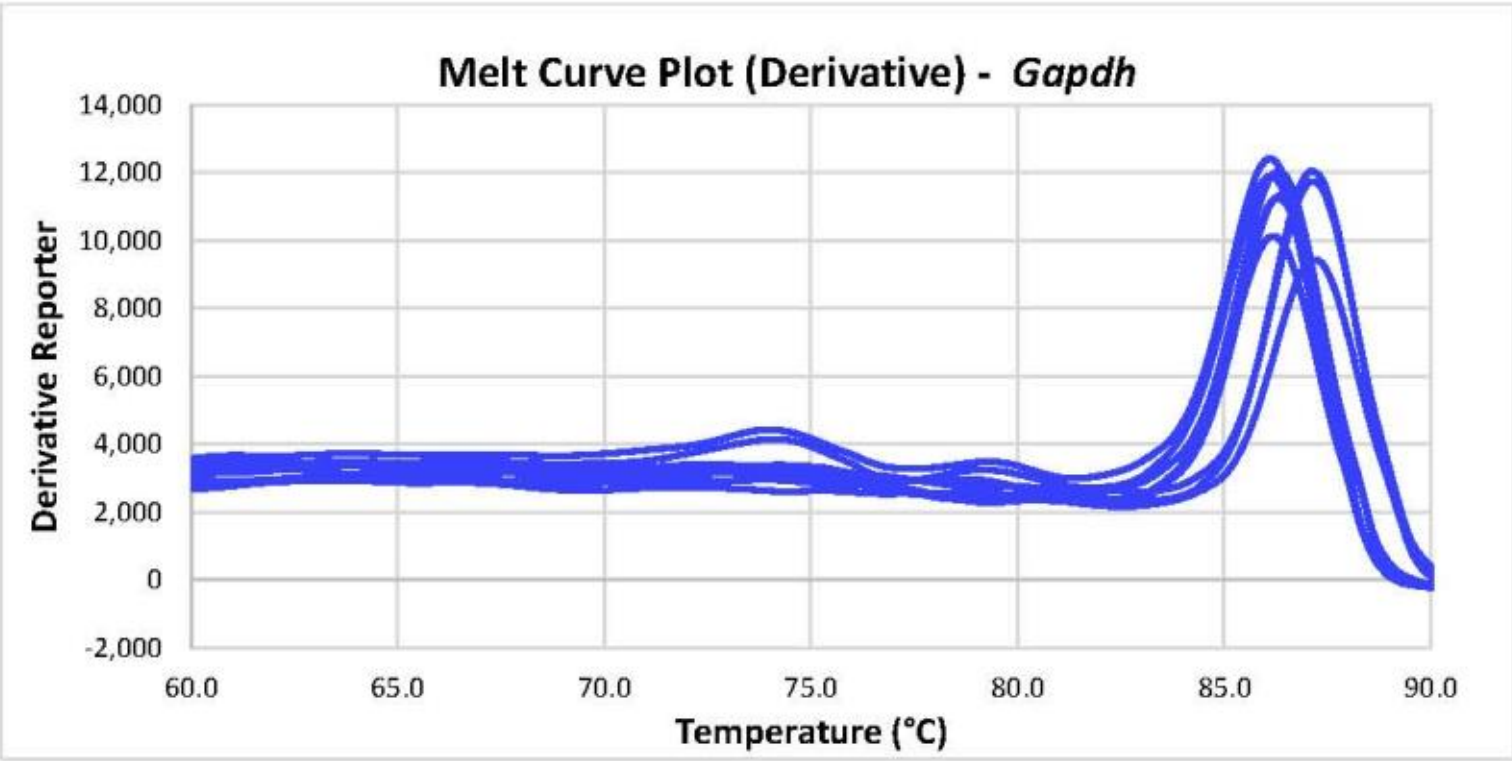

Hprt1

Con (ng)	*log(ng)*	*Cq*
**50**	1.698970004	20.015
**50**	1.698970004	20.251
**50**	1.698970004	20.096
**0.5**	-0.301029996	26.206
**0.5**	-0.301029996	26.101
**0.5**	-0.301029996	26.356
**0.05**	-1.301029996	29.815
**0.05**	-1.301029996	29.849
**0.05**	-1.301029996	29.734

m	-3.2011
b	25.484
**Amplification factor**	2.05301733
**Efficiency**	105.301733

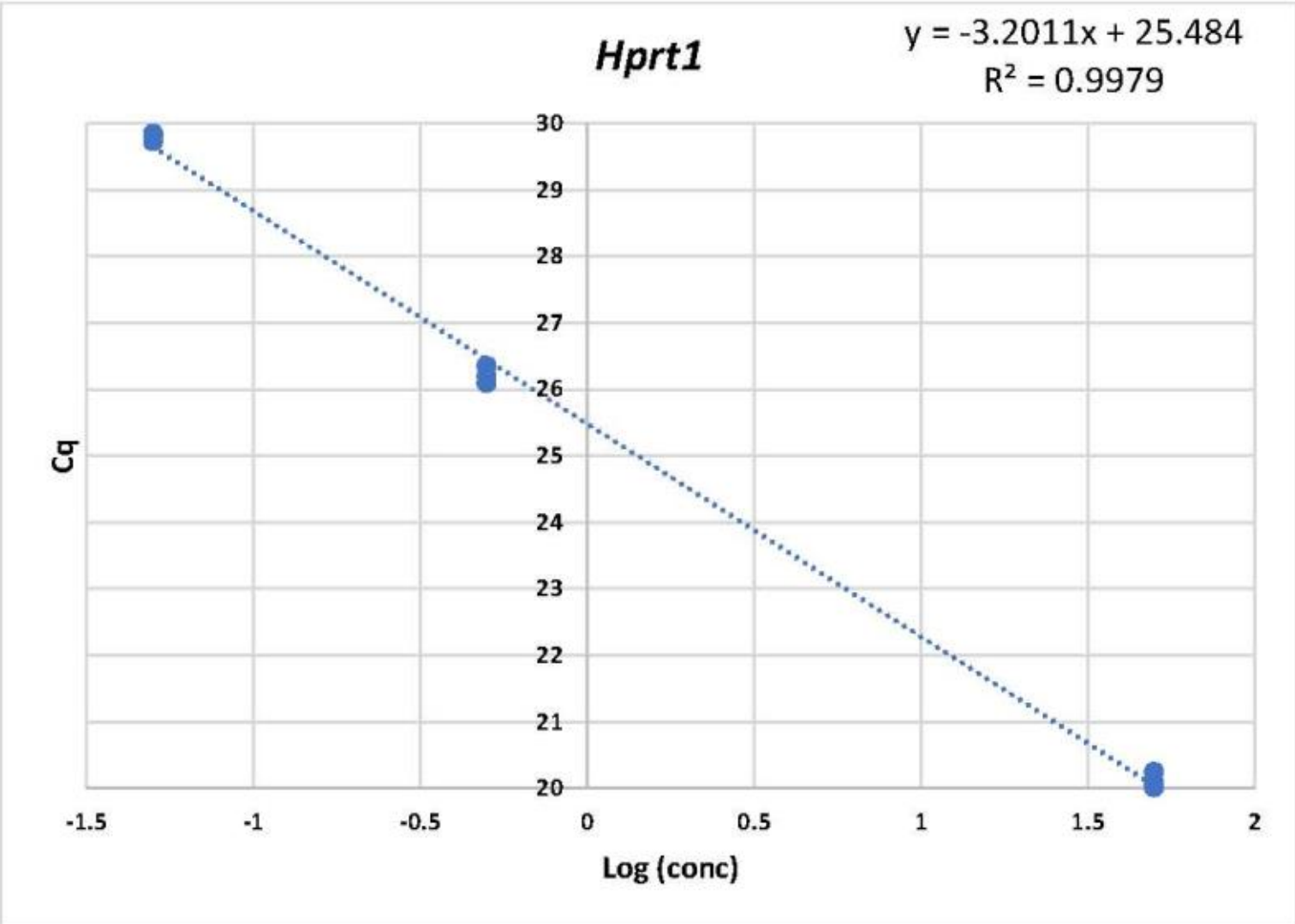

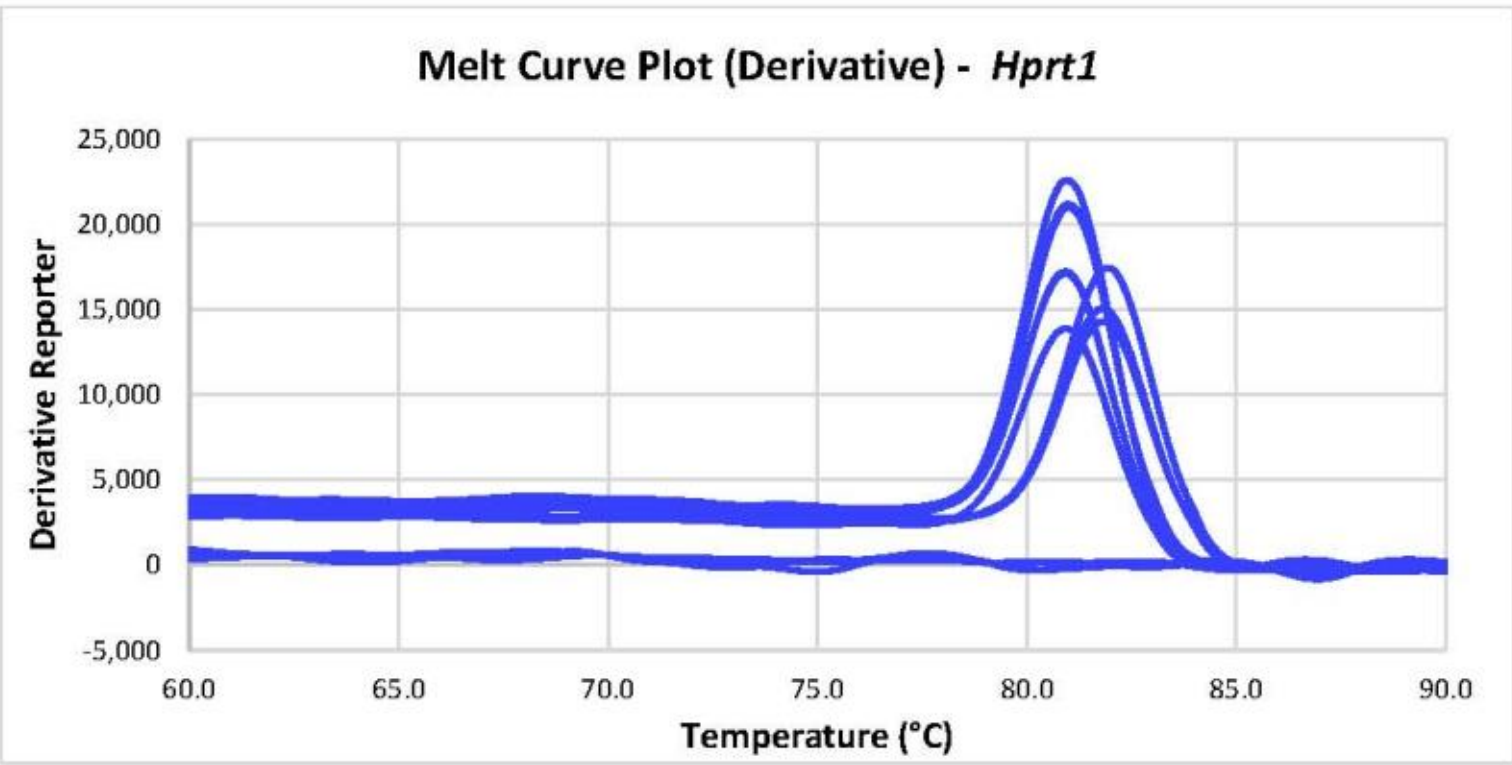

Faah

Con (ng)	log(ng)	Cq
**50**	1.698970004	20.002
**50**	1.698970004	20.179
**50**	1.698970004	20.313
**5**	0.698970004	23.116
**5**	0.698970004	23.189
**5**	0.698970004	23.223
**0.5**	-0.301029996	26.622
**0.5**	-0.301029996	26.466
**0.5**	-0.301029996	26.536
**0.05**	-1.301029996	30.134
**0.05**	-1.301029996	29.934
**0.05**	-1.301029996	30.275
**0.005**	-2.301029996	34.002
**0.005**	-2.301029996	34.112

**m**	-3.44715556
**b**	25.747825
**Amplification factor**	1.95026779
**Efficiency**	95.0267791

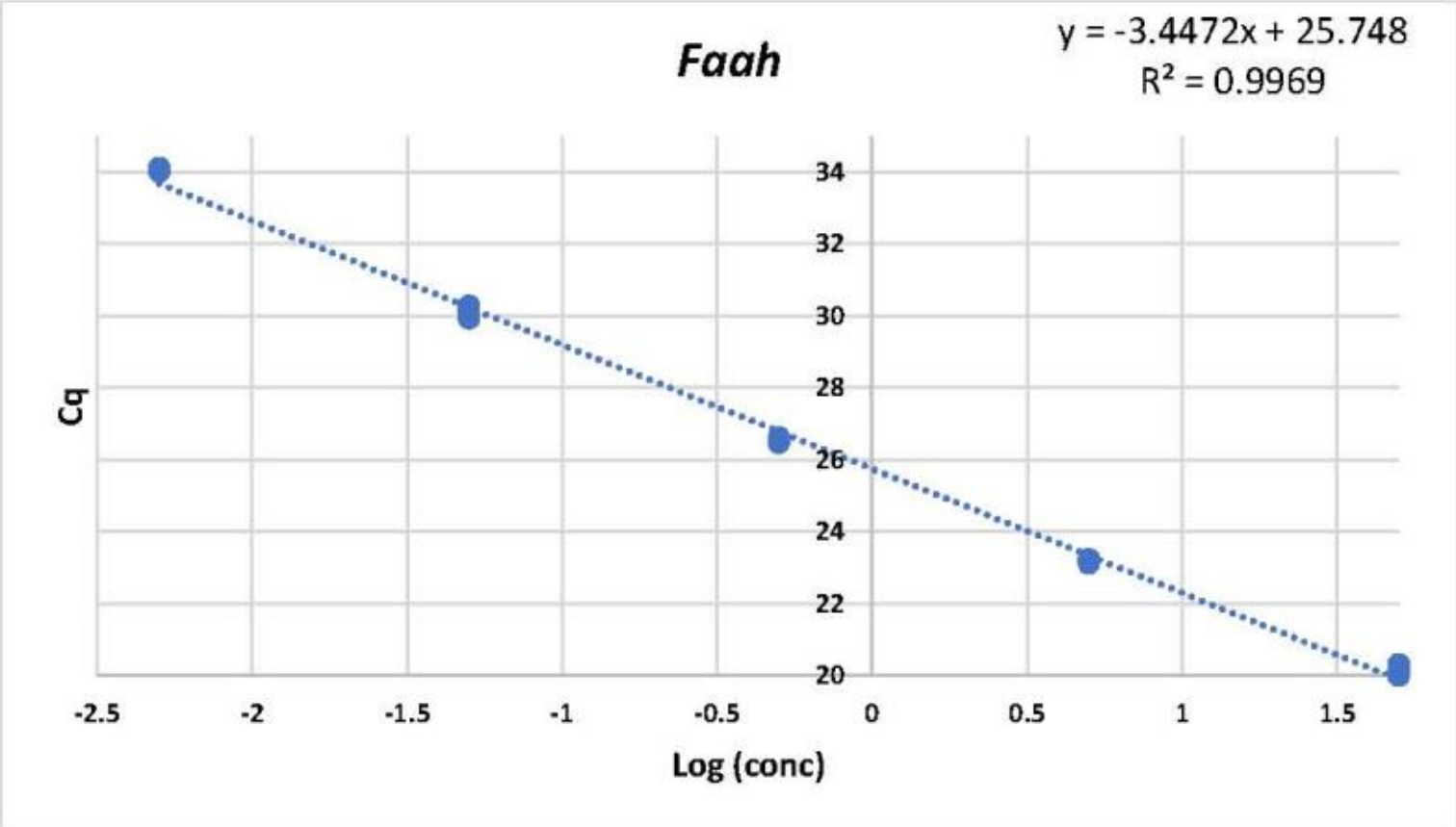

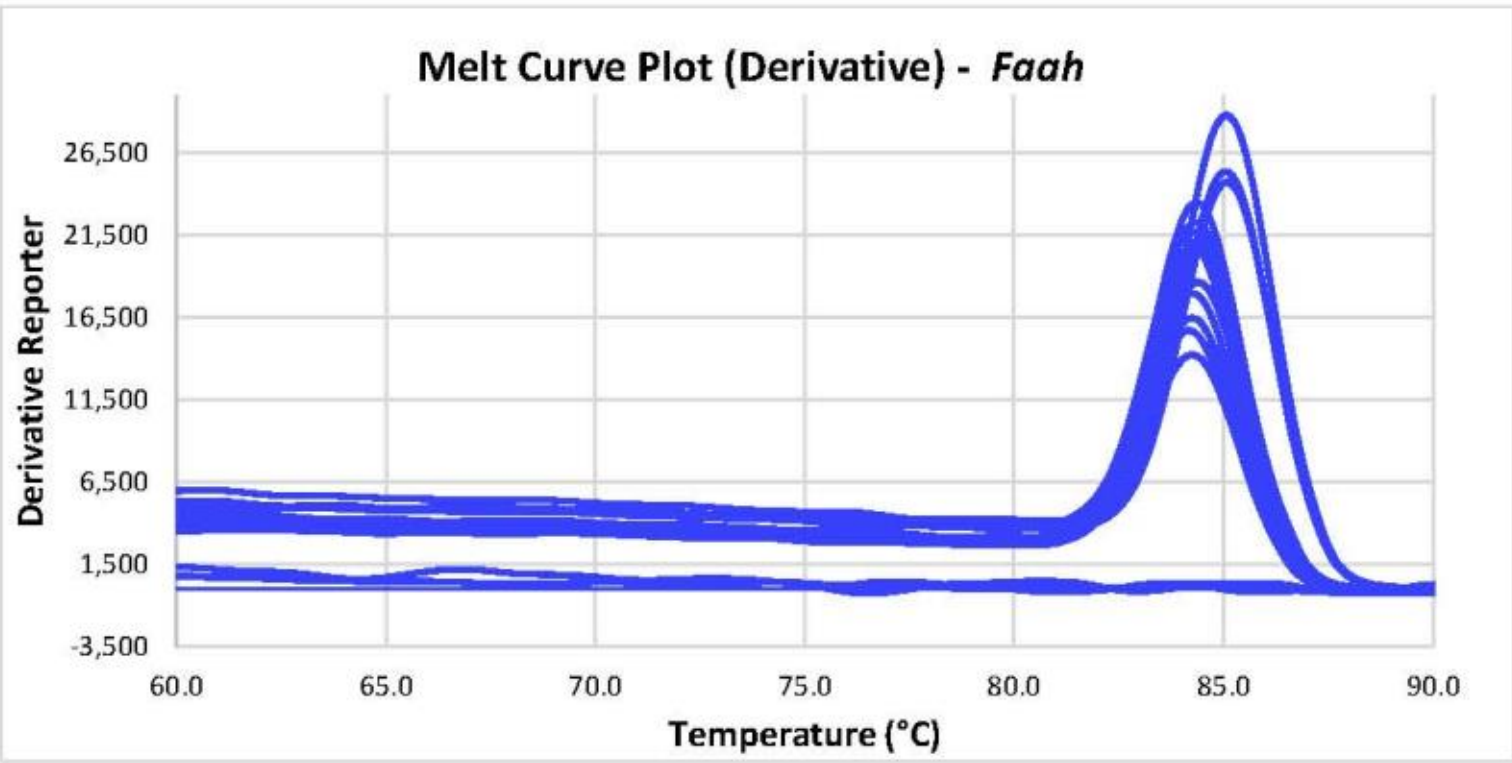

Cnr1

Con (ng)	*log(ng)*	*Cq*
**50**	1.698970004	18.266
**50**	1.698970004	18.284
**50**	1.698970004	18.19
**5**	0.698970004	21.236
**5**	0.698970004	21.274
**5**	0.698970004	21.246
**0.5**	-0.301029996	24.916
**0.5**	-0.301029996	24.66
**0.5**	-0.301029996	24.8
**0.05**	-1.301029996	28.074
**0.05**	-1.301029996	28.176
**0.05**	-1.301029996	28.37
**0.005**	-2.301029996	31.965

**m**	-3.39596296
**b**	23.8270096
**Amplification factor**	1.97000481
**Efficiency**	97.0004811

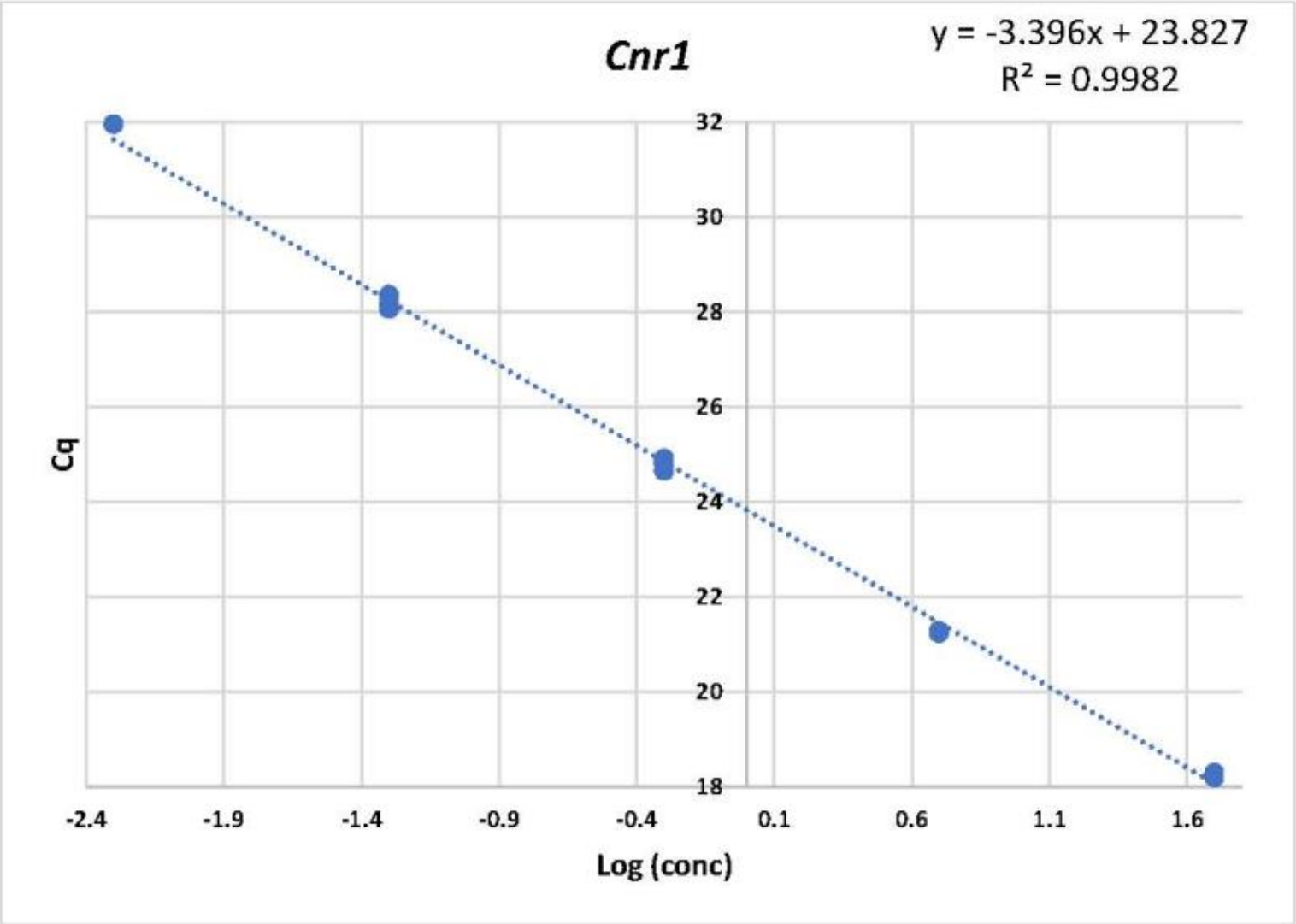

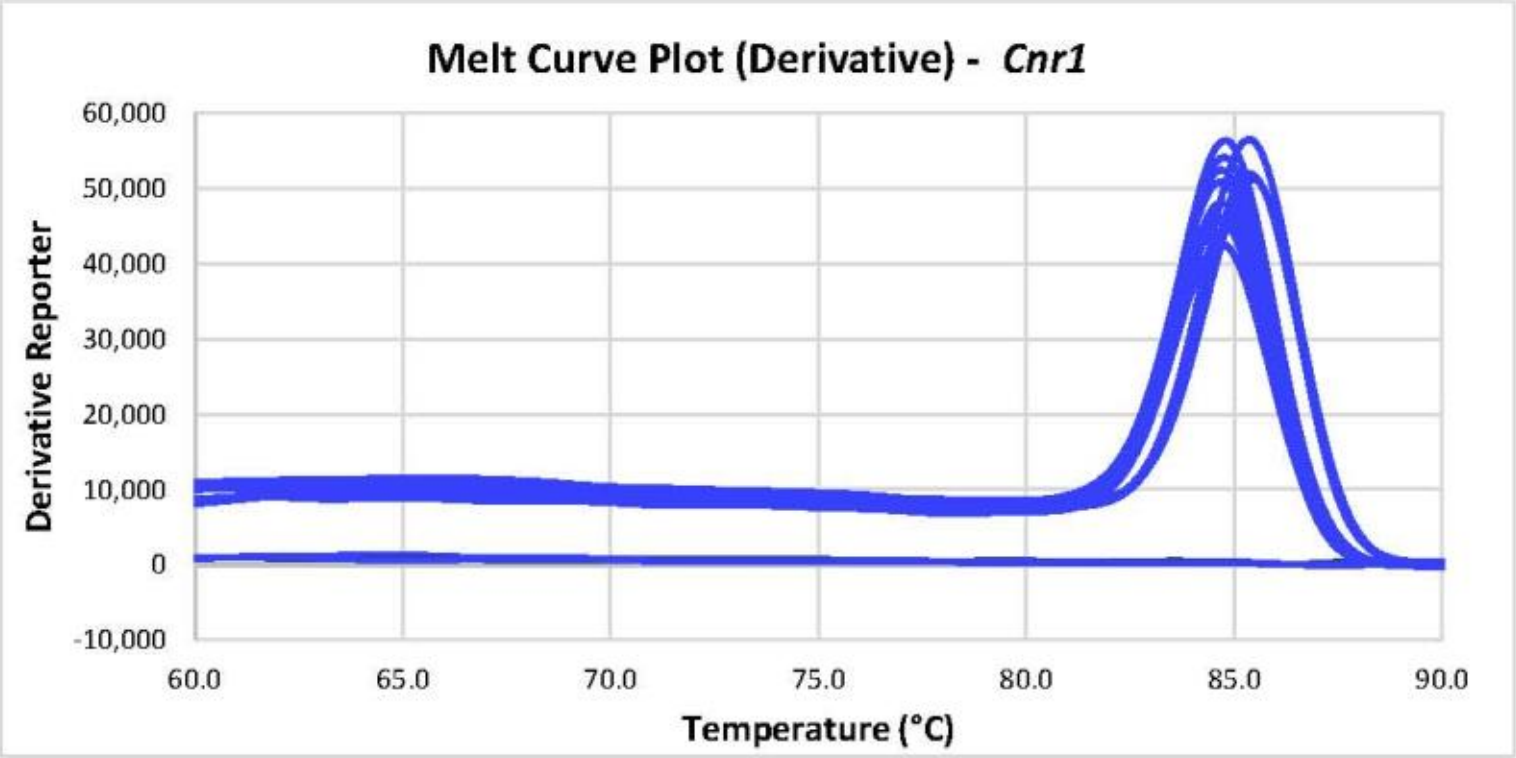

Napepld

Con (ng)	log(ng)	Cq
**50**	1.698970004	22.348
**50**	1.698970004	22.371
**50**	1.698970004	22.446
**5**	0.698970004	25.341
**5**	0.698970004	25.311
**5**	0.698970004	25.476
**0.5**	-0.301029996	28.887
**0.5**	-0.301029996	28.805
**0.5**	-0.301029996	28.582
**0.05**	-1.301029996	31.63
**0.005**	-2.301029996	34.324
**0.005**	-2.301029996	34.454

**m**	-3.03175
**b**	27.5958523
**Amplification factor**	2.13718688
**Efficiency**	113.718688

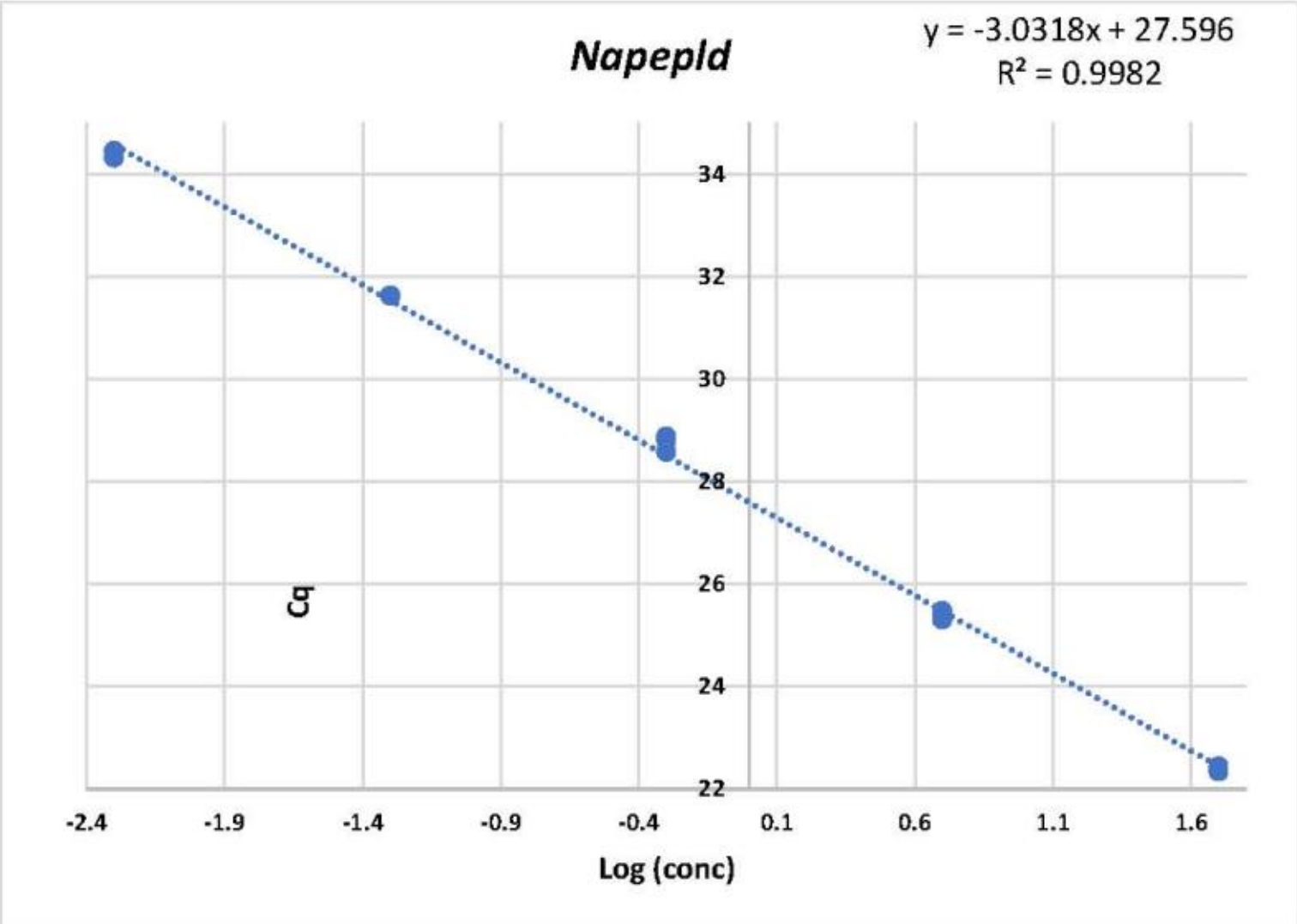

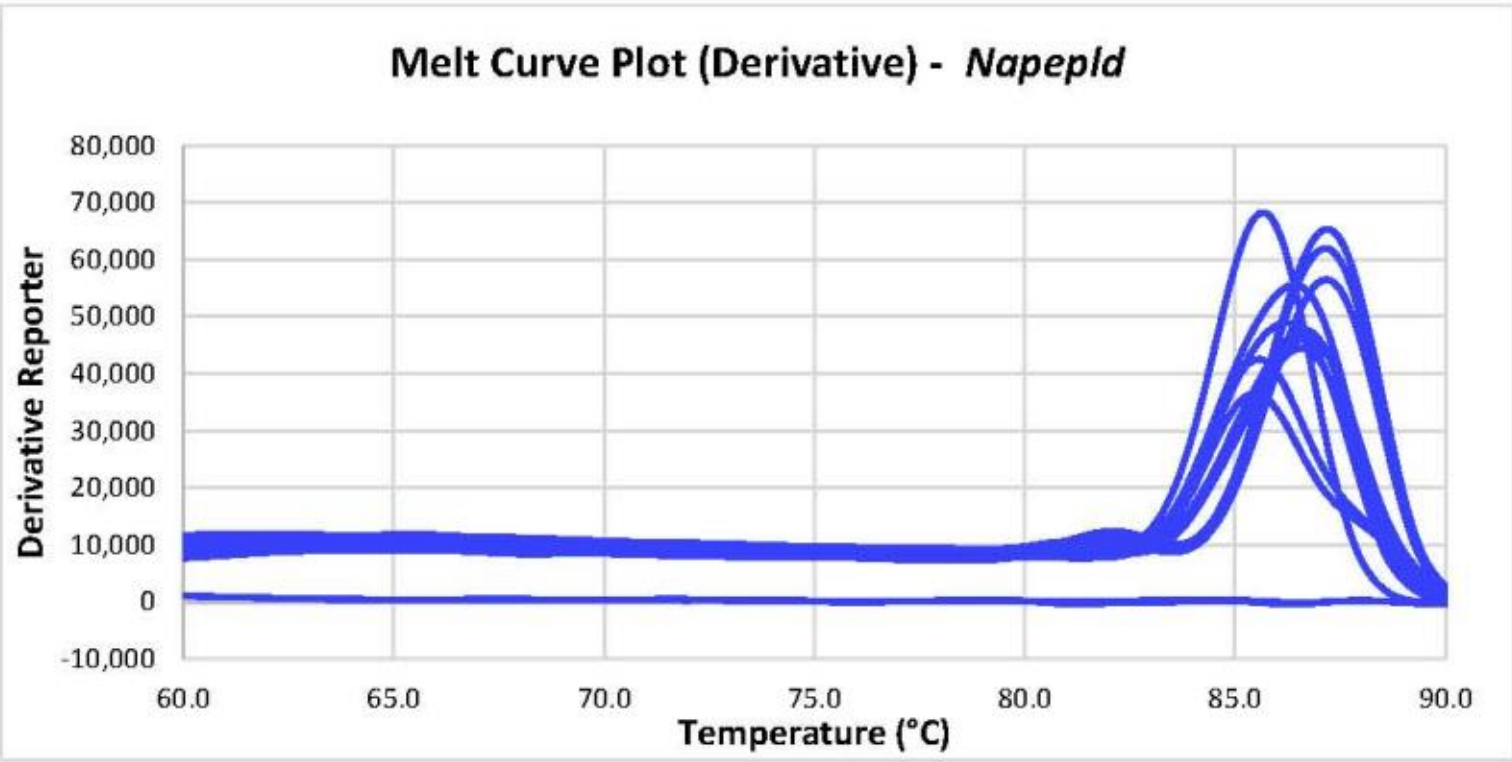

Kcnma1

Con (ng)	log(ng)	Cq
50	1.698970004	18.833
50	1.698970004	18.86
50	1.698970004	18.823
5	0.698970004	21.459
5	0.698970004	21.464
5	0.698970004	21.413
0.5	-0.301029996	24.868
0.5	-0.301029996	24.817
0.5	-0.301029996	24.704
0.05	-1.301029996	27.883
0.05	-1.301029996	28.397
0.05	-1.301029996	27.902
0.005	-2.301029996	31.876
0.005	-2.301029996	31.676

m	-3.22445
b	23.9881938
Amplification factor	2.04235113
Efficiency	104.235113

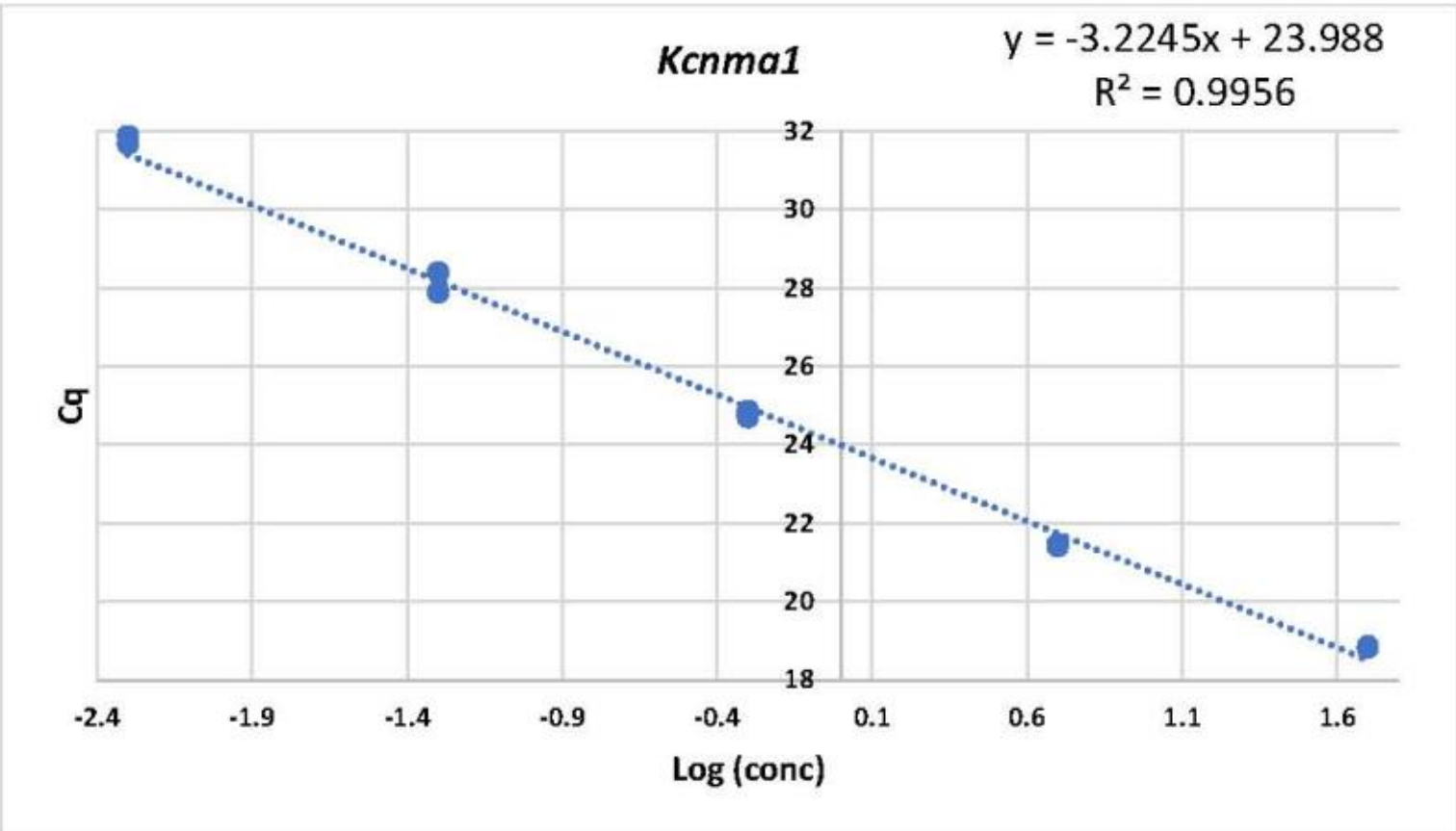

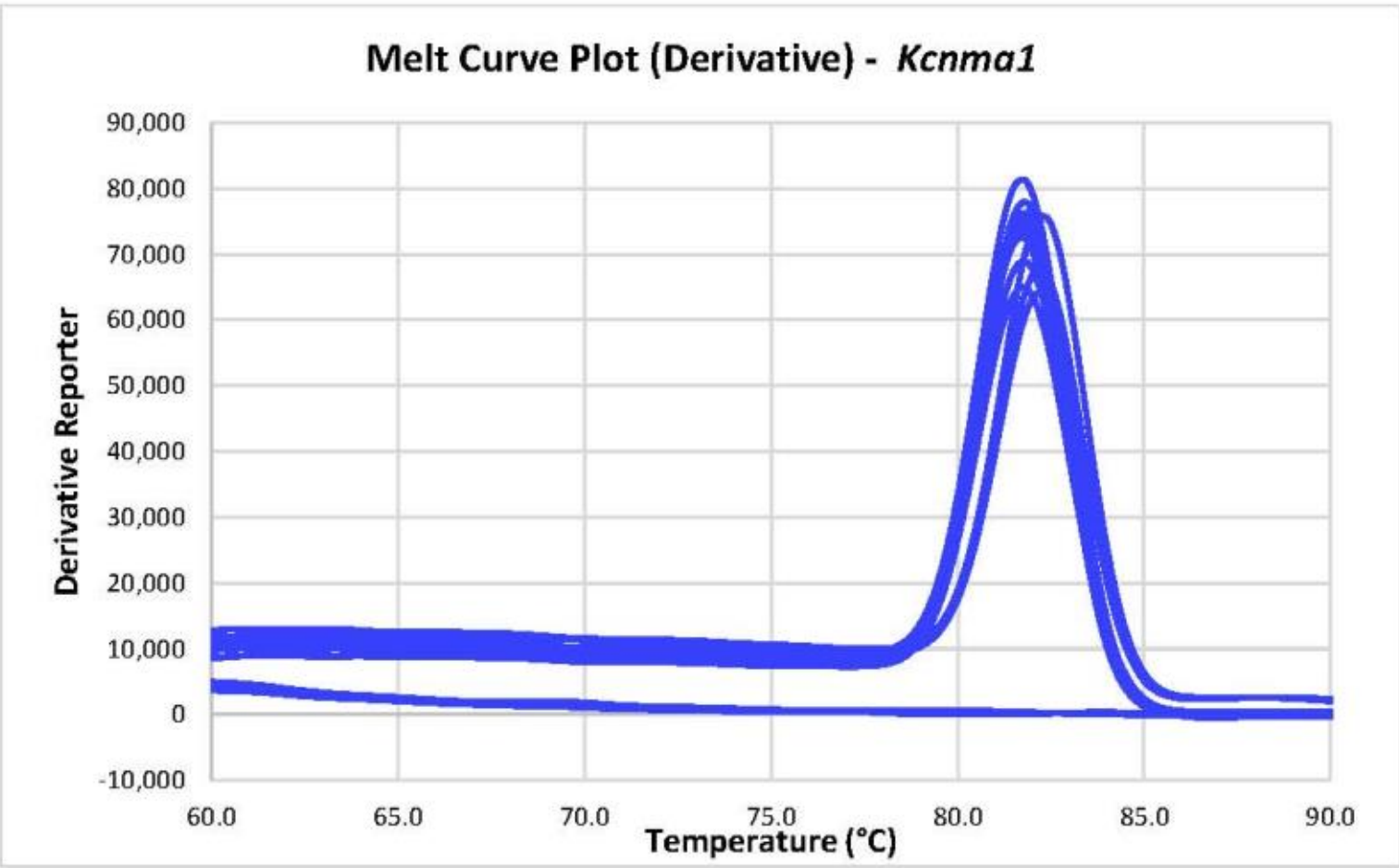

S3.4. Protein Quality Assessment.

Protein extracts obtained from the same hippocampal microdissections were quantified spectrophotometrically. Total protein concentrations ranged from

approximately 1.6 to 5.7 µg/µL depending on hippocampal region and tissue yield. Protein integrity was verified by SDS-PAGE followed by Ponceau S staining of PVDF membranes. Representative staining patterns demonstrated preservation of protein integrity and homogeneous protein transfer across samples (Supplementary **Figure S5**), supporting the suitability of the extracts for subsequent Western blot analyses.

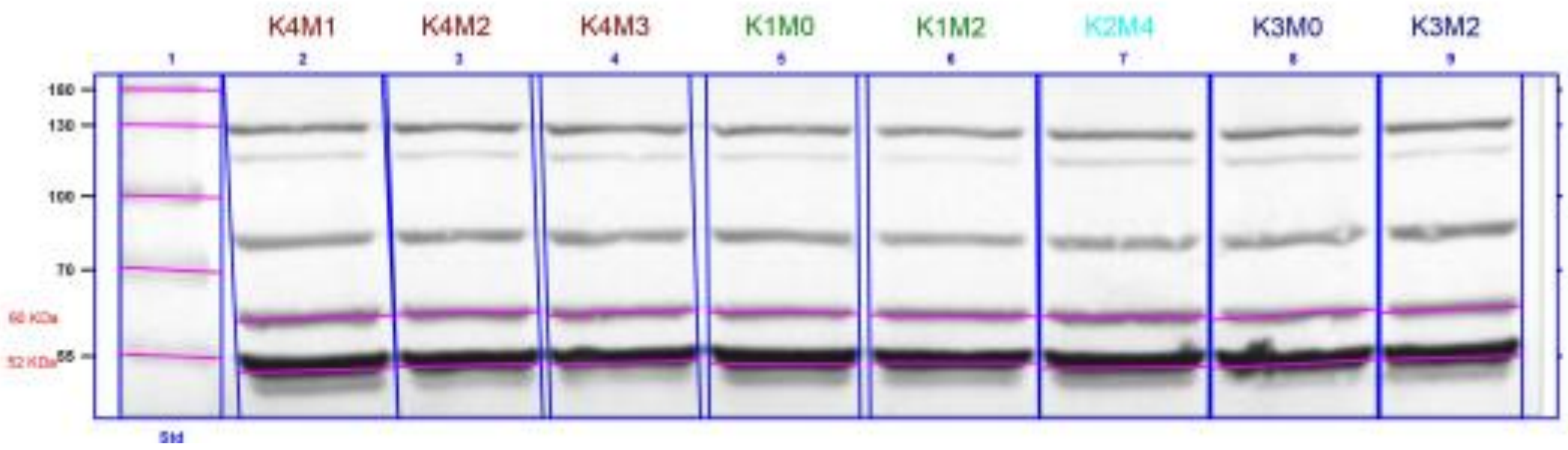

Acquisition Information

Imager	ChemiDoc MP
Exposure Time (sec)	0.146 (Optimal Auto-exposure)
Serial Number	734BR-7719
Software Version	3.0.1.14
Application	Colorimetric
Excitation Source	White Epi Illumination
Emission Filter	590/110 Filter

Image Information

Acquisition Date	31/1/2025 1:07:17 P. M.
User Name	@Lin2024
Image Area (mm)	X: 85.7 Y: 20.5
Pixel Size (µm)	X: 76.4 Y: 76.4
Data Range (Int)	23702 - 62948

Analysis Settings

Detection	Lane detection:<br>Manually created lanes<br><br>Band detection:<br>Automatically detected bands with sensitivity: High<br>Manually adjusted bands<br><br>Lane Background Subtraction:<br>Lane background subtracted with disk size: 7.4<br><br>Lane width: Variable
Mol. Weight Analysis	Standard: PageRuler™ Plus Prestained Protein Ladder 10 to 250 kDa<br>Standard lanes: first<br>Regression method: Point to Point (semi-log)

Figure S4. Representative full Western blot membrane and acquisition parameters. Representative full membrane image used for protein expression analysis. Protein bands were visualized using colorimetric detection and acquired with the ChemiDoc MP Imaging System (Bio-Rad, Hercules, CA, USA). Lanes correspond to representative samples from the experimental groups analyzed in the study. Molecular weight markers are shown on the left side of the membrane. Image acquisition was performed using automatic exposure settings, and lane detection, molecular weight assignment, and densitometric analyses were conducted using Image Lab software (version 3.0.1.14, Bio-Rad). Background subtraction was performed using the disk method, and molecular weight estimation was calculated using a Precision Plus Protein™ prestained protein ladder.

Full, uncropped membrane images are provided to ensure transparency and allow evaluation of band specificity, molecular weight assignment, and overall membrane quality. Quantitative analyses presented in the main manuscript were derived from these original images after normalization to the corresponding loading control.

S3.4. Protein quantification

Protein concentration was determined by spectrophotometry using bovine serum albumin (BSA) as the reference standard. For calibration, BSA standard solutions were prepared at concentrations of 0, 0.02, 0.04, 0.06, 0.08, and 0.10 mg/mL. The MLB (mammalian lisis buffer) solution used to dilute the samples was used as the blank to correct for absorbance attributable to the sample diluent matrix.

For each analytical session, an independent calibration curve was prepared using the BSA standard solutions. The standards and samples corresponding to each analytical session were analyzed under identical experimental and instrumental conditions. The absorbance

values obtained from the BSA standards were used to construct the calibration curve and establish the linear relationship between absorbance and the known BSA concentrations. Linear regression was then performed according to the following equation:

$$\boldsymbol{y = mx + b}$$

where *y* represents the measured absorbance, *x* represents the protein concentration (mg/mL), *m* represents the slope of the regression line, and *b* represents the y-intercept. The protein concentration of each sample was calculated from its measured absorbance using the regression equation obtained for the corresponding analytical session:

$$\boldsymbol{x = (y - b) / m}$$

For samples requiring dilution prior to spectrophotometric measurement, the corresponding dilution factor was taken into account when calculating the original protein concentration. The concentration obtained from the calibration curve represented the protein concentration in the diluted sample and was subsequently corrected according to the dilution factor using the following equation:

$$\boldsymbol{C_0 = C_\mathrm{m} \times DF}$$

Where C_0 represents the protein concentration in the original sample, C_m represents the concentration determined from the calibration curve, and *DF* is the dilution factor, calculated as the ratio between the final dilution volume and the volume of the original sample used for the dilution. Thus, the reported protein concentration corresponded to the concentration in the undiluted sample.

Example:

Protein quantification — CA1

Conc mg/ml	Abs. 1	Abs. 2	Abs. 3	Mean	Corrected Abs.
**0**	0.109	0.113	0.115	0.1123	0.000
**0.02**	0.149	0.157	0.162	0.156	0.044
**0.04**	0.204	0.199	0.205	0.203	0.090
**0.06**	0.285	0.258	0.255	0.266	0.154
**0.08**	0.31	0.311	0.316	0.312	0.200
**0.1**	0.364	0.352	0.348	0.355	0.242

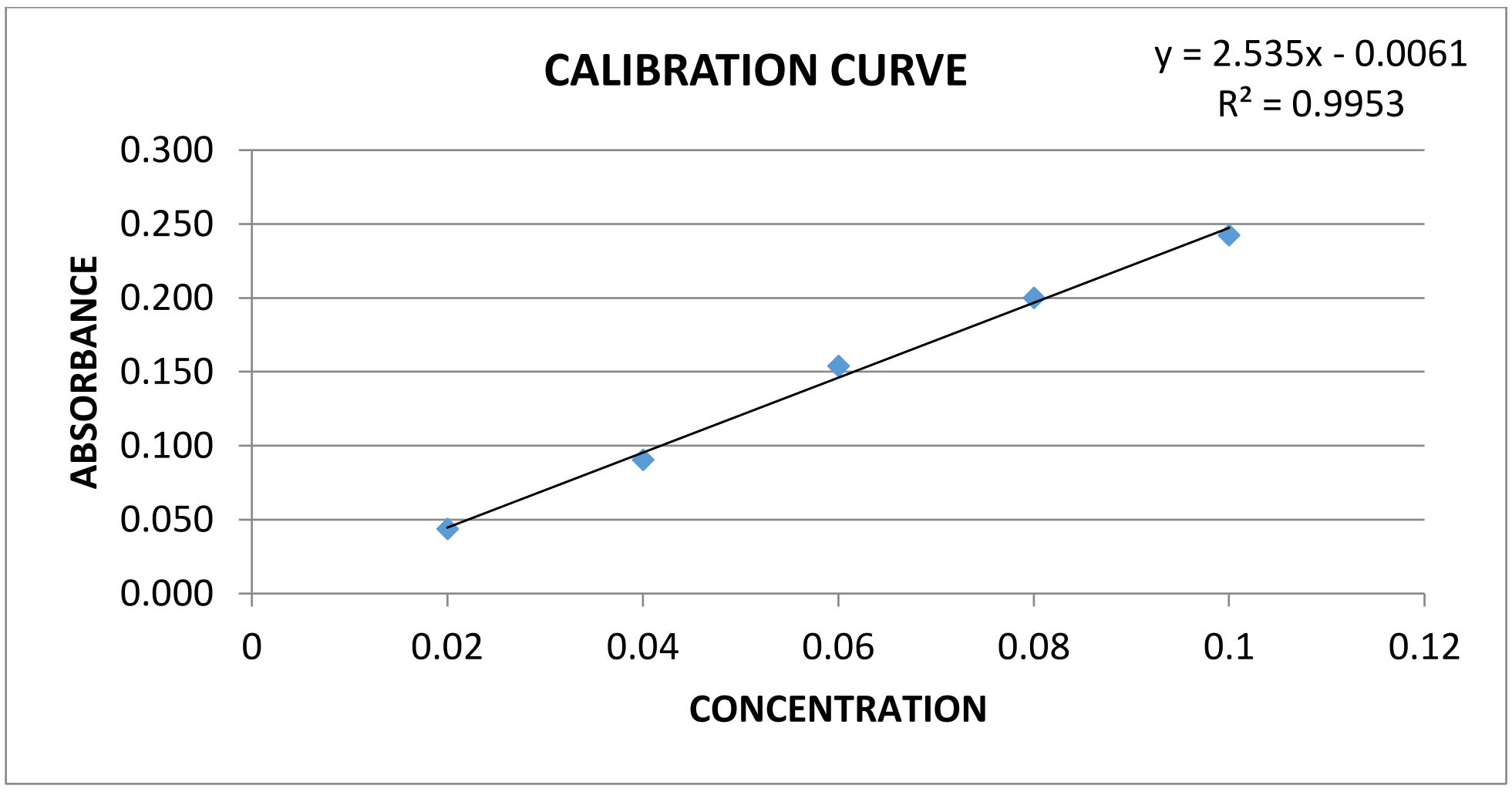

y= ***-0.0061***

m= ***2.535***

**Conc. (mg/mL)**	**MLB**	**P2M1**	**P2M2**	**P3M2**	**P3M3**	**P1M2**	**P1M3**	**P1M4**
**Abs. 1**	0.129	0.211	0.224	0.231	0.215	0.197	0.197	0.21
**Abs. 2**	0.133	0.212	0.212	0.236	0.21	0.198	0.189	0.212
**Abs. 3**	0.131	0.2115	0.218	0.2335	0.213	0.1975	0.193	0.211
**C_m**	0.0541	0.0858	0.0884	0.0945	0.0862	0.0803	0.0785	0.0856
**Normalized C_m**		0.0318	0.0343	0.0404	0.0321	0.0262	0.0245	0.0316
**C_O=Normalized C_m x DF (100)**		3.18	3.43	4.04	3.21	2.62	2.45	3.16

SECTION S4. *Behavioral sample-size and power justification*

Zare et al. (2024) and Dyomina et al. (2023) report results such as the F-test statistic and degrees of freedom in their analyses of variance, which can be converted to the η^2 effect size in the manner indicated by Lakens (2013):

$$\eta^2 = (F \times df\_effect) / (F \times df\_effect + df\_error).$$

In the present case, $\eta^2 = 0.552$ will be adopted for the Barnes maze, as it is the more conservative value and because the study by Dyomina et al. (2023), whose protocol is adapted herein, is the most closely related to the present work.

<u>BARNES</u>

Publication	Primary parameter	Statistical test	Test parameters	p-value	Interpretation in publication	Effect η
(Zare et al., 2024)	Escape latency	ANOVA	F(3,24) = 11.67	p < 0.001	Significant differences	0.593
(Dyomina et al., 2023)	Escape latency	ANOVA	F(1,17) = 21	p < 0.001	Significant differences	0.552

NOR

Publication	Primary parameter	Statistical test	Test parameters	p-value	Interpretation in publication	Effect d
(Jiang 2018)	Exploration rate	Student's t	t = 15.24	p < 0.001	Significant differences	7.002

The effect size was calculated for the results of the 24-hour NOR memory evaluation conducted in rats subjected to a pilocarpine model and in control rats, as reported by Jiang et al. (2018), on the basis of the t statistic and the sample sizes of the control and treatment groups, and following the procedure described in Lakens (2013), which directly relates these parameters to Cohen's d:

$$ds = t * \mathrm{sqrt}(1/n1 + 1/n2)$$

This is one of the studies retrieved in the search that follows a protocol similar to that employed in the present work (NOR, with a 24-hour interval between exposure to the familiar objects and the novel object) and, within this subset, the only study that reported the results of statistical tests, in addition to descriptive statistics (which were incomplete in the other studies), information that enabled the calculation of the effect size. In conclusion, d = 7.002 will be adopted.

For the following power analysis (and for other statistical analyses in this work), the (R language core Team, 2024) will be used, which presents the advantage of being released under a free license and at no cost, a factor that, together with others such as the complete description of procedures, contributes to the replicability of the studies. In this section in particular, the library pwr (Champely et al., 2017) will be used.

To begin with, the calculation of the sample size per group by means of the pwr library requires three parameters: k, the number of groups, which is four in the present work; sig.level, the significance level, set at 0.001 for both tests, as it is the most commonly used in the literature (see Tables 1 and 2); power, the statistical power, for which a value of 0.80 will be used, as it is the most widely recommended in the literature; and, finally, f, the effect size.

However, the specific function that performs the power analysis for ANOVA tests (which is intended to be used in the present work) receives effect sizes expressed as Cohen's f, which differ from those calculated and presented in Table 1 (η^2). Conversion functions between types of effect sizes are therefore required, which are provided by the effectsize library.

In the case of the Cohen's d reported in Table 2, the literature establishes the following simple relation (Cohen, 1977, p. 256):

$$f = d * 1/2$$

The instructions in the R language for the procedure described above are presented in the corresponding appendix.

Finally, the parameters and results of the preceding instructions are consolidated in the following table:

Behavioral test	***Groups (k)***	***Significance***	***Power***	***Effect size (f)***	***Estimated sample size***
Barnes maze	4	0.001	0.80	1.110	6.455
NOR	4	0.001	0.80	3.501	2.394

As can be observed, the power analysis suggests a sample size of six subjects per group (taking as a reference the higher value obtained, that of the analysis with the effect size from the Barnes maze study).

R CODE USED FOR CALCULATIONS:

```
library(effectsize)
library(pwr)
n_grupos <- 4
significancia <- 0.001
poder <- 0.80
es_etap_barnes <- 0.552
es_d_nor <- 7.002
es_f_barnes <- eta2_to_f(es_etap_barnes)
es_f_nor <- es_d_nor / 2
## power analysis
#### BARNES
pwr.anova.test(k= n_grupos, sig.level= significancia, power=0.80, f=es_f_barnes)
#### NOR:
pwr.anova.test(k= n_grupos,
sig.level= significancia,
power=0.80,
f=es_f_nor)
```

epilepsy rat mode. CNS neuroscience & therapeutics, 30(3).

https://europepmc.org/articles/PMC10912795

Supplementary Table S4. Statistical models, distributional assumptions, and sample sizes used for each experimental outcome.

Procedure	Sample size	Model	Distribution	Formula
**Kindling procedure**				
Relative body weight	Veh + Saline (n = 9), Veh + PTZ (n = 11), MCH2 + Saline (n = 9), MCH2 + PTZ (n = 10)	GLMM	Gaussian (link = "identity")	gain ~ group * experiment * day + (1 | id)
Survival probability	Veh + Saline (n = 9), Veh + PTZ (n = 11), MCH2 + Saline (n = 9), MCH2 + PTZ (n = 10)	Kaplan-Meier	Ranked	surv_obj ~ group
Racine scale progression	MCH2 + Saline (n = 9), MCH2 + PTZ (n = 10)	Cumulative Link Mixed Model	Cumulative probabilities	racine ~ group * trial + (1 | id)
Latency to first Racine S4	MCH2 + Saline (n = 9), MCH2 + PTZ (n = 10)	GLMM	Gamma	lat ~ trial * group + (1 | id)
**Novel object recognition**				
Exploration time of two familiar objects	Veh + Saline (n = 8), Veh + PTZ (n = 7), MCH2 + Saline (n = 8), MCH2 + PTZ (n = 8)	GLMM	Gamma	dur +1 ~ group * experiment * object + (1 | id)
Total exploration time (Familiar + novel)	Veh + Saline (n = 8), Veh + PTZ (n = 7), MCH2 + Saline (n = 8), MCH2 + PTZ (n = 8)	GLMM	Gaussian	total ~ group * experiment * day + (1 | id)

Exploration time of familiar vs. novel	Veh + Saline (n = 8), Veh + PTZ (n = 7), MCH2 + Saline (n = 8), MCH2 + PTZ (n = 8)	GLMM	Gamma	dur +1 ~ group * experiment * object + (1 | id)
Discrimination index	Veh + Saline (n = 8), Veh + PTZ (n = 7), MCH2 + Saline (n = 8), MCH2 + PTZ (n = 8)	GLMM	Gaussian	dis_index + 2 ~ group * experiment * day + (1 | id)
Total distance (cm)	Veh + Saline (n = 8), Veh + PTZ (n = 7), MCH2 + Saline (n = 8), MCH2 + PTZ (n = 8)	GLMM	Gaussian	vel ~ group * experiment * day + (1 | id)
Mean velocity (m/sec)	Veh + Saline (n = 8), Veh + PTZ (n = 7), MCH2 + Saline (n = 8), MCH2 + PTZ (n = 8)	GLMM	Gaussian	dist ~ group * experiment * day + (1 | id)
**Barnes**				
Latency to first goal visit	Veh + Saline (n = 8), Veh + PTZ (n = 7), MCH2 + Saline (n = 8), MCH2 + PTZ (n = 8)	GLMM	Gamma	lat ~ group * experiment * timepoint + (1 | id)

# errors until first goal visit	Veh + Saline (n = 8), Veh + PTZ (n = 7), MCH2 + Saline (n = 8), MCH2 + PTZ (n = 8)	GLMM	Gamma	errors + 1 ~ group * experiment * timepoint + (1 | id)
Distance traveled until first goal visit	Veh + Saline (n = 8), Veh + PTZ (n = 7), MCH2 + Saline (n = 8), MCH2 + PTZ (n = 8)	GLMM	Gamma	dist ~ group * experiment * timepoint + (1 | id)
Average velocity until first goal visit	Veh + Saline (n = 8), Veh + PTZ (n = 7), MCH2 + Saline (n = 8), MCH2 + PTZ (n = 8)	GLMM	Gamma	vel ~ group * experiment * timepoint + (1 | id)
Latency to goal entry	Veh + Saline (n = 8), Veh + PTZ (n = 7), MCH2 + Saline (n = 8), MCH2 + PTZ (n = 8)	GLMM	Gamma	lat ~ group * experiment * timepoint + (1 | id)
# errors until goal entry	Veh + Saline (n = 8), Veh + PTZ (n = 7), MCH2 + Saline (n = 8), MCH2 + PTZ (n = 8)	GLMM	Gamma	errors + 1 ~ group * experiment * timepoint + (1 | id)

Distance traveled until goal entry	Veh + Saline (n = 8), Veh + PTZ (n = 7), MCH2 + Saline (n = 8), MCH2 + PTZ (n = 8)	GLMM	Gamma	dist ~ group * experiment * timepoint + (1 | id)
Average velocity until goal entry	Veh + Saline (n = 8), Veh + PTZ (n = 7), MCH2 + Saline (n = 8), MCH2 + PTZ (n = 8)	GLMM	Gamma	vel ~ group * experiment * timepoint + (1 | id)
Time spent in target quadrant	Veh + Saline (n = 8), Veh + PTZ (n = 7), MCH2 + Saline (n = 8), MCH2 + PTZ (n = 8)	GLMM	Gaussian	dur_quad ~ group * experiment + (1 | id)
**RT-qPCR**				
Faah mRNA expression change in CA1	Veh + Saline (n = 6), Veh + PTZ (n = 5), MCH2 + Saline (n = 6), MCH2 + PTZ (n = 6)	GLMM	Gaussian	fc ~ group + (1 | id)
Cnr1 mRNA expression change in CA1	Veh + Saline (n = 6), Veh + PTZ (n = 5), MCH2 + Saline (n = 6), MCH2 + PTZ (n = 6)	GLMM	Gaussian	fc ~ group + (1 | id)
Napepld mRNA expression change in CA1	Veh + Saline (n = 6), Veh + PTZ (n = 5), MCH2 + Saline (n = 6), MCH2 + PTZ (n = 6)	GLMM	Gaussian	fc ~ group + (1 | id)
Kcnma1 mRNA expression change in CA1	Veh + Saline (n = 6), Veh + PTZ (n = 5), MCH2 + Saline (n = 6), MCH2 + PTZ (n = 6)	GLMM	Gaussian	fc ~ group + (1 | id)

Faah mRNA expression change in CA3	Veh + Saline (n = 6), Veh + PTZ (n = 5), MCH2 + Saline (n = 6), MCH2 + PTZ (n = 6)	GLMM	Gaussian	fc ~ group + (1 | id)
Cnr1 mRNA expression change in CA3	Veh + Saline (n = 6), Veh + PTZ (n = 5), MCH2 + Saline (n = 6), MCH2 + PTZ (n = 6)	GLMM	Gaussian	fc ~ group + (1 | id)
Napepld mRNA expression change in CA3	Veh + Saline (n = 6), Veh + PTZ (n = 5), MCH2 + Saline (n = 6), MCH2 + PTZ (n = 6)	GLMM	Gaussian	fc ~ group + (1 | id)
Kcnma1 mRNA expression change in CA3	Veh + Saline (n = 6), Veh + PTZ (n = 5), MCH2 + Saline (n = 6), MCH2 + PTZ (n = 6)	GLMM	Gaussian	fc ~ group + (1 | id)
Faah mRNA expression change in DG	Veh + Saline (n = 6), Veh + PTZ (n = 5), MCH2 + Saline (n = 6), MCH2 + PTZ (n = 6)	GLMM	Gaussian	fc ~ group + (1 | id)
Cnr1 mRNA expression change in DG	Veh + Saline (n = 6), Veh + PTZ (n = 5), MCH2 + Saline (n = 6), MCH2 + PTZ (n = 6)	GLMM	Gaussian	fc ~ group + (1 | id)
Napepld mRNA expression change in DG	Veh + Saline (n = 6), Veh + PTZ (n = 5), MCH2 + Saline (n = 6), MCH2 + PTZ (n = 6)	GLMM	Gaussian	fc ~ group + (1 | id)
Kcnma1 mRNA expression change in DG	Veh + Saline (n = 6), Veh + PTZ (n = 5), MCH2 + Saline (n = 6), MCH2 + PTZ (n = 6)	GLMM	Gaussian	fc ~ group + (1 | id)
**Western blot**				

FAAH protein expression change in CA1	Veh + Saline (n = 6), Veh + PTZ (n = 5), MCH2 + Saline (n = 6), MCH2 + PTZ (n = 6)	GLMM	Gaussian	fold_change ~ group + (1 | id)
CB1r protein expression change in CA1	Veh + Saline (n = 6), Veh + PTZ (n = 5), MCH2 + Saline (n = 6), MCH2 + PTZ (n = 6)	GLMM	Gaussian	fold_change ~ group + (1 | id)
BKa1 protein expression change in CA1	Veh + Saline (n = 6), Veh + PTZ (n = 5), MCH2 + Saline (n = 6), MCH2 + PTZ (n = 6)	GLMM	Gaussian	fold_change ~ group + (1 | id)
FAAH protein expression change in CA3	Veh + Saline (n = 6), Veh + PTZ (n = 5), MCH2 + Saline (n = 6), MCH2 + PTZ (n = 6)	GLMM	Gaussian	fold_change ~ group + (1 | id)
CB1r protein expression change in CA3	Veh + Saline (n = 6), Veh + PTZ (n = 5), MCH2 + Saline (n = 6), MCH2 + PTZ (n = 6)	GLMM	Gaussian	fold_change ~ group + (1 | id)
BKa1 protein expression change in CA3	Veh + Saline (n = 6), Veh + PTZ (n = 5), MCH2 + Saline (n = 6), MCH2 + PTZ (n = 6)	GLMM	Gaussian	fold_change ~ group + (1 | id)
FAAH protein expression change in DG	Veh + Saline (n = 4), Veh + PTZ (n = 5), MCH2 + Saline (n = 3), MCH2 + PTZ (n = 6)	GLMM	Gaussian	fold_change ~ group + (1 | id)
CB1r protein expression change in DG	Veh + Saline (n = 4), Veh + PTZ (n = 5), MCH2 + Saline (n = 3), MCH2 + PTZ (n = 6)	GLMM	Gaussian	fold_change ~ group + (1 | id)
BKa1 protein expression change in DG	Veh + Saline (n = 4), Veh + PTZ (n = 5), MCH2 + Saline (n = 3), MCH2 + PTZ (n = 6)	GLMM	Gaussian	fold_change ~ group + (1 | id)

Supplementary Table S4. Statistical models, distributional assumptions, and sample sizes used for each experimental procedure. The table summarizes the sample size, statistical model, assumed probability distribution, and model formula used for the analysis of kindling progression, behavioral outcomes, RT-qPCR, and Western blot data. GLMM, generalized linear mixed model; PTZ, pentylenetetrazol; NOR, novel object recognition; Hipocampal region CA1, CA3 and DG, dentate gyrus.

SECTION S5. *Hippocampal immunofluorescence following PTZ kindling*

NeuN, GFAP, IBA1, and CB1 were evaluated by immunofluorescence in the four experimental groups (Control, MCH-2, PTZ, and PTZ+MCH-2). NeuN was used as a neuronal marker in combination with GFAP, IBA1, and CB1 to assess their regional distribution in the dentate gyrus (DG), CA1, and CA3. Primary antibodies were used at a dilution of 1:1000 under all experimental conditions.

Scope and limitations of the immunofluorescence observations:

The representative immunofluorescence images are presented as qualitative observations of regional immunoreactivity and cellular staining profiles. No systematic quantification of fluorescence intensity, cell number, cellular density, or colocalization was performed, and no statistical comparisons were conducted for these images. Accordingly, these panels do not establish differences among experimental groups in protein abundance, astrocytic or microglial activation, or neuroinflammation, and are not used to support mechanistic conclusions or treatment effects. Such interpretations would require quantitative image analysis under standardized acquisition conditions, with defined sampling procedures and appropriate biological replication.

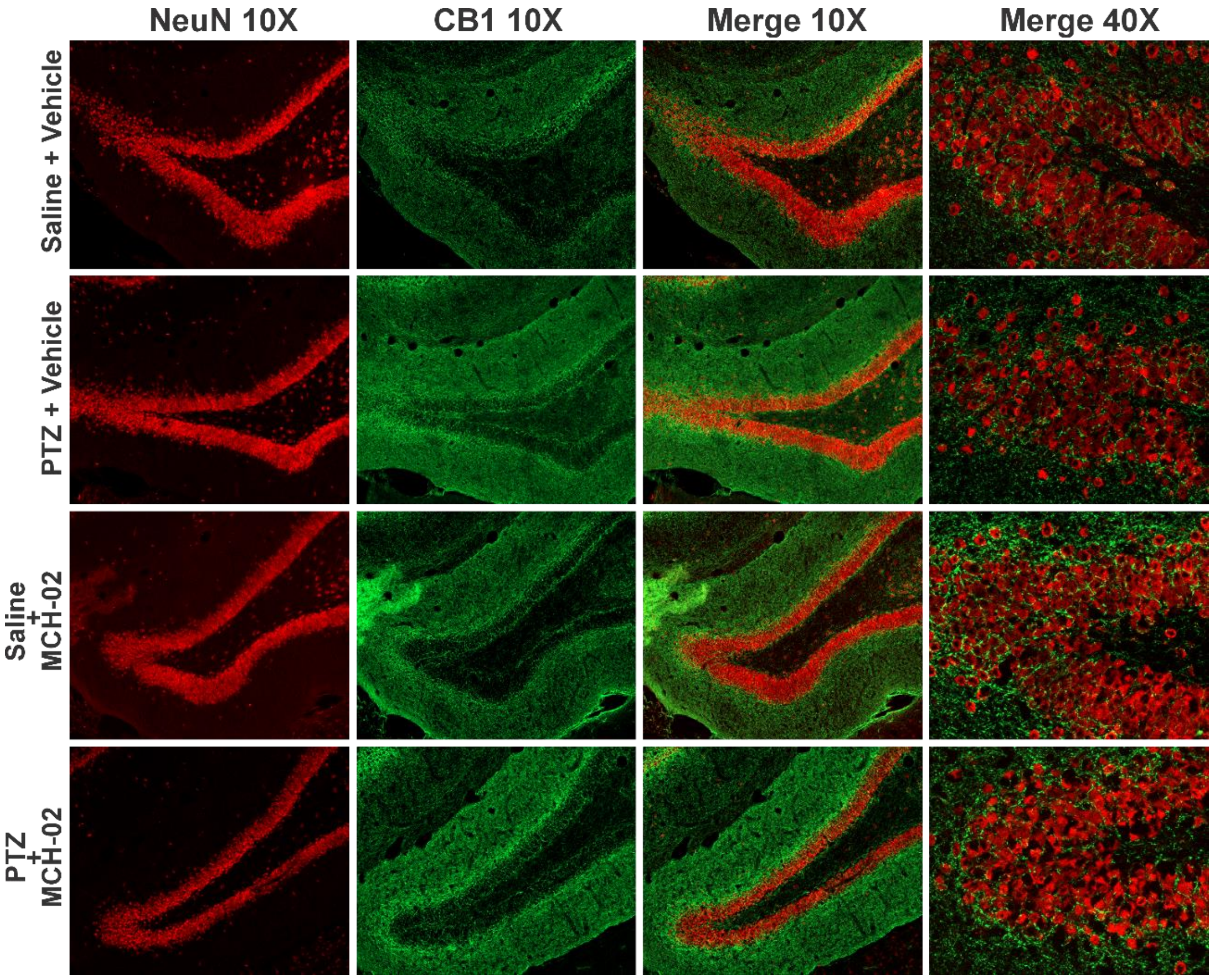

Figure S5. CB1 Immunoreactivity in the Dentate Gyrus Following PTZ Kindling. Representative immunofluorescence images showing neuronal labeling (NeuN) and cannabinoid receptor type 1 (CB1) immunoreactivity in the dentate gyrus (DG) of Control, MCH-2, PTZ, and PTZ+MCH-2 groups. Individual fluorescence channels and merged images illustrate the spatial distribution of CB1 immunoreactivity relative to NeuN-positive neuronal structures. Representative fields provide qualitative information on regional CB1 distribution and should not be interpreted independently as quantitative evidence of differences in CB1 protein expression between experimental groups.

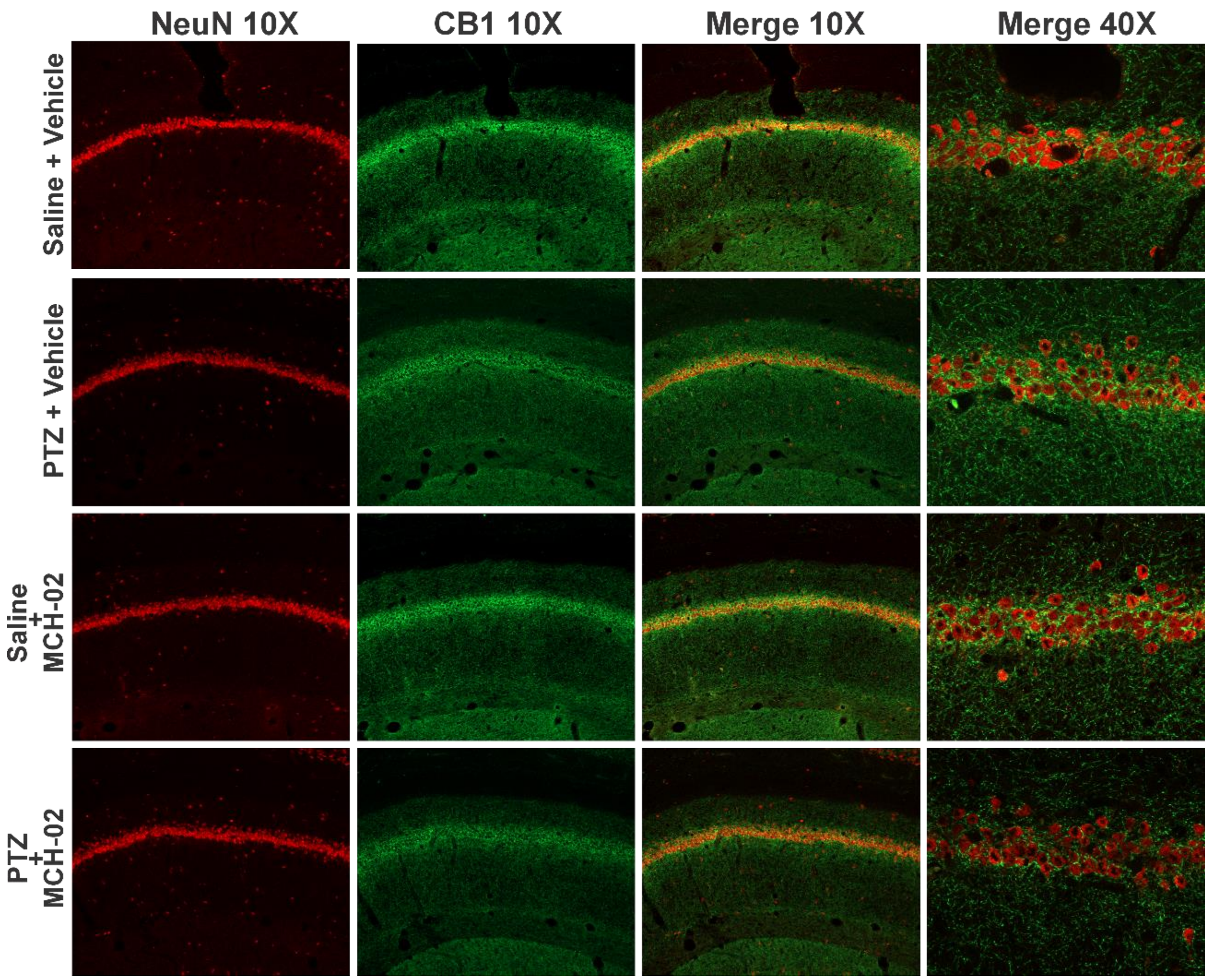

Figure S6. CB1 Immunoreactivity in the Hippocampal CA1 Region Following PTZ Kindling. Representative immunofluorescence images showing NeuN labeling and CB1 immunoreactivity in hippocampal CA1 from Control, MCH-2, PTZ, and PTZ+MCH-2 groups. Individual channels and merged images illustrate the distribution of CB1 immunoreactivity relative to NeuN-positive neuronal structures. Apparent differences in fluorescence among representative fields require quantitative analysis before conclusions regarding treatment-dependent changes in CB1 protein expression can be established.

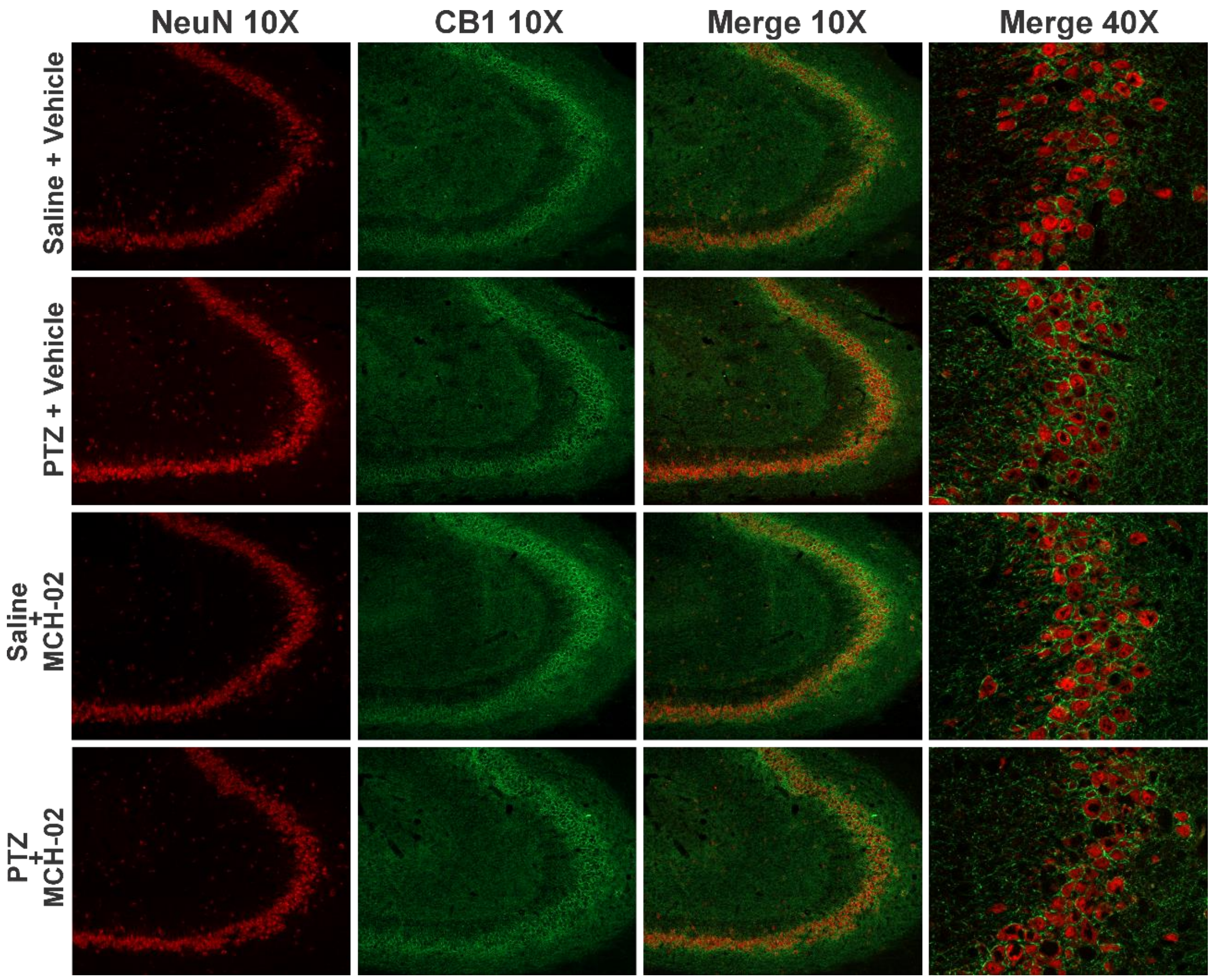

Figure S7. CB1 Immunoreactivity in the Hippocampal CA3 Region Following PTZ Kindling. Representative immunofluorescence images of NeuN and CB1 labeling in hippocampal CA3 from Control, MCH-2, PTZ, and PTZ+MCH-2 groups. Merged images illustrate the spatial relationship between CB1 immunoreactivity and NeuN-positive neuronal structures. These representative fields provide qualitative information on CB1 distribution but do not independently establish treatment-dependent changes in CB1 protein expression.

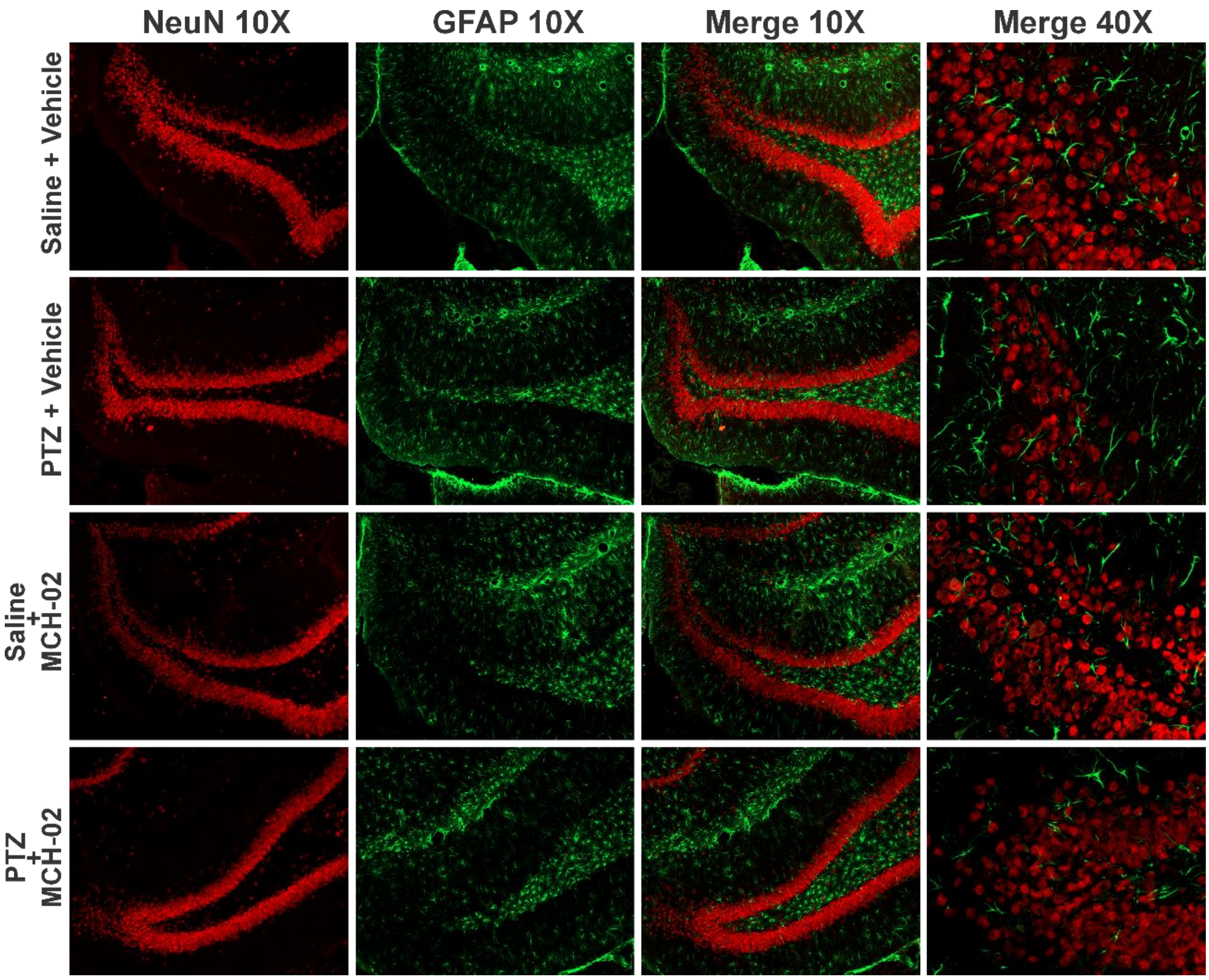

Figure S8. GFAP-Positive Astrocytic Profiles in the Dentate Gyrus Following PTZ Kindling. Representative immunofluorescence images showing NeuN labeling and glial fibrillary acidic protein (GFAP) immunoreactivity in the dentate gyrus of Control, MCH-2, PTZ, and PTZ+MCH-2 groups. GFAP-positive cellular profiles and processes are visualized within the DG, while merged images show their spatial relationship with NeuN-positive neuronal structures. Differences in GFAP immunoreactivity or astrocytic morphology among representative fields are qualitative and require morphometric and/or fluorescence-based quantification to establish treatment-associated astrocytic alterations.

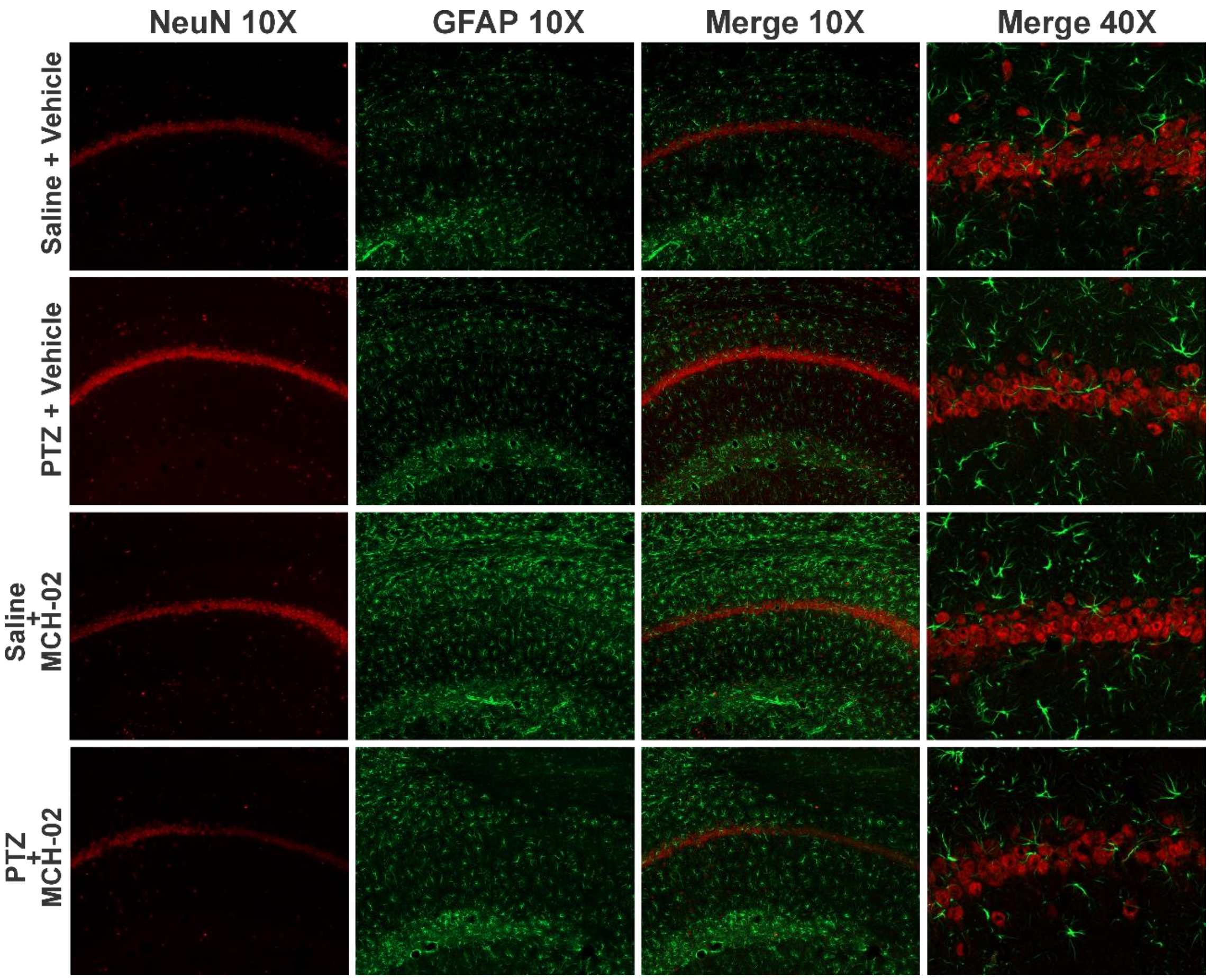

Figure S9. GFAP-Positive Astrocytic Profiles in the Hippocampal CA1 Region Following PTZ Kindling. Representative images showing NeuN and GFAP immunoreactivity in CA1 from Control, MCH-2, PTZ, and PTZ+MCH-2 groups. Individual channels and merged images illustrate the distribution and morphology of GFAP-positive astrocytic profiles relative to NeuN-positive neuronal structures. Quantitative analysis is required to determine whether GFAP immunoreactivity or astrocytic morphology differs between experimental conditions.

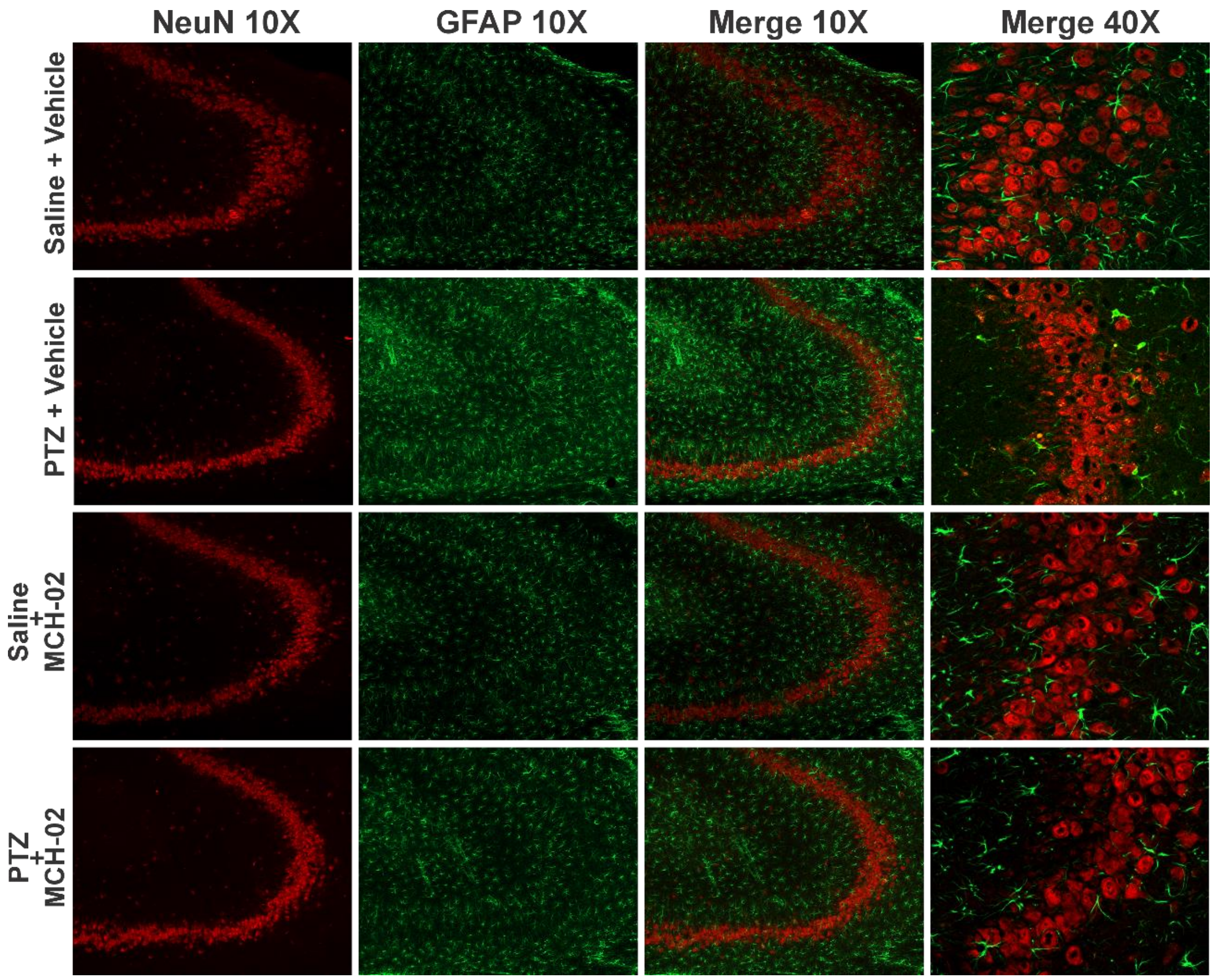

Figure S10. GFAP-Positive Astrocytic Profiles in the Hippocampal CA3 Region Following PTZ Kindling. Representative immunofluorescence images showing NeuN labeling and GFAP-positive astrocytic profiles in hippocampal CA3 from Control, MCH-2, PTZ, and PTZ+MCH-2 groups. Merged images illustrate the spatial organization of GFAP-positive cells and processes relative to NeuN-positive neuronal structures. Potential differences among groups require quantitative confirmation and should not, based on representative images alone, be interpreted as evidence of reactive astrogliosis.

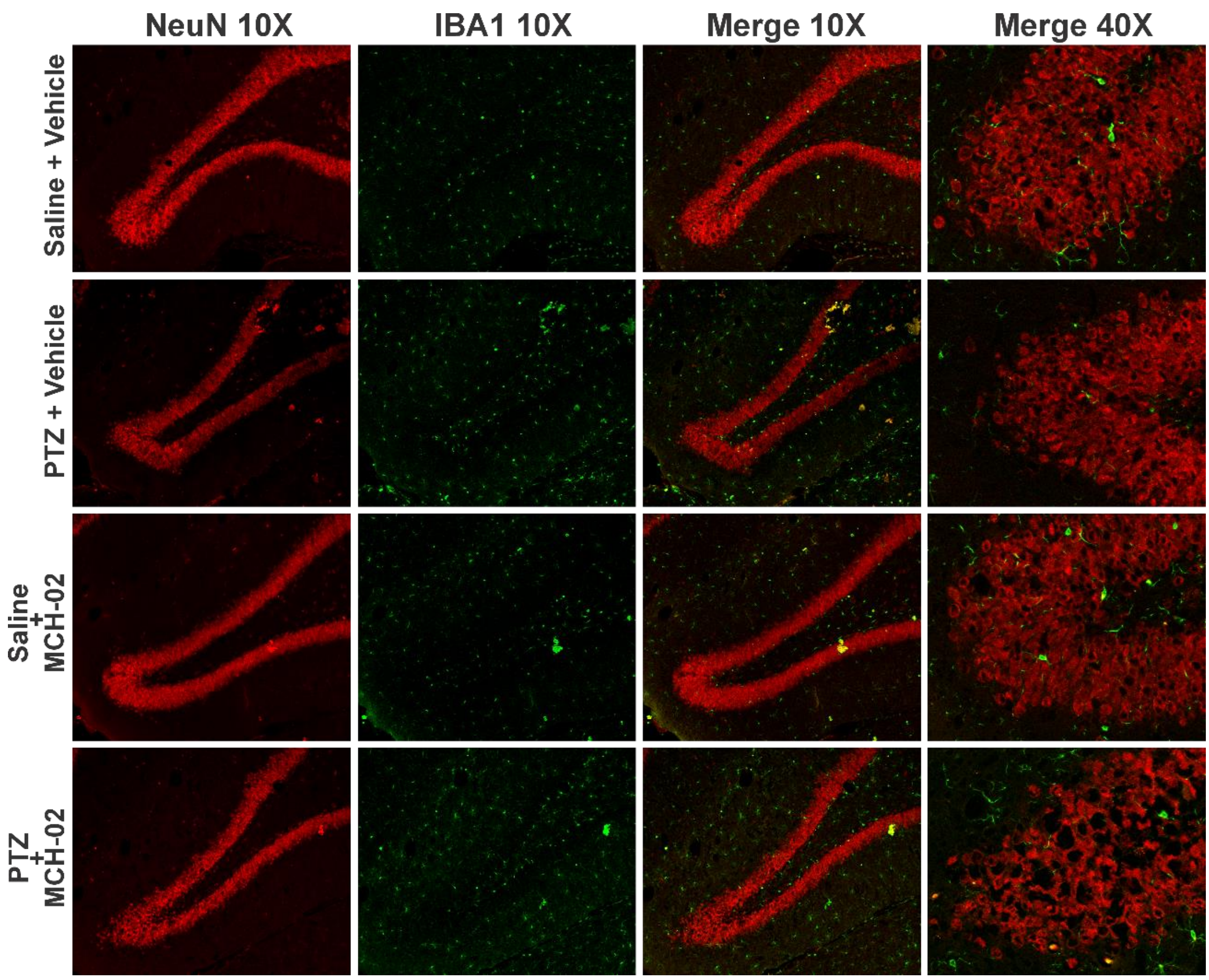

Figure S11. IBA1-Positive Microglial Profiles in the Dentate Gyrus Following PTZ Kindling. Representative immunofluorescence images showing NeuN labeling and ionized calcium-binding adaptor molecule 1 (IBA1) immunoreactivity in the dentate gyrus of Control, MCH-2, PTZ, and PTZ+MCH-2 groups. IBA1-positive microglial profiles are distributed throughout the analyzed region, and merged images illustrate their spatial relationship with NeuN-positive neuronal structures. Differences in IBA1 immunoreactivity or cellular morphology among representative fields require quantitative morphometric analysis and do not independently demonstrate microglial activation or a specific inflammatory phenotype.

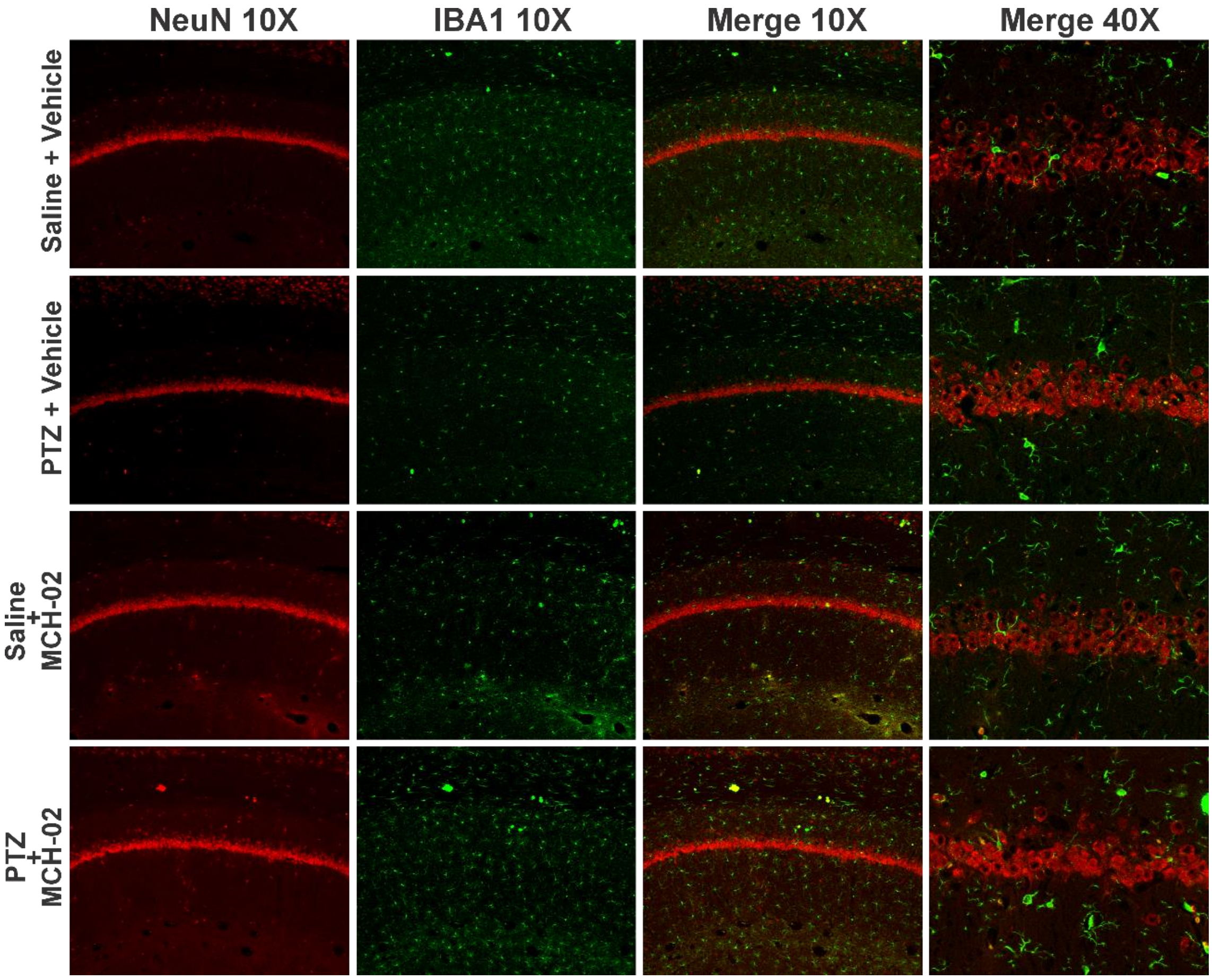

Figure S12. IBA1-Positive Microglial Profiles in the Hippocampal CA1 Region Following PTZ Kindling. Representative immunofluorescence images showing NeuN and IBA1 labeling in hippocampal CA1 from Control, MCH-2, PTZ, and PTZ+MCH-2 groups. Individual fluorescence channels and merged images illustrate the distribution and morphology of IBA1-positive microglial profiles relative to NeuN-positive neuronal structures. Qualitative differences between representative fields should be interpreted cautiously, as IBA1 immunoreactivity alone does not establish microglial activation or neuroinflammation.

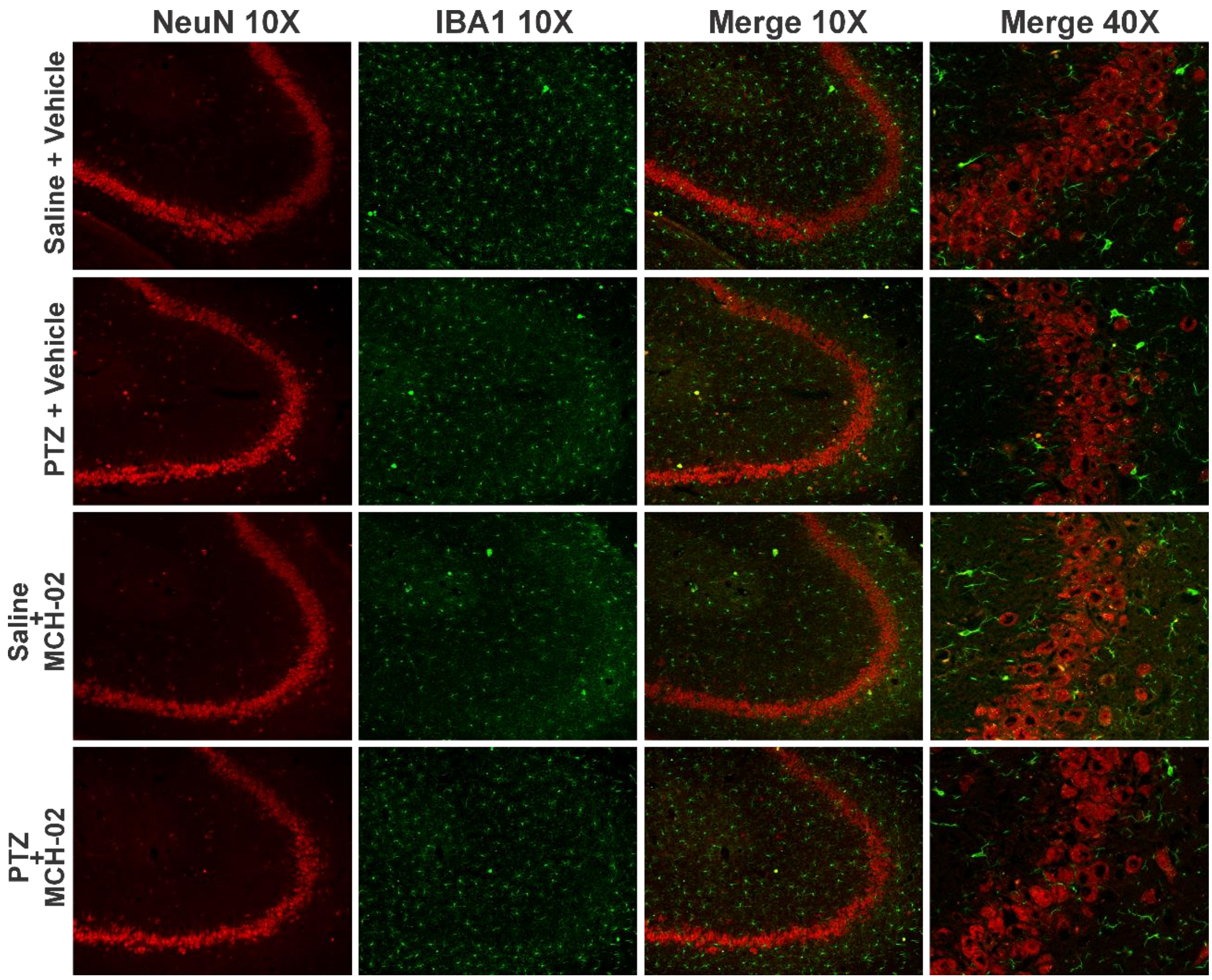

Figure S13. IBA1-Positive Microglial Profiles in the Hippocampal CA3 Region Following PTZ Kindling. Representative immunofluorescence images showing NeuN labeling and IBA1-positive microglial profiles in hippocampal CA3 from Control, MCH-2, PTZ, and PTZ+MCH-2 groups. Merged images illustrate the spatial distribution of IBA1-positive cells relative to NeuN-positive neuronal structures across the experimental conditions. These representative images provide qualitative information regarding microglial distribution and morphology; quantitative morphometric and fluorescence analyses are required to establish treatment-dependent microglial alterations.

Bibliography

Ben-Shachar, M. S., Lüdecke, D., & Makowski, D. (2020). Effectsize: Estimation of effect size indices and standardized parameters. Journal of Open Source Software, 5(56), 2815. https://doi.org/10.21105/joss.02815

Cohen, J. (1977) Statistical Power Analysis for the Behavioral Sciences. Revised Edition, Academic Press, New York.

Dyomina, A. V., Smolensky, I. V., & Zaitsev, A. V. (2023). Refinement of the Barnes and Morris water maze protocols improves characterization of spatial cognitive deficits in the lithium-pilocarpine rat model of epilepsy. Epilepsy & behavior, 147, 109391. https://doi.org/10.1016/j.yebeh.2023.109391

Jiang, Y., Han Chun-Lei, Liu Huan-Guang, Wang, X., Zhang, X., Meng Fan Gang, & Zhang Jian-Guo. (2018). Abnormal hippocampal functional network and related memory impairment in pilocarpine treated rats. Epilepsia, 59(9), 1785–1795. https://doi.org/10.1111/epi.14523

Lakens, D. (2013). Calculating and reporting effect sizes to facilitate cumulative science: a practical primer for t-tests and ANOVAs. Frontiers in psychology, 4. https://doi.org/10.3389/fpsyg.2013.00863
R Core Team (2024) R: A Language and Environment for Statistical Computing. R Foundation for Statistical Computing. https://www.R-project.org/

Zare, M., Rezaei, M., Nazari, M., Kosarmadar, N., Faraz, M., Barkley, V., Shojaei, A., Raoufy, M. R., & Mirnajafi-Zadeh Javad. (2024). Effect of the closed-loop hippocampal low-frequency stimulation on seizure severity, learning, and memory in pilocarpine
