# Supplementary material for: MCH-02, a synthetic macamide analog with in vitro FAAH-inhibitory activity, modulates hippocampal endocannabinoid system and BK channel expression without preventing PTZ-induced progressive seizure susceptibility in rats
Source: Front Neurol. 2026 Oct 16;17:1843264. doi: 10.3389/fneur.2026.1843264 (PMC13719350; doi:10.3389/fneur.2026.1843264)
Supplement: Supplementary file 2 [file Data_Sheet_2.PDF]

Table 1													
Quantification of RNA and Total Protein from Rat Hippocampal Microdissections (CA1)													
**Animal ID**	**Experimental Group**				**Integrity test**	**Reverse transcription**				**Total protein**			
		**Concentration (ng/µL)**	**Total volume (µL)**	**A260/A280**	**Volume q.s. to 400 ng (µL)**	**RNA volume for reverse transcription (µL)**	**Volume q.s. to 500 ng (µL)**	**Final cDNA volume (25 ng/µL)**		**Concentration (ug/µL)**	**Initial volume (µL)**	**Final concentration (ug/µL)**	**Final volume (µL)**
**P3M0**	Veh+PTZ	114.5	17.0	1.77	3.5	12.5	4.4	57.3		2.40	65.0	2.00	78.08
**P4M1**	Veh+PTZ	174.0	17.0	1.87	2.3	10.6	2.9	73.8		3.91	68.0	2.00	132.81
**P4M2**	Veh+PTZ	173.5	17.0	1.86	2.3	10.7	2.9	74.3		4.06	68.0	2.00	138.13
**P4M4**	Veh+PTZ	118.5	17.0	1.83	3.4	12.6	4.2	59.8		3.09	68.0	2.00	104.92
**K2M4**	Veh+PTZ	167.2	17.0	1.92	2.4	13.6	3.0	76.0		2.37	62.0	2.00	73.47
**P5M0**	MCH-02 + PTZ	129.8	17.0	1.68	3.1	12.9	3.9	67.1		3.16	65.0	2.00	102.83
**P5M1**	MCH-02 + PTZ	129.9	17.0	1.82	3.1	12.9	3.8	67.1		3.16	68.0	2.00	107.58
**P5M2**	MCH-02 + PTZ	144.0	17.0	1.84	2.8	12.8	3.5	73.7		3.50	68.0	2.00	118.87
**P5M3**	MCH-02 + PTZ	124.9	17.0	1.84	3.2	11.8	4.0	59.0		1.99	66.4	1.60	82.68
**K1M0**	MCH-02 + PTZ	138.4	17.0	1.88	2.9	13.1	3.6	76.0		2.49	66.5	2.00	82.90
**K1M2**	MCH-02 + PTZ	181.2	17.0	1.93	2.2	13.8	2.8	76.0		2.97	63.0	2.00	93.64
**K4M1**	MCH-02 + Saline	182.0	17.0	1.90	2.2	13.8	2.7	76.0		3.33	62.0	2.00	103.19
**K4M2**	MCH-02 + Saline	165.5	17.0	1.92	2.4	13.6	3.0	76.0		2.90	65.0	2.00	94.38
**K4M3**	MCH-02 + Saline	153.2	17.0	1.88	2.6	13.4	3.3	76.0		2.23	64.5	2.00	72.01
**P1M2**	MCH-02 + Saline	138.5	17.0	1.81	2.9	13.1	3.6	72.6		2.62	65.0	2.00	85.26
**P1M3**	MCH-02 + Saline	115.7	17.0	1.78	3.5	11.5	4.3	53.2		2.45	65.0	2.00	79.49
**P1M4**	MCH-02 + Saline	203.1	17.0	1.84	2.0	14.0	2.5	114.0		3.16	65.0	2.00	102.56
**P2M1**	Veh+Saline	121.0	17.0	1.78	3.3	11.7	4.1	56.6		3.18	65.0	2.00	103.21
**P2M2**	Veh+Saline	161.0	17.0	1.80	2.5	13.5	3.1	87.0		3.43	65.0	2.00	111.54
**P3M2**	Veh+Saline	158.2	17.0	1.82	2.5	13.5	3.2	85.2		4.04	65.0	2.00	131.41
**P3M3**	Veh+Saline	153.3	17.0	1.84	2.6	13.4	3.3	82.1		3.21	65.0	2.00	104.49
**K3M0**	Veh+Saline	158.6	17.0	1.90	2.5	13.5	3.2	76.0		2.52	65.5	2.00	82.55
**K3M2**	Veh+Saline	184.1	17.0	1.92	2.2	13.8	2.7	76.0		3.27	65.0	2.00	106.40

Table 2														
**Quantification of RNA and Total Protein from Rat Hippocampal Microdissections (CA3)**														
**Animal ID**	**Experimental Group**				**Integrity test**	**Reverse transcription**				**Total protein**				
		**Concentration (ng/µL)**	**Total volume (µL)**	**A260/A280**	**Volume q.s. to 400 ng (µL)**	**RNA volume for reverse transcription (µL)**	**Volume q.s. to 500 ng (µL)**	**Final cDNA volume (25 ng/µL)**		**Concentration (ng/µL)**	**Initial volume (µL)**	**Final concentration (ng/µL)**	**Final volume (2 µg/µL)**	
**P3M0**	Veh+PTZ	142.6	17	1.77	2.81	13.2	3.5	75.3		1.56	61.00	1.50	63.32	
**P4M1**	Veh+PTZ	165.5	17	1.75	2.42	13.6	3.0	76.0		1.78	61.00	1.60	67.93	
**P4M2**	Veh+PTZ	151.8	17	1.77	2.64	13.4	3.3	76.0		2.17	62.00	2.00	67.17	
**P4M4**	Veh+PTZ	165.7	17	1.80	2.41	13.6	3.0	76.0		1.93	61.00	1.60	73.43	
**K2M4**	Veh+PTZ	159.3	17	1.90	2.51	13.5	3.1	76.0		2.52	62.50	2.00	78.71	
**P5M0**	MCH-02 + PTZ	180.7	17	1.81	2.21	13.8	2.8	76.0		2.33	62.00	2.00	72.15	
**P5M1**	MCH-02 + PTZ	168.3	17	1.82	2.38	13.6	3.0	76.0		2.49	60.00	2.00	74.64	
**P5M2**	MCH-02 + PTZ	156.1	17	1.79	2.56	13.4	3.2	76.0		2.50	59.00	2.00	73.87	
**P5M3**	MCH-02 + PTZ	121.4	17	1.78	3.29	12.7	4.1	76.0		1.80	59.00	1.70	62.39	
**K1M0**	MCH-02 + PTZ	272.9	17	1.94	1.47	14.5	1.8	76.0		2.64	62.00	2.00	81.86	
**K1M2**	MCH-02 + PTZ	239.6	17	1.90	1.67	14.3	2.1	76.0		2.62	62.50	2.00	81.88	
**K4M1**	MCH-02 + Saline	179.0	17	1.91	2.23	13.8	2.8	76.0		1.95	65.50	1.60	79.82	
**K4M2**	MCH-02 + Saline	265.6	17	1.93	1.51	14.5	1.9	76.0		2.06	64.50	2.00	66.49	
**K4M3**	MCH-02 + Saline	298.6	17	1.95	1.34	14.7	1.7	76.0		3.22	62.00	2.00	99.80	
**P1M2**	MCH-02 + Saline	105.8	17	1.76	3.78	12.2	4.7	51.7		3.84	63.00	2.00	120.93	
**P1M3**	MCH-02 + Saline	91.9	17	1.70	4.35	11.6	5.4	42.8		3.36	64.00	2.00	107.40	
**P1M4**	MCH-02 + Saline	136.6	17	1.80	2.93	13.1	3.7	71.4		3.03	63.00	2.00	95.59	
**P2M1**	Veh+Saline	142.3	17	1.82	2.81	12.2	3.5	69.4		2.00	64.00	1.30	98.46	
**P2M2**	Veh+Saline	130.8	17	1.80	3.06	12.9	3.8	67.7		3.29	64.00	2.00	105.20	
**P3M2**	Veh+Saline	115.6	17	1.76	3.46	11.5	4.3	53.2		2.51	64.00	1.70	94.33	
**P3M3**	Veh+Saline	123.1	17	1.79	3.25	12.8	4.1	62.8		2.90	64.00	1.90	97.57	
**K3M0**	Veh+Saline	189.6	17	1.92	2.11	13.9	2.6	76.0		2.92	62.50	2.00	91.40	
**K3M2**	Veh+Saline	171.3	17	1.88	2.34	13.7	2.9	76.0		2.19	64.40	2.00	70.64	

**Table 3**													
**Quantification of RNA and Total Protein from Rat Hippocampal Microdissections (DG)**													
**Animal ID**	**Experimental Group**				**Integrity test**	**Reverse transcription**				**Total protein**			
		**Concentration (ng/µL)**	**Total volume (µL)**	**A260/A280**	**Volume q.s. to 400 ng (µL)**	**RNA volume for reverse transcription (µL)**	**Volume q.s. to 500 ng (µL)**	**Final cDNA volume (25 ng/µL)**		**Concentration (ug/µL)**	**Initial volume (µL)**	**Final concentration (ug/µL)**	**Final volume (2µL)**
**P3M0**	Veh+PTZ	151.5	17	1.81	2.64	13.4	3.3	81.0		2.5444	65.00	2.00	82.69
**P4M1**	Veh+PTZ	151.3	17	1.84	2.64	13.4	3.3	80.8		3.3136	62.00	2.00	102.72
**P4M2**	Veh+PTZ	162.2	17	1.81	2.47	13.5	3.1	87.8		2.9389	64.00	2.00	94.04
**P4M4**	Veh+PTZ	160.7	17	1.81	2.49	13.5	3.1	86.8		3.7278	62.00	2.00	115.56
**K2M4**	Veh+PTZ	229.3	17	1.88	1.74	14.3	2.2	76.0		1.9831	64.50	1.90	67.32
**P5M0**	MCH-02 + PTZ	173.0	17	1.86	2.31	13.7	2.9	94.7		5.7791	66.00	2.00	190.71
**P5M1**	MCH-02 + PTZ	186.9	17	1.84	2.14	13.9	2.7	103.6		5.0099	67.00	2.00	167.83
**P5M2**	MCH-02 + PTZ	130.9	17	1.86	3.06	12.9	3.8	67.8		4.3195	66.00	2.00	142.54
**P5M3**	MCH-02 + PTZ	199.0	17	1.89	2.01	14.0	2.5	111.3		4.7732	64.00	2.00	152.74
**K1M0**	MCH-02 + PTZ	311.5	17	1.94	1.28	14.7	1.6	76.0		3.3755	65.50	2.00	110.55
**K1M2**	MCH-02 + PTZ	219.9	17	1.90	1.82	14.2	2.3	76.0		2.9255	65.00	2.00	95.08
**K4M1**	MCH-02 + Saline	190.8	17	1.92	2.10	13.9	2.6	76.0		1.9831	61.00	1.90	63.67
**K4M2**	MCH-02 + Saline	226.3	17	1.93	1.77	14.2	2.2	76.0		2.7145	65.00	2.00	88.22
**K4M3**	MCH-02 + Saline	284.2	17	1.93	1.41	14.6	1.8	76.0		2.5176	64.00	2.00	80.56
**P1M2**	MCH-02 + Saline	198.5	17	1.87	2.02	14.0	2.5	111.0		0.5720	53.00	0.40	75.79
**P1M3**	MCH-02 + Saline	201.8	17	1.86	1.98	14.0	2.5	113.2		0.8679	52.00	0.70	64.47
**P1M4**	MCH-02 + Saline	158.9	17	1.86	2.52	13.5	3.1	85.7		1.2623	58.00	1.00	73.21
**P2M1**	Veh+Saline	178.3	17	1.88	2.24	13.8	2.8	98.1		0.4734	51.00	0.30	80.47
**P2M2**	Veh+Saline	154.2	17	1.85	2.59	13.4	3.2	82.7		0.4536	54.00	0.30	81.66
**P3M2**	Veh+Saline	172.2	17	1.87	2.32	13.7	2.9	94.2		-0.1183	54.00	---	---
**P3M3**	Veh+Saline	119.0	17	1.83	3.36	12.6	4.2	60.2		0.2367	58.00	0.15	91.52
**K3M0**	Veh+Saline	227.4	17	1.90	1.76	14.2	2.2	76.0		1.9269	65.00	1.80	69.58
**K3M2**	Veh+Saline	298.1	17	1.95	1.34	14.7	1.7	76.0		3.6709	65.50	2.00	120.22
									**C2M0***	3.60	66.00	2.00	118.87
									**C2M2***	2.55	66.00	2.00	84.21

Samples in red were not used for subsequent analyses

* *Due to the limited availability of biological material for protein expression analysis, two additional control samples were included exclusively for Western blot analysis. Their inclusion was restricted to this analysis and did not alter the group allocation used for the other experimental assessments. These samples were processed under the same experimental conditions and following the same protocol as the other samples included in the protein analysis.*

Note: *Differences in the final cDNA volume among samples reflect variations in the amount of RNA available for reverse transcription. For samples with final volumes different from 76 µL, the reverse transcription reaction was performed using the entire available RNA volume. For samples with a final volume of 76 µL, the amount of RNA subjected to reverse transcription was adjusted to obtain the specified final volume. Importantly, the subsequent calculations accounted for these volume differences and ensured normalization of the cDNA cotration across samples prior to downstream analys*
