# Supplementary material for: MCH-02, a synthetic macamide analog with in vitro FAAH-inhibitory activity, modulates hippocampal endocannabinoid system and BK channel expression without preventing PTZ-induced progressive seizure susceptibility in rats
Source: Front Neurol. 2026 Oct 16;17:1843264. doi: 10.3389/fneur.2026.1843264 (PMC13719350; doi:10.3389/fneur.2026.1843264)
Supplement: Supplementary file 3 [file Data_Sheet_3.PDF]

Group		Kindling protocol		* Behavioral assessments		Molecular analyses**		Inmunofluorescense
**Veh + Saline**	9 (6 / 3)	Completed kindling	8	NOR and Barnes	8	RT-qPCR CA1, CA3 and DG	6	2
		Mortality	1			WB CA1, CA3 and DG	6 (CA1 and CA3)<br>4 DG***	
**Veh + PTZ**	11 (6 / 5)	Completed kindling	7	NOR and Barnes	7	RT-qPCR CA1, CA3 and DG	5	2
		Mortality	4			WB CA1, CA3		
**MCH-02 + Saline**	9 (5 / 4)	Completed kindling	8	NOR and Barnes	8	RT-qPCR CA1, CA3	6	2
		Mortality	1			WB CA1, CA3 and DG	6 (CA1 and CA3)<br>3 DG***	
**MCH-02 + PTZ**	10 (5 / 5)	Completed kindling	8	NOR and Barnes	8	RT-qPCR CA1, CA3 and DG	6	2
		Mortality	2			WB CA1, CA3 and DG		
**Total**	**39**	**39**		**31**		**23**		**8**

*** Behavioral assessments:** *The same animals were used for both the Novel Object Recognition (NOR) and Barnes maze tests.*

**** Molecular analyses:** *The same animals that underwent the behavioral assessments were subsequently used for molecular analyses, including RT-qPCR (CA1, CA3, and DG) and Western blot (CA1, CA3 and DG).*

****** *The final sample size varied among the DG qPCR analyses because only samples with adequate protein concentration were included.*

.

Details

Group	Animal	Cohort	Kindling	Mortality	NOR	Barnes	RT-qPCR (CA1/CA3/DG)	WB CA1	WB CA3	WB DG	Inmunofluorescense
Veh + Saline	P2M0	1	-	Just after kindling	---	---	---	---	---	---	---
	P2M1	1	-	-	YES	YES	YES	YES	YES	NO	NO
	P2M2	1	-	-	YES	YES	YES	YES	YES	NO	NO
	P3M1	1	-	-	YES	YES	NO	NO	NO	NO	YES
	P3M2	1	-	-	YES	YES	YES	YES	YES	NO	NO
	P3M3	1	-	-	YES	YES	YES	YES	YES	NO	NO
	K3M0	2	-	-	YES	YES	YES	YES	YES	YES	NO
	K3M1	2	-	-	YES	YES	NO	NO	NO	NO	YES
	K3M2	2	-	-	YES	YES	YES	YES	YES	YES	NO
Veh + PTZ	P3M0	1	Completed	-	YES	YES	YES	YES	YES	YES	NO
	P4M0	1	Stopped D1	Day 1	---	---	---	---	---	---	---
	P4M1	1	Completed	-	YES	YES	YES	YES	YES	YES	NO
	P4M2	1	Completed	-	YES	YES	YES	YES	YES	YES	NO
	P4M3	1	Completed	-	YES	YES	NO	NO	NO	NO	YES
	P4M4	1	Completed	-	YES	YES	YES	YES	YES	YES	NO
	K2M0	2	D0	Challenge	---	---	---	---	---	---	---
	K2M1	2	D0	Challenge	---	---	---	---	---	---	---
	K2M2	2	D0	Challenge	---	---	---	---	---	---	---
	K2M4	2	Completed	-	YES	YES	YES	YES	YES	YES	NO
	K2M5	2	Completed	-	YES	YES	NO	NO	NO	NO	YES
MCH-02 + Saline	P1M0	1	Completed	-	YES	YES	NO	NO	NO	NO	YES
	P1M1	1	D9	Day 9	---	---	---	---	---	---	---
	P1M2	1	Completed	-	YES	YES	YES	YES	YES	NO	NO
	P1M3	1	Completed	-	YES	YES	YES	YES	YES	NO	NO
	P1M4	1	Completed	-	YES	YES	YES	YES	YES	NO	NO
	K4M0	2	Completed	-	YES	YES	NO	NO	NO	NO	YES
	K4M1	2	Completed	-	YES	YES	YES	YES	YES	YES	NO
	K4M2	2	Completed	-	YES	YES	YES	YES	YES	YES	NO
	K4M3	2	Completed	-	YES	YES	YES	YES	YES	YES	NO
MCH-02 + PTZ	P5M0	1	Completed	-	YES	YES	YES	YES	YES	YES	NO
	P5M1	1	Completed	-	YES	YES	YES	YES	YES	YES	NO
	P5M2	1	Completed	-	YES	YES	YES	YES	YES	YES	NO
	P5M3	1	Completed	-	YES	YES	YES	YES	YES	YES	NO
	P5M4	1	Completed	-	YES	YES	NO	NO	NO	NO	YES
	K1M0	2	Completed	-	YES	YES	YES	YES	YES	YES	NO
	K1M1	2	Completed	-	YES	YES	NO	NO	NO	NO	YES
	K1M2	2	Completed	-	YES	YES	YES	YES	YES	YES	NO
	K1M3	2	Challenge	Challenge	---	---	---	---	---	---	---
	K1M4	2	Challenge	Challenge	---	---	---	---	---	---	---
